# Brain substrates of visual scene memory: a lesion-behavior mapping study

**DOI:** 10.3389/fnhum.2025.1606051

**Published:** 2025-09-01

**Authors:** Shir Ben-Zvi Feldman, Nachum Soroker, Daniel A. Levy

**Affiliations:** ^1^Faculty of Medicine, Tel Aviv University, Tel Aviv, Israel; ^2^Baruch Ivcher School of Psychology, Reichman University, Herzliya, Israel; ^3^Loewenstein Rehabilitation Medical Center, Ra'anana, Israel; ^4^Department of Psychology, Palo Alto University, Palo Alto, CA, United States

**Keywords:** visual-scene memory, recall, stroke, lesion, Wechsler Memory Scale

## Abstract

**Background:**

The brain network supporting visual-scene memory includes ventral (what) and dorsal (where, how) visual processing streams, and hubs within the medial temporal cortex, plus frontal, temporal, and parietal regions comprising the core-recollection memory system. However, the exact relationship between these regions and the capacity to memorize different types of visual-scene elements remains debated. Functional neuroimaging studies point to dissociable as well as common network components supporting perception and memory of different aspects of visual information. In the current neuropsychological study, we assess impact of stroke lesion topography on recall of identity, location, and action of event participants, as assessed by the *WMS-III Family Pictures* subtest.

**Methods:**

Ninety-three first-event stroke patients (54 and 39 right- and left-hemisphere damaged, respectively; RHD, LHD) in the sub-acute phase performed the Family Pictures task. Voxel-based lesion-symptom mapping (VLSM) analysis identified brain lesions implicated in memory deficits for the composite score and separately for each scene element: characters' identity, the action they performed, and location of occurrence.

**Results:**

Behavioral analysis revealed significant impairment in identity, location, and action memory among right hemisphere damage (RHD) patients compared to matched healthy controls. Performance was significantly lower in delayed compared to immediate testing condition, and memory domains showed a hierarchy of scores: highest for identity, intermediate for location, and lowest for action. VLSM revealed a markedly different pattern of lesion effects in the two hemispheres. In the RH, network was dominated by large voxel clusters in middle and superior temporal and inferior parietal regions. In contradistinction, the LH network was dominated by large voxel clusters in temporo-occipital and medial temporal lobe (MTL) regions. VLSM conjunction analysis disclosed further distinctions between anatomical regions subserving memory of character identity, action, and location.

**Discussion:**

Visual scene memory is supported by a bi-hemispheric network dominated in the LH by temporo-occipital/MTL structures and in the RH by temporo-parietal components of the core recollection network. The LH network regions were mostly implicated in a non-specific manner, whereas in the RH network more regions were implicated in memory for specific scene elements.

## 1 Introduction

Our visual experience of the world is an ongoing stream of distinct features of events unfolding simultaneously, such as the identity of the persons who take part in an episode, their relative positions, their intentions, and their actions, converging into a coherent and seemingly unified representation (Grieben et al., 2020; Morales-Torres et al., 2024). Visual episodic memories for complex scenes are crucial for everyday orientation in the world, social interactions, and even our ability to simulate future scenarios and plan ahead (Schacter et al., 2017). During episodic remembering, we recollect multiple perceptual features tied to an event that occurred in a specific spatio-temporal context (Clewett et al., 2019; Ranganath, 2010). These features can range from high-level identities and actions to fundamental visual attributes such as the perceived lightness of colors (Bortolotti et al., 2022). Notably, recent work suggests that visual recognition memory for scenes is predominantly driven by higher-level, categorical, rather than low-level sensory, visual representations (Morales-Torres et al., 2024). Visual episodic memories of scenes are a particularly complex form of episodic memory as they can also include meaningful activities and social interactions, which are especially important in ecological contexts. Such memories require the binding of multiple distinct perceptual features (Ranganath, 2010), including participants' identity, executed actions, and spatial locations, as bound components of a single experienced event within a specific spatiotemporal context.

What are the anatomical substrates of our memory abilities for such observed events? While it is likely that medial temporal lobe (MTL) regions implicated in declarative memory in general (Squire and Dede, 2015) are required, additional neural substrates may contribute to memory for the various types of information represented in the visual scene (Morales-Torres et al., 2024; Gallivan and Goodale, 2018). While both neuroimaging and lesion studies have provided insights into visual scene memory (as reviewed below), systematic understanding of how brain lesions differentially affect memory for distinct visual scene elements remains limited.

Given that remembering might involve the recapitulation of neural activities occurring at the time of data acquisition, i.e., when perceiving the occurrence of the original event (Johnson et al., 2009; Staresina et al., 2012; Wing et al., 2015), we might expect that remembering the identity of visual-scene items would require reactivation of temporo-occipital, ventral visual processing stream areas (the “what pathway”) of the visual association cortex implicated in recognition of objects and persons (Morales-Torres et al., 2024). In contrast, remembering the location of visual-scene items would be based on parieto-occipital, dorsal-stream areas (the “where pathway”) of the visual association cortex, implicated in representation of the spatial location of visual objects (Torres-Morales and Cansino, 2024; Gallivan and Goodale, 2018). Functional imaging studies have indeed confirmed the reactivation of ventral-stream representations in the context of episodic memory (Xue, 2018), with recall of objects being consistently associated with activation patterns across the fusiform and parahippocampal gyri (Schacter et al., 1995; Vaidya et al., 2002; Wheeler et al., 2000; Wheeler and Buckner, 2003; Slotnick et al., 2003). Other regions activated during visual object identity memory were frontal regions and the precuneus (Slotnick et al., 2003; Wheeler et al., 2000), lateral temporal regions (Slotnick et al., 2003), and middle occipital and lateral parietal regions (Wheeler et al., 2000). In a recent fMRI study, Morales-Torres et al. (2024) have demonstrated the neural underpinnings of object identity memory within visual scenes using a four-alternative forced-choice test. Performance related to activation in a network of regions spanning the occipital, parietal, and temporal lobes reliably predict subsequent memory for naturalistic stimuli. However, some studies have demonstrated cortical activation related to visual item memory in both dorsal and ventral visual streams (Bone et al., 2020; Buchsbaum et al., 2012; Moscovitch et al., 1995; Slotnick et al., 2003; Wheeler et al., 2000). Furthermore, a lesion study by Kessels et al. (2000) reported left parietal and occipital as well as right medial and posterior parietal involvement in object identity memory.

Other studies have focused on memory for the spatial location of objects. A typical spatial-location paradigm entails the presentation of visual objects in various locations in a grid, in which participants are tested subsequently for their memory of the objects and their original locations. Early positron emission tomography (PET) studies reported regional cerebral blood flow increments associated selectively with location memory in different right hemisphere regions—middle occipital and supramarginal gyri and the superior temporal sulcus region. This pattern contrasted sharply with bilateral activations in the fusiform and lingual gyri associated selectively with object identity memory. Consistent with the well-established role of the dorsal visual stream in spatial processing and guiding attention and action within visual scenes, Grieben et al. (2020) demonstrated that visual scene memory significantly enhances the speed and efficiency of visual search through mechanisms involving spatial inhibition, a cognitive process that strongly implicates dorsal stream contributions. However, a recent systematic review and meta-analysis by Torres-Morales and Cansino (2024) identified a spatial retrieval network comprising regions from both the dorsal (e.g., superior parietal lobule and precuneus) and ventral (e.g., inferior temporal and parahippocampal gyri) visual streams. This integrated engagement presumably reflects the notion that spatial information isn't processed independently of its associated object or event (Torres-Morales and Cansino, 2024). While these domain-specific activation patterns likely represent a continuum of processing in perception and memory systems, other regions of the cortical mantle exhibited domain-general activations with distinctive patterns shown for the encoding and retrieval phases (Köhler et al., 1998; Moscovitch et al., 1995). Subsequently, various functional magnetic resonance imaging (fMRI) studies corroborated the distinction between neural substrates involved in item memory vs. source or context memory (i.e., the processing involved in recognition memory of an object's identity vs. the memory of its spatial location). For example, Slotnick et al. (2003) reported on right and left prefrontal activations related to the retrieval of identity and location information, respectively. In a later study (Ross and Slotnick, 2008), retrieval of location information was associated with stronger activations in the hippocampus and parahippocampal cortex compared to retrieval of item identity information. Memory of both item identity and item location was associated with widespread activations in different regions of the dorsolateral cortical mantle with partial overlap between the two (Ross and Slotnick, 2008). Hales and Brewer (2013) reported on distinctive parietal and frontal fMRI activation patterns upon attempted vs. successful association of object stimuli and corresponding spatial-location cues. Lesion studies showed that the capacity to remember the location of specific objects is negatively affected by damage to the posterior parietal cortex (Kessels et al., 2000), especially in the left hemisphere (van Asselen et al., 2009). Another lesion study by Lugtmeijer et al. (2021) examined in 81 stroke patients the impact of lesion topography on performance of a working memory task, and afterwards on a surprise episodic memory task, where patients had to judge whether the items shown in the working memory task are now presented in the same or in a different location. Results point to shared and distinct processes in the two tasks, localizing at different cortical and sub-cortical structures of the right hemisphere.

Literature reviews of lesion and functional imaging studies (Postma et al., 2008; Zimmermann and Eschen, 2017) pointed to an MTL (especially the hippocampus) role in binding object identity and location data. Object-location memory was also found to be impaired following damage to fronto-parietal cortical regions, with categorical and coordinate (exact) object-location binding being affected more by left- and right-hemispheric lesions, respectively. In comparison to studies of the impact of focal lesions, the functional imaging studies reviewed by Zimmermann and Eschen (2017) pointed to a larger extent of the involved neural networks, implicating additional brain regions in the process of binding an object to its exact location in episodic memory. These authors stress the importance of distinguishing between different facets of object-location memory, activated under different experimental conditions [i.e., incidental vs. intentional encoding; short- vs. long-term maintenance; implicit vs. explicit retrieval; object-location binding within the context of a specific event (episodic memory) vs. knowing the location of objects as part of semantic memory; object location encoded in small vs. large environment, in egocentric vs. allocentric reference frames, or in categorical (more abstract) vs. coordinate (more concrete and exact) spatial terms]. All these factors seem to exert differential effects, which are still far from being clear, on fMRI activation patterns associated with object-location memory (Zimmermann and Eschen, 2017).

Actions in the context of events are represented and interpreted with reference to conceptual schemas, which have a profound impact on how those actions are later remembered (Alba and Hasher, 1983; Gilboa and Marlatte, 2017). Knowledge structures that specify the sequence of actions in a stereotyped event are called scripts (Abelson, 1981; Abbott et al., 1985; Hudson et al., 1992). For example, in a restaurant script, there is a sequence of typical activities: being seated, getting a menu, ordering food, eating, and paying (Hudson et al., 1992). Stored scripts guide the interpretation and integration into memory of experienced events. With respect to human actions, the literature points to a possible contribution of mirror-neuron cell aggregates near the temporo-parietal junction in processing biological movement at the perceptual level, from which the observer derives a direct insight into the underlying intentionality, behavioral goals, and social meaning of perceived human actions (Gallese et al., 2004). Empirical evidence, gathered mainly in functional neuroimaging research, points also to a specific role of the sensorimotor system in perceptual processing and maintenance in memory of dynamic bodily actions (for a review see Galvez-Pol et al., 2020). Further processing and interpretation of gestures and actions performed by others in a social context involves the medial prefrontal cortex (mPFC), where the meaning and intentionality behind such actions can be deduced by reference to more expanded contextual information stored in memory following past experiencing of comparable events (van Overwalle, 2009). Recent fMRI studies point to a different involvement of mPFC and MTL networks in modulation and enhancement of event memory, where the former region seems to affect memory through congruence with past knowledge while the latter through incongruence with past knowledge (e.g., Bonasia et al., 2018; Masís-Obando et al., 2022).

Studies of memory for specific features of objects and events and their neural underpinnings generally examine retrieval of each feature separately. Relatively little work has been conducted in which subjects were shown complex visual scenes portraying daily events, and then were examined for memory of specific details in the scene. One such study, by Burgess et al. (2001), used event-related fMRI to explore the neural substrates associated with memory of different event details. During the encoding phase, participants received various objects from different people in different places within a virtual reality environment. Participants were then tested for their memory of the objects, the persons who handled the objects, and the locations where this happened. The experiment evoked extended fMRI activation patterns, both in medial (precuneus; parahippocampal gyri; left hippocampus; anterior cingulate) and lateral (posterior parietal and lateral prefrontal) aspects of the cortical mantle. Bilateral PFC activations during retrieval of both types of context information (the person giving the item and the location of the delivery) was thought to indicate a strategic role in retrieval for these regions. In contrast, a network of regions that included the parahippocampal gyri, right posterior parietal cortex, and the precuneus was specifically related to location memory.

Two studies which investigated the neural substrates of memory for different event details in an autobiographical episodic memory context are also worth noting. An fMRI study by Gilmore et al. (2021) demonstrated separable systems in the memory network supporting explicit recall of people, places, and objects in experienced past events. Recall of peoples' details was associated with bilateral enhanced activations in the ventromedial prefrontal cortex, posterior cingulate cortex, anterior temporal lobes, and the posterior superior temporal sulcus—angular gyrus region. Recall of place details was associated with bilateral enhanced activations in the parahippocampal cortex, posterior angular gyrus, and retrosplenial cortex/parieto-occipital sulcus. Recall of objects' details was associated with broad activations including the lateral occipital complex and dorsal-parietal, somatosensory and prefrontal cortices. A study by Memel et al. (2020) used diffusion MRI to investigate the neural substrate of autobiographical episodic retrievals of people/objects (event elements) details as compared to spatiotemporal (event context) details. Memory for event elements was found to relate to hippocampal-cortical interactions subserved by the uncinate fasciculus, which connects medial temporal lobe (MTL) structures with the anterotemporal network (including orbitofrontal, temporopolar, amygdala and adjacent regions). In contrast, memory for spatiotemporal context details was found to relate to hippocampal-cortical interactions subserved by the cingulum bundle, which connects MTL structures with the posteromedial network (including the precuneus, retrosplenial cortex, posterior cingulate, inferolateral parietal cortex, medial prefrontal cortex and the parahippocampal cortex). It should be noted however that transmission through the cingulum bundle was related to recollection of both element and context details of autobiographical events.

A recent neuroimaging study by Morales-Torres et al. (2024), has advanced the understanding of visual recognition memory for scene data by showing that categorical features of high-level vision, processed within the precuneus, inferior temporal and superior occipital cortices, rather than features of low-level vision, reliably predict subsequent memory for naturalistic stimuli. This emphasis on higher-level processing in scene memory formation is particularly pertinent to our current study, which implicitly assumes categorical processing of visual scene data.

It should be noted that usually, neuroimaging studies of visual memory examine retrieval of specific types of memoranda following a separate presentation. Relatively little work has been conducted in which subjects were shown complex visual scenes portraying daily events and then were examined for memory of specific details in the scene (e.g., Burgess et al., 2001; Gilmore et al., 2021). Neuroimaging findings reveal complex patterns of dissociable substrates for object, location, and action information (e.g., Schacter et al., 1995; Vaidya et al., 2002; Wheeler et al., 2000; Wheeler and Buckner, 2003; Slotnick et al., 2003). Furthermore, while these studies provide valuable insights into the neural networks supporting visual scene memory in healthy brains, they cannot establish whether these regions are necessary for memory functions. Lesion studies, on the other hand, can shed light on causal relationships between specific brain regions and memory functions. The few existing lesion studies that have examined memory for visual objects of different types, for the location of objects or for other attributes of data presented visually, usually addressed each type of data separately rather than as distinct elements of a unified scene or episode (e.g., Kessels et al., 2000; van Asselen et al., 2009; Lugtmeijer et al., 2021). This approach limits our understanding of how brain lesions differentially affect memory for distinct elements (identity, location, action) within integrated visual scenes, and prevents direct comparison of component-specific neural substrates. Therefore, the current neuropsychological study aims to examine how lesions—specifically, those engendered by stroke—affect memory for each category of memoranda in the *Family Pictures* subtest of the WMS-III test using a lesion-symptom mapping analysis.

The *Family Pictures* test is one of the mandatory subtests of the third version of the WMS (WMS-III; Wechsler, 1997). It assesses visual memory for complex meaningful pictures in immediate and delayed recall phases. In this task, participants view drawings presenting different episodes in family life, which include persons, each in a certain location, performing a specific action. Participants are tested for their memory of each of those aspects of the visual scene. The *Family Pictures* subtest is widely used as a diagnostic tool and was found to show high sensitivity to deficits in different patient groups. For example, patients who underwent right temporal lobectomy were found to perform the *Family Pictures* test post-operatively at a lower level relative to patients who underwent similar surgery on the left hemisphere (Chapin et al., 2009; Doss et al., 2004; Wilde et al., 2001). Recently, Reekes et al. (2023) found a significant difference between males and females in patients with Parkinson's disease in *Family Pictures* delayed recall performance. This task was also used as a measure of deficits in memory for social episodes among autistic persons (Williams et al., 2005). A few studies have manipulated the standard scoring method of the *Family Pictures* test to create measures for different phenomena. For example, to increase the sensitivity of this task to laterality effects while assessing individuals who had undergone temporal lobectomies, Holley et al. (2000) divided the *Family Pictures* subtest into Character, Location, and Action components. After statistically removing verbal memory scores, they observed that the Location score demonstrated increased sensitivity to structural damage in the right temporal lobe. In another study, to create a measurement for feature binding in schizophrenic patients, Gold et al. (2004) calculated the average number of features recalled per successfully recalled character by summing the location, action, and object scores and dividing by the number of characters recalled.

In the current study we employ systematic group analyses of lesion effects by voxel-based lesion symptom mapping (VLSM) to examine how stroke lesion topography impacts this assessment. In addition to scrutinizing the neuroanatomical correlate of visual scene memory as reflected in the original composite score of the WMS-III *Family Pictures* subtest (resulting from summation of the scores for identity, location and action of characters), we used VLSM conjunction analysis to uncover non-specific shared neural substrates related to all the three components of the composite score, vs. neural substrates which are specifically linked to recall of just one or two of the visual scene elements.

By applying VLSM analysis to Family Pictures components, we adopt an exploratory approach with three specific predictions. First, we expect to identify both shared and component-specific neural substrates: damage to domain-general memory/perception networks will show non-selective impact on all three components, while damage to regions engaged in domain-specific processing will affect selectively identity, location or action components of a visual scene. Second, based on the dual-stream theory, we predict that damage to ventral stream regions will affect mainly the retrieval of actors' identity in the scene, whereas damage to dorsal stream regions will preferentially affect location and action recall. Third, we anticipate hemispheric specialization, with right hemisphere regions showing stronger associations with spatial components compared to left hemisphere regions. The precise magnitude and spatial extent of these dissociations will be determined empirically through our systematic VLSM approach.

This information is expected to shed new light on the involvement of distinct hubs of the recollection network in recall of distinct elements of a visual scene. This in turn may contribute to refining the utility of the *Family Pictures* test in clinical neuropsychology, allowing for a better characterization of specific difficulties in remembering different types of memoranda, in their relation to the location of brain damage in individual stroke patients.

## 2 Methods

### 2.1 Participants

Ninety-three patients with first-incident ischemic or hemorrhagic hemispheric stroke in the subacute phase were recruited for the study during their hospitalization at the Loewenstein Rehabilitation Medical Center (LRMC), Raanana, Israel, or under treatment in the neurology department at Wolfson Medical Center (WMC), Holon, Israel.

#### 2.1.1 Image acquisition

Structural brain imaging information (CT/MRI scans) analyzed in this study was acquired for diagnostic and follow-up clinical purposes in different clinical settings, using different scanners and acquisition protocols, but with slice thickness of 5 mm or less in all cases. To ensure comparability of lesion analyses, all images underwent rigorous post-acquisition standardization and spatial normalization procedures as detailed in the “Lesion Analysis” section.

#### 2.1.2 Participant recruitment and selection

Potential participants were identified from individuals admitted to these facilities. An initial screening based on medical records verified eligibility against primary inclusion criteria (first-incident, subacute phase, ischemic or hemorrhagic stroke with unilateral hemispheric damage). The “first-incident” nature of the stroke was confirmed by careful review of each patient's medical records, careful evaluation of the imaging data, and questioning the patient and family about past occurrence of stroke symptoms and signs. These measures confirmed that the recent stroke was a first-ever event. The type of stroke and unilaterality were further confirmed via CT/MRI scans. This initial screening also assessed exclusion criteria (prior psychiatric or neurological disorders, regular psychotropic drug use prior to the stroke, unstable clinical/metabolic state). Importantly, only patients who were medically stable and deemed capable of reliably participating in comprehensive neuropsychological evaluations were included in the study. Patients passing this preliminary review were then comprehensively assessed by treating speech and occupational therapists using the clinical tests described below to confirm their language and cognitive capacity for study participation. All participants had unilateral hemispheric lesions and were assigned to either the right hemisphere damage (RHD) or left hemisphere damage (LHD) group based on the location of the lesion, as determined from clinical CT/MRI scans and documented in the medical record. Analyses were conducted separately for the RHD and LHD groups. Specifically for the purpose of this study, which focuses on performance of the “family pictures” subtest of the WMS-III battery, five LHD patients were identified who, in view of their performance on other subtests of the WMS-III, were judged to be incompetent for reliably performing the “family pictures” task in a manner enabling any clear conclusions with respect to their scene memory. These patients were consequently not included in the final participant cohort. Only those fulfilling all criteria and providing informed consent were finally recruited, resulting in a total of 93 participants (54 RHD and 39 LHD). The diversity of patient lesion profiles and the consequences of such variance for cognition seems to belie the normality distribution assumptions required for standard power estimates. We therefore focused on recruiting the maximum number of patient participants who met inclusion and exclusion criteria during the years in which data could be collected for this study.

#### 2.1.3 Clinical assessments

To ensure that patients' language and cognitive status enables full understanding of task requirements, we reviewed evaluations provided by their treating speech and occupational therapists. These evaluations were based on clinical tests performed at the Loewenstein Rehabilitation Medical Center, including: an aphasia screening test for Hebrew speaking patients—*the Israeli Loewenstein Aphasia Test* (*ILAT*; Schechter, 1965, Loewenstein Rehabilitation Medical Center, Raanana, Israel) which evaluates core language functions such as comprehension of words and sentences in oral and written language, naming, repetition, oral and written verbal expression; *SHEMESH* (Biran and Friedmann, 2004, 2005) which assesses object naming capacity (Hebrew) in aphasia; *PALPA*—*Psycholinguistic Assessments of Language Processing in Aphasia* (Kay et al., 1992; Hebrew version: Gil and Edelstein, 2001), which provides targeted assessment of specific language-processing components; *LOTCA*—*Loewenstein Occupational Therapy Cognitive Assessment* (Katz et al., 1989), which assesses basic cognitive functions (orientation, visual perception, spatial perception, praxis, visuo-motor organization, thinking) relevant for occupational therapy and cognitive rehabilitation.

#### 2.1.4 Participant demographics

In the right-hemisphere-damage group (RHD; *n* = 54), the mean age was 60.1 years (SD = 12.6), 19 patients were females, eight patients were left-handed, and the group's mean educational level was 13.4 years of formal schooling (SD = 3.2). Reflecting our inclusion criteria for subacute phase stroke, the mean time since stroke onset for this group was 8.7 weeks (SD = 4.6, range: 3–25 weeks), and the group comprised 36 (66.67%) ischemic and 13 (24.07%) hemorrhagic strokes, with three mixed (I/H; 5.56%), one AVM-H (1.85%), and one NA (1.85%). In the left-hemisphere-damage group (LHD; *n* = 39), the mean age was 59.2 years (SD = 13.8), 17 patients were females, two were left-handed, and the group's mean educational level was 12.7 years of formal schooling (SD = 2.8). Similarly, for the LHD group, the mean time since stroke onset was 8.3 weeks (SD = 4.2, range: 2.3–20 weeks), with 28 (71.8%) ischemic and 7 (17.9%) hemorrhagic strokes, with one mixed (I/H; 2.56%), one AVM-H (2.56%), and two CVST-H (5.13%). Supplementary Table S11 details each patient's demographic and clinical data.

Seventy-three healthy individuals of an age range of 21–84 years, with no history of neurological or psychiatric disorders, served as comparison group participants in return for payment. Their mean age was 57.9 years (SD = 16.9), and they had a mean educational level of 14.0 years (SD = 2.8); 33 were females; six were left-handed; handedness data was missing for eight participants. Ten age-matched healthy participants were selected for each stroke patient to construct individually matched comparison groups of comparable age. All participants provided informed consent to participate in the study, which was performed according to a protocol approved by the human subjects' research committees of the LRMC and WMC, following the ethical standards prescribed in the 1964 Declaration of Helsinki. The overall size of the healthy comparison group (*N* = 73) was thus dictated by the need to recruit a sufficient number of participants to enable the construction of these individually-matched comparison groups.

To assess for potential demographic confounding variables across groups, differences in continuous demographic variables such as age and education were examined using one-way ANOVA with a between-subjects factor of group (RHD, LHD, healthy participants), as it is appropriate for assessing differences in means across three or more independent groups. Group effect was not significant for both age, *F*_(2, 163)_ = 0.35, *p* **=** 0.70, and education, *F*_(2, 163)_ = 2.18, *p* **=** 0.12. To examine differences in proportions of categorical variables, specifically gender distribution across the groups (RHD, LHD, healthy participants), a Chi-square test of independence was performed, given its suitability for analyzing associations between two categorical variables. The M/F proportion did not differ between groups, χ^2^ (2, *N* = 166) = 1.37, *p* **=** 0.50. Thus, there was no difference between groups in terms of demographic variables.

### 2.2 Test procedures

The Family Pictures subtest, a recall memory test for details of visual scenes, was administered as part of the Wechsler Memory Scale-III (WMS-III; Wechsler, 1997) battery by trained personnel, according to standardized procedures of stimulus presentation, verbal instructions and a defined recall sequence. Adherence to the test standardized protocol minimizes variability in testing conditions and presentation order effects across participants. Patients were individually tested in a quiet, well-lit room at the rehabilitation center. Comparison group participants were tested at the Reichman University or at their homes. The WMS-III test battery was conducted in a single session lasting ~60–90 min, depending on the individual's personal pace. The Family Pictures subtest itself took ~10–15 min to complete, including the delayed recall phase. Short rest breaks were offered as needed between tests, based on participant preference or signs of fatigue.

Before beginning the task, participants were briefly familiarized with the characters featured in the task by viewing a picture that included all family members (mother, father, son, daughter, grandmother, grandfather, and dog). The experimenter explicitly labeled each member by their role. The picture lacked any contextual background or actions. This ensured recognition of the characters during their subsequent presentation as part of the different visual scenes. Following this familiarization, four distinct scene images (store, picnic, meal, garden), each depicting four of the family members engaged in different everyday activities, were presented sequentially, each for 10 s. Participants were instructed to remember as much detail as they could about each picture. Participants' instructions were as follows: “This is a picture of the characters you will see in the following four scenes. I will show you four scenes featuring these family members and their dog. Each scene will be shown for 10 s. Try to remember as much information as you can from each picture. Afterwards, I will ask you questions about the pictures you saw.” No practice trials were included, in accordance with the WMS-III standardized protocol.

### 2.3 Performance metric collection and timeframe

Immediately following the encoding phase, during which all scenes were presented, participants underwent an immediate recall test, sequentially for each scene, according to the presentation order in the encoding phase. During the recall tests participants were asked to recall for each scene in order, the actor identities (the specific characters that appeared in each scene), the locations [the precise spatial quadrant (1–4) on the page where each character was present], and the actions (the specific activity each character was performing) of the characters that appeared in each scene. A delayed recall phase was administered 30 min later, during which other subtests of the WMS-III were conducted, according to the standard procedure. This design, featuring a fixed delay with intervening tasks, inherently reduces recency effects and working memory influences, ensuring a robust assessment of delayed recall. Some patients (three LHD, five RHD) did not perform the delayed part of the test due to technical reasons. In those cases, only the immediate test phase was analyzed. As the Family Pictures subtest is a standardized clinical instrument, all participants viewed the same four scenes (store, picnic, meal, garden) in a fixed sequential order, as dictated by the WMS-III administration manual (Wechsler, 1997). Thus, counterbalancing or randomization was not applied.

### 2.4 Scoring method

Following WMS-III guidelines, participants received points for correctly recalled identities, locations, and actions. The maximum raw score was 16 for identities, 16 for locations, and 32 for actions, totaling 64 points. In addition to computing the total WMS-III score, we also analyzed those three subcomponents separately to create specific measures of Identity, Location, and Action scores. This approach allowed us to examine dissociable memory processes within visual episodic memory: the Identity score reflects the “who” (i.e., specific characters); the Location score represents the spatial “where” (i.e., precise quadrant recall); and the Action score indicates the “what happened” (i.e., specific event activity). This detailed scoring method thus provides a nuanced understanding of episodic visual scene memory.

### 2.5 Behavioral data analysis

**Figure 1:**
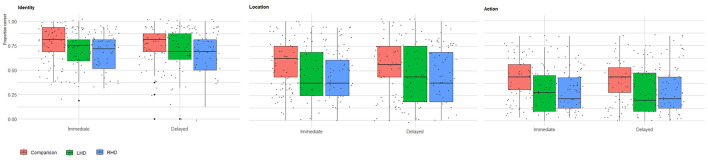
Immediate and delayed recall memory for memoranda identity, location, and action. Raw scores of healthy comparison group (*n* = 73), LHD (*n* = 39), and RHD (*n* = 54) participants for identity, location, and action memory in the immediate and delayed test phases. For each group, a boxplot with individual data points illustrates the distribution of that group's raw scores overall participants. The box marks the first and third quartiles, the continuous line marks the median, and the whiskers represent 1.5 times the interquartile range (i.e., the range between the first and third quartiles) above the third quartile and below the first quartile.

Memory performance was analyzed in RHD and LHD patients in comparison to matched healthy controls. This involved both group-based comparisons and individually matched patient analyses. For the latter, *z*-scores were computed for each patient relative to their individually matched group of 10 age-matched healthy controls, thereby normalizing performance relative to age-matched peers and accounting for age-related variability in memory performance. These *z*-scores then served as the dependent variable in subsequent lesion–behavior analyses. Prior to selecting the statistical model, normality of the raw Score variable was assessed using the Shapiro-Wilk tests, separately for each group. Measure type and testing phase. This analysis consistently indicated significant departures from normality on the majority of the measures (see Supplementary Table S1). Given this widespread non-normality and the proportional nature of the Score variable (bounded between 0 and 1), which was accordingly fit a Beta distribution, a Generalized Linear Mixed Model (GLMM) was used to examine differences in memory performance both between the RHD and LHD groups and between the patient groups and the comparison group, for each information type in immediate and delayed testing phases (Figure 1). We investigated the effects of group (RHD, LHD, controls), stimulus type (identity, location, action), and test phase (immediate, delayed), and their interactions, on participants' raw scores, while Subject ID was included as a random intercept to account for repeated measures within participants. Pairwise comparisons were adjusted using Bonferroni correction to control for Type I error rate inflation during multiple comparisons (see Figure 1). This analysis was conducted using R statistical software (R Core Team, version 2024.09.0). Statistical significance was set at *p* **<** 0.05. Normality tests and assessments of differences between groups in demographic variables (e.g., gender, age, and education), lesion extent and proportion of subjects affected in each brain region were performed using IBM SPSS Statistics (version 28.0.1.1).

### 2.6 Lesion analysis

Patients' brain damage was assessed using follow-up CT/MRI scans performed mostly during the subacute period, dating on average 41 days post-stroke onset (SD = 33), thus ensuring consistency in the stage of recovery in which the impact of brain damage was assessed. In some cases, where the boundaries of the damaged area were clearly defined in an earlier scan, lesion analysis was based on that initial scan (those latencies are included in the post-onset average time provided). Time between stroke onset and CT/MRI for each participant is detailed in Supplementary Table S11.

Lesion analyses were performed with the Analysis of Brain Lesions (ABLe) module implemented within MEDx software (Medical Numerics, Sterling, VA, USA). Lesions were manually outlined on the digitized CT/MRI scans using the MEDx software by a trained researcher on each axial slice where visible tissue damage was apparent. Each lesion was outlined using the pencil tool in 2D mode with locked dimensionality. Lesion boundaries delineation was independently confirmed and adjusted as required, slice by slice, by a physician experienced in neuroimaging data processing (author NS). This dual review process safeguarded quality control beyond automated methods of lesion delineation, and ensured that a systematic and standardized procedure of lesion analysis is undertaken across all participants.

ABLe characterizes brain lesions in MRI and CT scans of the adult human brain by first spatially normalizing the lesioned brain into Montreal Neurological Institute (MNI) space. This process standardizes the anatomical location and effective resolution of the images for subsequent lesion analysis. To ensure valid spatial normalization, registration accuracy to the MNI template was computed for each scan. Scans with registration accuracy below 90% were re-processed in an effort to improve alignment. Although not all scans reached this threshold after re-processing, they were retained in the analysis to preserve sample size, and registration accuracy was documented for transparency (see Supplementary Table S12). Final registration accuracy ranged from 89 to 95.6% (94.2 ± 1.2, 94.1 ± 1.1 in RHD and LHD subjects, respectively). Registration accuracy information from seven RHD patients and five LHD patients was not extant due to technical problems. Supplementary Table S12 shows the percent of damage for each brain region/structure in each patient. Subsequently, lesion volume was calculated, and affected brain regions were automatically identified in the normalized brain based on spatial overlap with the Automated Anatomical Labeling (AAL) atlas (Tzourio-Mazoyer et al., 2002) and the White Matter (WM) atlas (Lancaster et al., 2000; Mori et al., 2008). The ABLe software validation and accuracy metrics have been reported previously (Solomon et al., 2007), demonstrating reliable performance for automated lesion analysis in stroke patients. The specific procedures employed here follow those described in Haramati et al. (2008).

In addition, a comparison of lesion distribution was made between the RHD and LHD groups concerning (1) the proportion of subjects affected in each brain region/structure of the AAL and WM atlases (Lancaster et al., 2000; Solomon et al., 2007; Tzourio-Mazoyer et al., 2002; Mori et al., 2008). For these comparisons of proportions in each region, Fisher's exact test was utilized, as it is appropriate for analyzing associations between two categorical variables, especially when dealing with small sample size, for which the Chi-square test's assumptions might be violated and (2) the extent of damage (lesion volume) in each region, which often demonstrates a non-normal distribution, was compared using the Mann–Whitney test. This non-parametric test is suitable for assessing differences between two independent groups on a continuous variable without assuming normality. To control for the increased risk of Type I errors arising from the numerous comparisons across brain regions, the Holm-Bonferroni correction method was applied for multiple comparisons.

### 2.7 Voxel-based lesion-symptom mapping (VLSM)

Voxel-based lesion-symptom mapping (VLSM; Bates et al., 2003) was employed to identify brain structures where the existence of damage leads to memory deficits in specific components of a visual scene (identity, location, and action). Analysis of lesion impact at the voxel-level offers several key advantages over region-wise analyses, given the fact that the brain's regional nomenclature (e.g., that of cortical gyri and sulci or subcortical white-matter regions) is traditionally based largely on gross morphological appearance, often dis-regarding marked differences in functional neuroanatomy within regions. In contrast, VLSM provides a voxel-wise, data-driven approach that enables unbiased detection of lesion-deficit associations across the whole brain, without requiring prior hypotheses about specific anatomical regions. Moreover, by comparing behavioral scores between patients with and without damage to each voxel, VLSM yields high spatial resolution maps of brain–behavior relationships, allowing for more precise localization of functionally relevant areas (Bates et al., 2003). This makes it particularly well-suited for evaluating potentially dissociable neural substrates of complex memory components.

VLSM (Bates et al., 2003) analysis was conducted in the current study using the ABLe module within the MEDx software. For each voxel in standard MNI space, patients were classified as either lesioned or non-lesioned. Behavioral scores were compared between groups using the Mann–Whitney test, due to concerns about the normality assumption of traditional *t*-tests. Non-parametric alternatives are recommended for such cases and have been used extensively in past univariate lesion mapping studies (de Haan and Karnath, 2018). The output of this procedure is a map where each voxel is assigned a *z*-value, indicating the strength of the lesion-deficit association based on the Mann–Whitney *U*-test. To reduce noise and enhance reliability, we implemented several data filtering procedures: brain voxels were included in the VLSM analysis only if at least 10% of patients in the relevant group had damage to these voxels. This ensured sufficient data per voxel to support meaningful comparisons. Furthermore, voxels were considered significantly associated with behavioral deficit if they were part of a cluster of at least 20 contiguous “significant” voxels (for a similar method see Ben-Zvi et al., 2015), and additionally, they met either of the following criteria: (1) They surpassed a false discovery rate (FDR) corrected threshold of *p*FDR **<**0.05 (Benjamini and Hochberg, 1995; Genovese et al., 2002; Frenkel-Toledo et al., 2019); (2) In cases of reduced statistical power, regions were also identified using a more lenient criterion of uncorrected threshold of *p* **<** 0.005 (*z* > 2.6), consistent with prior lesion mapping studies (Wu et al., 2015; Schoch et al., 2006; Lo et al., 2010; Moon et al., 2016; Ben-Zvi Feldman et al., 2021, 2023). For each significant cluster, we report the maximum *z*-score and the MNI coordinates of the most superior, posterior, and leftward voxel with that value. Locations were labeled using the AAL atlas for gray matter and the White Matter Atlas (Lancaster et al., 2000; Solomon et al., 2007; Mori et al., 2008). To examine overlap across memory components, conjunction analysis was performed by overlaying VLSM maps for identity, location, and action across immediate and delayed recall phases using the uncorrected threshold (*p* **<** 0.005), enabling identification of shared anatomical correlates.

## 3 Results

### 3.1 Memory test performance

Memory performance varied across groups, stimulus type, and testing phases. Controls showed the highest overall performance, significantly outperforming the RHD group. Furthermore, performance was highest for identity memory, followed by location and action memory. Immediate testing phase performance was higher than performance on the delayed testing phase. Group average percent correct raw scores and average *z*-scores for identity, location, and action memory are presented in Table 1. A Generalized Linear Mixed Model (GLMM) was conducted to analyze the raw score variable, with group (Control, RHD, LHD), stimulus type (identity, location, action), and test phase (immediate, delayed) as fixed effects, and Subject ID as a random intercept. A significant main effect of group was observed (*p* **=** 0.009). Pairwise comparisons, using Bonferroni correction, indicated that the control group performed significantly better than the RHD group [control: predicted mean = 0.62, 95% CI (0.541, 0.700), RHD: predicted mean = 0.44, 95% CI (0.348, 0.541); *p* **=** 0.011]. No other significant differences were found between groups (all other Bonferroni-adjusted *ps* **>** 0.05). A significant main effect of stimulus type was also found (*p* **<** 0.001). Bonferroni-pairwise comparisons showed that score was highest for identity [predicted mean = 0.75, 95% CI (0.696, 0.790)], followed by location [predicted mean = 0.48, 95% CI (0.416, 0.536)], and lowest for action [predicted mean = 0.30, 95% CI (0.251, 0.354)]. All pairwise differences among action, identity, and location were statistically significant (*p*s **<** 0.0001). Additionally, a significant main effect was observed for test phase (*p* = 0.008), due to worse performance in the delayed test phase [predicted mean = 0.49, 95% CI [0.434, 0.553)] compared to the immediate test phase [predicted mean = 0.53, 95% CI (0.469, 0.588)]. The analysis revealed no statistically significant interaction effects among group, measure Type, and test phase (all *ps* **>** 0.1). Overall scores for each group (RHD, LHD, control), for each test phase (immediate, delayed), and stimulus type (identity, location, action) are shown in Table 2.

**Table 1:** Identity, location, and action memory on immediate and delayed test phases in RHD and LHD patient groups and the control group.

**Test phase**	**Group**	**Identity raw**	**Identity *Z*-score**	**Location raw**	**Location *Z*-score**	**Action raw**	**Action *Z*-score**	**Standard raw**	**Standard *Z*-score**
Immediate	RHD (*n* = 54)	0.68 (0.20)	−0.91 (1.68)	0.45 (0.26)	−0.76 (1.32)	0.31 (0.23)	−0.90 (1.63)	0.44 (0.22)	−0.93 (1.57)
	LHD (*n* = 39)	0.63 (0.28)	−0.76 (1.43)	0.41 (0.27)	−0.78 (1.20)	0.26 (0.22)	−1.14 (1.36)	0.39 (0.23)	−1.07 (1.37)
	Control (*n* = 73)	0.79 (0.15)	–	0.60 (0.23)	–	0.45 (0.18)	–	0.57 (0.17)	–
Delayed	RHD (*n* = 49)	0.61 (0.28)	−0.80 (1.97)	0.38 (0.31)	−0.50 (1.21)	0.27 (0.26)	−0.59 (1.43)	0.38 (0.26)	−0.69 (1.59)
	LHD (*n* = 36)	0.58 (0.32)	−0.74 (1.58)	0.40 (0.31)	−0.41 (1.27)	0.25 (0.23)	−0.76 (1.24)	0.37 (0.26)	−0.79 (1.50)
	Control (*n* = 73)	0.77 (0.19)	–	0.56 (0.26)	–	0.41 (0.20)	–	0.54 (0.19)	

Raw and Z-score matched identity, location, and action recall memory measures in the family pictures subtest of the WMS-III (see Methods section for explanation); SDs appear in parentheses. RHD/LHD = right/left hemisphere damage.

**Table 2:** Overall score of WMS III Family Pictures subtest for each group (RHD, LHD, healthy comparison group), test phase (immediate, delayed), and stimulus type (identity, location, action) factors.

**Factor**	**Group**	**Overall score**
Group	RHD	0.47 (0.22)
	LHD	0.47 (0.21)
	Healthy	0.60 (0.18)
Test phase	Immediate	0.54 (0.20)
	Delayed	0.52 (0.22)
Stimulus type	Identity	0.72 (0.19)
	Location	0.50 (0.26)
	Action	0.35 (0.22)

SDs appear in brackets. RHD/LHD = right/left hemisphere damage.

As a result of the heterogeneity in patients' age, which can affect memory performance, for the lesion-behavior analysis, we used the more individually focused age-matched *Z* scores as the behavioral measure for each patient (for a similar approach, see Ben-Zvi Feldman et al., 2021; Ben-Zvi et al., 2015).

### 3.2 Lesion analysis

Supplementary Table S12 shows the extent of damage to each region of the AAL and WM atlases for each patient. In the following brain regions, the proportion of subjects who had at least 5% of the region/structure damaged by the stroke was greater in the RHD than in the LHD patient group: superior, middle and polar regions of the temporal lobe; inferior frontal gyrus, Heschl gyrus, the Rolandic operculum, and putamen.

Additionally, the average extent of damage was significantly larger in the RHD compared to the LHD patient group in the following regions/structures: middle, superior and polar regions of the temporal lobe; orbital, inferior, and middle frontal gyri; supramarginal and angular gyri; insula, precentral and postcentral gyri, Heschl gyrus, the Rolandic operculum and white matter fibers of the superior longitudinal fasciculus, external capsule and superior corona radiata (detailed descriptive statistics are presented in Supplementary Table S13). The above group differences could affect VLSM results, therefore hemispheric differences in lesion effects of homologous regions should be interpreted with caution. It is important to note that there are differences in the opposite directions, showing average extent of damage or proportion of subjects who had at least 5% of the region/structure damaged which are greater in the LHD than in the RHD patient group, especially in temporo-occipital regions. However, those differences did not reach significance. Importantly, although those differences are not significant, they could lead to the finding of “significant” voxel clusters shown only in the LHD group.

**Figure 2:**
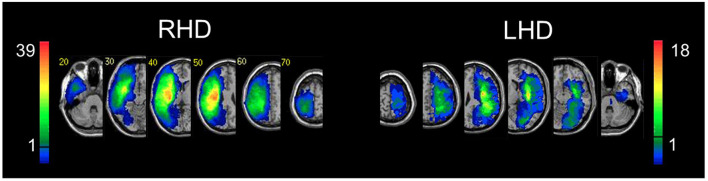
Lesion overlay. Representative normalized slices from 54 RHD patients **(left)** and 39 LHD patients **(right)**. A voxel was included in the VLBM analysis if damaged in at least 10% of the cohort (five and four patients in RHD and LHD groups, respectively). This threshold is denoted by the horizontal bar in the color code column representing the number of patients damaged in each region of the brain. Corner numbers indicate normalized slice numbers out of 90 total slices. The left side of the image corresponds to the right side of the brain (i.e., radiological convention).

Hemispheric volume loss ranged from 0.39 to 278 cm^3^ (*M* = 75 cm^3^, SD = 63 cm^3^) in the RHD group, and from 0.41 to 123.19 cm^3^ (*M* = 28 cm^3^, SD = 29 cm^3^) in the LHD group. Lesion size information from seven RHD patients and one LHD patient was not extant due to subsequent data loss (however, those data were available when the functional analyses reported below were conducted). Figure 2 shows the overlap of lesions in the RHD and LHD patient groups. As can be seen in this figure, most patients in both groups had lesions within the middle cerebral artery (MCA) territory, as is usually encountered in a cohort of stroke patients. Inferences from the current lesion analysis are confined to brain voxels affected in a sufficient number of subjects, as explained in the Methods section.

### 3.3 Voxel-based lesion-symptom mapping (VLSM)

VLSM analysis identified clusters of voxels in which damage was associated with impaired performance. As explained in the Methods section, patients' performance was expressed as *Z*-scores computed for each patient in reference to 10 age-matched healthy controls. VLSM analysis was done in 54 RHD patients in the immediate testing phase and 49 in the delayed testing phase. In the LHD group, VLSM analysis was done in 39 patients in the immediate testing phase and 36 patients in the delayed testing phase. The lists of brain regions containing clusters of 20 contiguous “significant” voxels or more are presented in Supplementary Tables S2–S9, which depict the VLSM results corresponding to the original WMS-III scoring method, and to the identity, location, and action recall memory components, for the RHD and LHD groups. In the VLSM analysis using the original WMS-III scoring method, memory performance is reflected by a composite measure of correct responses, without discrimination between identity, location, and action memory. In our approach, the raw score measure from the WMS-III was compared to 10 age-matched healthy controls for each patient to calculate *z*-scores which were entered into the VLSM analysis.

In the RHD group, the effects of damage on the composite subtest score (overall correct response rate) did not pass the FDR correction for both immediate and delayed testing phases. Results which passed only the lenient criterion of *z* = 2.6 (*p* < 0.005), occupying mainly lateral temporoparietal regions, are shown in Supplementary Table S2.

In the LHD group, VLSM analysis disclosed a markedly different pattern. First, the impact of damage on memory performance survived the FDR correction for multiple comparisons only at the delayed testing phase. Second, the voxel clusters in which the existence of damage was related to deficit in the overall recall capacity were concentrated in occipital (calcarine and inferior occipital regions) and temporo-occipital/MTL regions (lingual, parahippocampal, fusiform gyri, and the hippocampus; see Supplementary Table S3 for detailed results).

**Figure 3:**
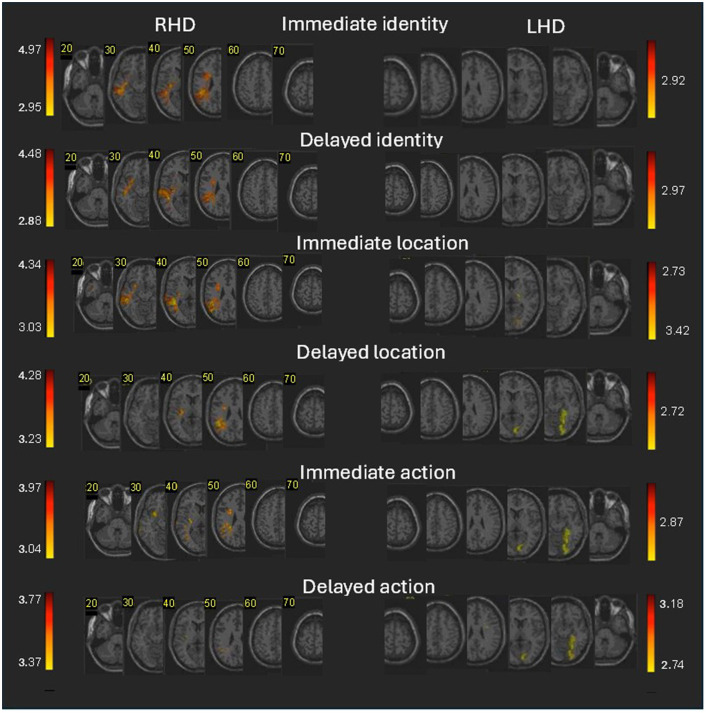
Voxel-based lesion-behavior mapping (VLSM) analysis in the RHD and LHD groups. Colored regions denote brain voxels in which the existence of damage significantly impacted the following measures of immediate and delayed memory for an object's identity, location, and action for the RHD **(left side)** and LHD **(right side)** patients' groups. The colored voxels passed threshold of *z* > 2.6 (*p* < 0.005). Voxels were analyzed only if they were damaged in at least 10% of the patients. Regions in red correspond to higher *z* scores. Corner numbers indicate normalized slice numbers out of 90 total slices. The left side of the image corresponds to the right side of the brain (i.e., radiological convention).

Turning to areas implicated by lesion effects in memory for the three components of scene memory, we found that memory for the immediate testing phase in RHD patients (Figure 3; Supplementary Tables S4–S9) was affected for the actor (person) identity and location memory by damage to sizable voxel clusters in the superior and middle temporal gyri, supramarginal and angular gyri of the lateral parietal cortex and the superior longitudinal fasciculus, together with smaller clusters in various locations which partially differ between different scene elements (see Supplementary Table S4 for detailed results). Those effects passed the FDR correction for multiple comparisons. Lesion effects for action memory showed similar patterns but passed only a lenient criterion of *z* = 2.6 (*p* **<** 0.005).

Lesion effects on Memory for the immediate testing phase in LHD patients for actor identity and location (Figure 3; Supplementary Tables S4, S5) did not pass the FDR correction. Memory for action (Supplementary Table S7; FDR corrected) was affected by damage to sizeable clusters in occipital (calcarine and inferior occipital gyrus) and temporo-occipital/MTL regions (lingual, fusiform and parahippocampal gyri).

The delayed testing phase in RHD patients was generally affected for the actor (person) identity and location memory by damage to smaller clusters of the same lateral temporo-parietal regions which affected those scene elements on the immediate testing phase (see Supplementary Tables S4–S6 for detailed results). The effects of damage to voxel clusters in this analysis passed the FDR correction for multiple comparisons. Lesion effects for action memory showed similar patterns but passed only a lenient criterion of *z* = 2.6 (*p* **<** 0.005).

The delayed testing phase in LHD patients was affected for location and action memory (FDR corrected) by damage to sizeable clusters in occipital regions (calcarine and inferior occipital gyrus) and temporo-occipital/MTL regions (lingual, fusiform and parahippocampal gyri). Lesion effects on actor identity (Figure 3; Supplementary Table S7) passed only a lenient criterion of *z* = 2.6 (*p* **<** 0.005) and showing small cluster of this area. Note that the anatomical pattern observed for the delayed testing phase in the LHD group is quite similar to the pattern previously described for the composite WMS-III score. Specifically, both sets of significant findings emerged at the delayed testing phase and yielded significant clusters in occipital and temporo-occipital/medial temporal regions.

### 3.4 VLSM conjunction analysis

**Figure 4:**
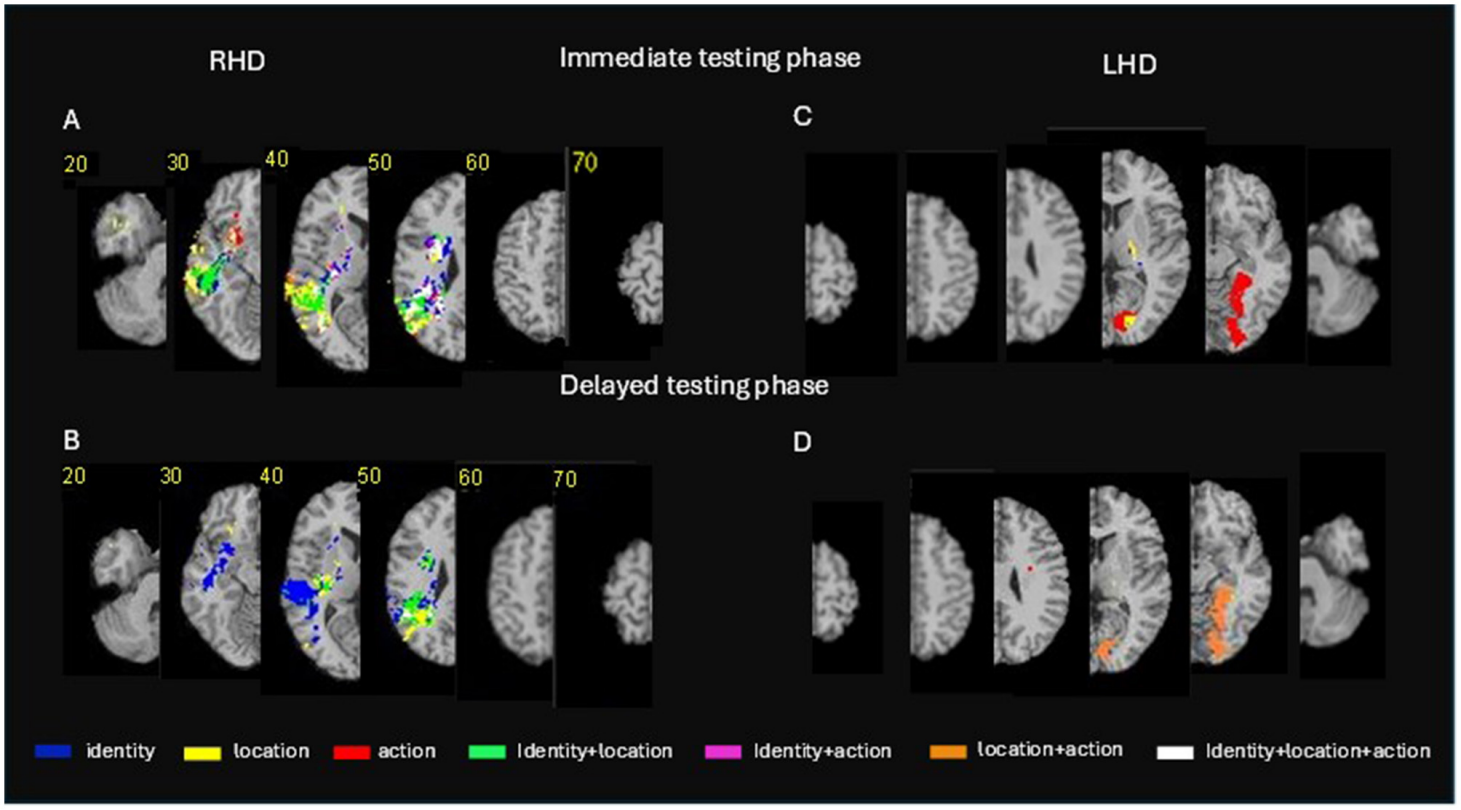
Voxel-based lesion-behavior mapping (VLSM) conjunction analysis. Conjunction VLSM analysis comparing: **(A)** (top left panel) identity vs. location vs. action memory measures on the immediate testing phase for the RHD group; **(B)** (lower left panel) the delayed testing phase for the RHD group; **(C)** (top right panel) the immediate testing phase for the LHD group; **(D)** (lower right panel) the delayed testing phase for the LHD group. The colored voxels passed the threshold of *z* > 2.6 (*p* < 0.005). Representative slices from VLSM maps were computed for each behavioral measure and overlaid on an MNI template brain. Colored pixels represent voxels in which the existence of damage exerted a significant impact on the tested behavior. Displays follow radiological convention, i.e., right hemisphere displayed on the left side. For a verbal description of the brain regions in which significant voxel clusters have been found in each conjunction analysis (see Supplementary Table S10).

While VLSM analysis of each individual task component is instructive in indicating areas implicated in these aspects of memory, a more constrained picture of the similarities and differences of the involvement of these areas in the task components is provided by conjunction analysis, in which all three sub-aspects are entered into the same analysis of performance at each of the stages (immediate and delayed). Figure 4 and Supplementary Table S10 show the anatomical structures where VLSM conjunction analysis revealed voxel clusters on the right and left hemispheres in which damage exerts a significantly specific impact on actor identity vs. location vs. action memory (or alternatively, an impact on all types) in the immediate and delayed testing phases. All anatomical comparisons are presented using a common threshold of *z* > 2.6 (*p* < 0.005) to accommodate measures that passed different threshold levels.

Conjunction VLSM analysis comparing actor identity vs. location vs. action memory measures on the immediate testing phase in the RHD group (Figure 4, top left panel; Supplementary Table S10A) reveals non-specific effects on all stimulus types by damage within the middle and superior temporal gyri, the angular and supramarginal gyri, the posterior and superior parts of the corona radiata and the posterior thalamic radiation. However, damage to other portions of the same anatomical regions were found to affect each of the three stimulus types in a specific manner. Damage to other cortical and subcortical regions of the right hemisphere was found to present mostly specific effects. Specificity for actor identity memory was found in the immediate testing phase for voxel clusters within the hippocampus, supramarginal gyrus, angular gyrus, superior and middle temporal gyri, putamen and several white matter tracts. Specificity for location memory was found in the immediate testing phase for large voxel clusters within the lateral temporal cortex, the angular gyrus and temporo-polar cortices. Specificity for action memory was found in the immediate testing phase for small voxel clusters within the lateral temporal cortices and the basal ganglia (see Supplementary Table S10 for fully detailed results). Damage to several voxel clusters, notably in temporo-parietal regions and some white matter tracts (detailed in Supplementary Table S10A) was found to affect two types of stimuli (i.e., actor identity plus location, actor identity plus action, or location plus action).

Conjunction VLSM analysis comparing actor identity vs. location vs. action memory measures on the delayed testing phase in the RHD group (Figure 4, top left panel; Supplementary Table S10A) revealed a non-specific effect (i.e., damage to voxel clusters affecting all three stimulus types) only in small cluster in the angular gyrus. Voxels' damage specifically affecting actor identity memory at the delayed testing phase was found within the superior, middle and inferior temporal cortex, the angular and supramarginal gyri, the hippocampus, putamen, and a few white matter tracts. Damage specifically affecting location memory at the delayed testing phase was found in the RHD group in large voxel clusters within the superior and middle temporal gyri, the angular and supramarginal gyri, the insula, putamen and several white matter tracts. There were no clusters with specific effect on action memory at the delayed testing phase in the RHD group. Damage to several, relatively small, voxel clusters in temporo-parietal regions and the superior longitudinal fasciculus was found to affect two types of stimuli (i.e., identity plus location, identity plus action or location plus action) in the delayed testing phase.

Conjunction VLSM analysis comparing actor identity vs. location vs. action memory measures on the immediate testing phase in the LHD group (Figure 4, top right panel; Supplementary Table S10B) did not reveal non-specific effects (i.e., effects on all the three stimulus types). A large cluster in the hippocampus affected identity and location memory.

Conjunction VLSM analysis comparing actor identity vs. location vs. action memory measures on the delayed testing phase in the LHD group (Figure 4, lower right panel; Supplementary Table S10B) revealed effects on both location and action memory for damage to large voxel clusters within the temporo-occipital cortex, in the lingual, fusiform, calcarine, parahippocampal, and inferior occipital gyri. Note that these are exactly the same voxels that affected action memory in what seems to be a specific manner in the immediate testing phase (Supplementary Tables S4–S9).

Given an appropriate statistical power, the involvement of these voxels most likely manifest a non-specific effect across multiple sub-measures also in the immediate testing phase. Note also that some of the effects in Supplementary Tables S4–S9) VLSM effects for identity, location and action memory) were obtained in voxel clusters that are exactly the same clusters that emerged in Supplementary Tables S2–S3, based on VLSM of the original composite scores (similarly, ascertained by cluster's *X*–*Y*–*Z* values and number of constituent voxels). Given that identity is scored in Supplementary Tables S4–S9 independently of other sub-measures, and location and action scoring depends on correct recall of identity, we may conclude that in all cases where a voxel cluster related to the composite measure (Supplementary Tables S2, S3) exactly overlaps a voxel cluster related to one of the sub-measures (Supplementary Tables S4–S9), the cluster relates to that sub-measure in a non-specific manner, i.e., by contributing to general episodic memory performance rather than to feature-specific processing. Note that this overlap happened mainly in temporo-occipital and medial temporal regions of the left hemisphere. However, as these regions were more frequently involved in our LHD group, this could lead to un-even statistical power in the analysis of lesion effects in the two hemispheric groups.

## 4 Discussion

The purpose of the present study was to investigate, using lesion-behavior mapping in patients after stroke, the shared and differential anatomical substrates of memory for identity, location and action of persons in a scene, which are key features of visual episodic memory. To this end, we have studied the impact of brain damage, at the voxel level, on performance of the *Family Pictures* subtest of the WMS-III. We first studied lesion effects on the original composite score, and then the shared (non-specific) and differential (specific) anatomical substrates of memory for the different scene elements (i.e., the identity, location and action of persons in these visual scenes). In the following, we consider the implications of our findings.

### 4.1 VLSM analysis of the family pictures composite score

Analysis of lesion effects on visual scene memory, as reflected in the original composite score of the *Family Pictures* test, disclosed a bi-hemispheric network with marked differences between the impact of lesion topography on performance following right hemisphere (RH) and left hemisphere (LH) stroke (Supplementary Tables S2, S3). In the RHD group, the network supporting visual scene memory was dominated by large voxel clusters in middle and superior temporal and inferior parietal regions of the lateral cortical mantle, with notable participation also of the superior longitudinal fasciculus (SLF) and other white-matter tracts. In contra-distinction, the network supporting visual scene memory in the LHD group was dominated by large voxel clusters in occipito-temporal and medial temporal lobe (MTL) regions, including the hippocampus. Given the nature of test materials—graphic illustrations of typical situations in family life, the involvement of primary visual (calcarine) and “ventral stream” components of the visual association cortex, as shown here in the LHD group, is expected (Xue, 2018). Involvement of MTL structures is also expected given the role of the hippocampus/MTL in declarative memory and the putative role of hippocampal-cortical connections in maintaining different types of memoranda as constituents of a single episode (for review see Squire and Dede, 2015; Henke, 2010). Involvement of temporo-parietal regions, as shown here in the RHD group, is also expected given the central role played by these regions in the core recollection network (Thakral et al., 2017). A word of caution concerning the above-mentioned hemispheric differences is required: lack of representation of temporo-parietal regions in the LH network could stem from recruitment bias, as LHD stroke patients with large infarctions in these regions are likely to be unable to participate due to severe aphasia. Indeed, comparative analysis disclosed greater temporo-parietal lesion extent and involvement rate in the RHD group (Supplementary Table S13A, B). As the original scoring method of the *Family Pictures* test is based on summation of scores obtained for correct recall of three sub-measures (actor identity, location, action), we deconstructed the composite score and analyzed the impact of lesion topography directly on the three sub-measures.

### 4.2 Remembering actor identity

Actor identity memory was most prominently affected, in the immediate testing phase, by damage to the right temporo-parietal cortex, the SLF, and the hippocampus. In the delayed testing phase, damage to part of these regions affected performance through involvement of much smaller voxel clusters (Supplementary Table S4). In the right temporo-parietal region, the majority of voxels in which the existence of damage was associated with impaired actor identity recall, were associated also with impaired recall of location or action or both. However, in the RH, unlike the LH, impaired memory of actor identity was associated also with damage to identity-specific voxels. This was found for aggregates of voxels in the right hippocampus, lateral temporo-parietal cortex, the middle occipital gyrus, and in some white matter tracts, more notably in the delayed testing phase (Supplementary Table S10).

### 4.3 Remembering the location of scene elements

Like actor identity memory, location memory was most prominently affected, in the immediate testing phase, by damage to the right temporo-parietal cortex and the SLF. Additional regions in the RH location memory network include the basal ganglia, insula, inferior temporal, and middle occipital areas as well as several white matter tracts. In the delayed testing phase, damage to part of these regions affected performance through involvement of much smaller voxel clusters (Supplementary Table S5). In addition, delayed location memory was affected by damage to the left occipito-temporal/MTL region including the hippocampus (Supplementary Table S8). Here, damage to the LH voxel clusters residing in the lingual, fusiform, parahippocampal, and inferior occipital gyri, affected location memory in a domain-general form, as evidenced by the fact that damage to exactly the same brain voxels also affected the recall of action details (Supplementary Tables S7–S9). The results of VLSM conjunction analysis in the LHD group (Supplementary Table S10) corroborate this conclusion by showing that the negative effect on location memory originated from damage to exactly the same voxels in which the existence of damage was associated with impaired memory for action details. However, damage to the left hippocampus affected delayed memory of the two sub-measures with partial voxel incongruence. Overall, the domain general nature of the LH temporo-occipital/MTL network subserving location memory stands in clear contrast to the RH network in which lesion effects showed a much more segregated impact on each sub measure. As can be seen in Supplementary Table S10, in the RH, temporo-parietal voxels showed a predominance of selective effects. Thus, the number of temporo-parietal voxels where damage affected location memory in a selective manner, outnumbered the number of voxels where the existence of damage affected location memory in a non-selective manner, i.e., in addition to actor identity and/or action memory. This predominance of selective effects suggests a degree of functional specialization within these right temporo-parietal voxels. It indicates that these areas are primarily engaged in processing the spatial aspects of an event, rather than serving as general nodes for processing all event components.

The superior and middle parts of the right temporal pole were implicated in the current VLSM analysis (Supplementary Tables S5, S10) as location-memory specific, both in the immediate and in the delayed testing phases. In accordance with this finding, the involvement of the right temporal pole in processing of spatial information was demonstrated in a case of a patient with focal atrophy located in the right temporal pole region, who managed to process and memorize visual information related to characteristics of presented stimuli, while failing in tasks requiring recall of objects' spatial position and in spatially based imagery operations (Luzzatti et al., 1998).

The current VLSM results identified a location-related voxel cluster in the right hippocampus, an area the integrity of which was also identified with actor identity memory. This is in accordance with the notion that the hippocampus is the binding site of object location and identity information, creating unified representations (Gaffan, 1998; Manns and Eichenbaum, 2006; Knierim et al., 2006; Diana et al., 2007). In the current paradigm, location memory is not an independent function, rather requiring identity information. It thus more greatly represents binding of location information with the identity information. Lesion studies have also shown that right hippocampal lesions cause deficits in various tasks of object-location and spatial memory (Kopelman et al., 1997; Pigott and Milner, 1993); (Smith and Milner, 1981, 1984; Holdstock et al., 2002, 2005; Crane and Milner, 2005; Nunn et al., 1999; Stepankova et al., 2004; van Asselen et al., 2009). Patients with hippocampal pathology due to perinatal anoxia (Vargha-Khadem et al., 1997) show impaired object-location memory. According to two reviews of the literature of lesion studies (Postma et al., 2008; Zimmermann and Eschen, 2017) and neuroimaging studies (Zimmermann and Eschen, 2017), the right hippocampus plays a critical role in object-location processing.

### 4.4 Remembering actions

As for actor identity and location, action memory was prominently affected by lesions in the right lateral temporoparietal regions and SLF in the immediate testing phase (not FDR corrected). However, in contrast with location memory, it was also prominently affected by lesions in left occipitotemporal areas both on the immediate and delayed testing phases. Some action-related clusters were action-specific, whereas others were implicated in performance on the other memory features.

In our VLSM results, the left insula was specifically related to action memory. This finding aligns with a functional imaging study by Paulus et al. (2005), where the insula was engaged during action selection and assessment. While deficits in action memory following damage to the insula could stem from impaired retrieval, they could also represent deficient encoding of perceptual or semantic aspects of gestures. This possibility is suggested by a lesion study (Binder et al., 2017) showing that damage to the left insula causes deficits in gesture comprehension, which may lead to an encoding failure rather than a failure of episodic recollection of actions.

### 4.5 Non-specific substrates

As noted, non-selective clusters on the right lateral temporo-parietal cortices—angular, superior temporal and middle temporal gyri (AG, STG, MTG)—were implicated by the VLSM analysis as important for all three memory functions assessed in this paradigm. Those regions are vital parts of the core recollection network (CRN), an episodic memory network that is specifically associated with recollection, according to functional imaging studies (Rugg and King, 2018; Vilberg and Rugg, 2012; Johnson and Rugg, 2007). The CRN, in accordance with the present findings, supports conscious access to representations of prior experiences, regardless of the content of the information recollected (Hayama et al., 2012; Johnson and Rugg, 2007; Rugg and Vilberg, 2013; Vilberg and Rugg, 2012). Therefore, voxel clusters supporting all memory features may perform essential memory functions, free of category or task segregation.

What kinds of memory processes are supported by the regions comprising the CRN? The lateral parietal cortex is the most studied among those regions, and hence, numerous suggestions regarding its memory functions were proposed, among which, attentional processes (e.g., Cabeza et al., 2008, 2011; Ciaramelli et al., 2008, 2010), expectation, and salience (Buchsbaum et al., 2011; O'Connor et al., 2010), retrieval buffering (Vilberg and Rugg, 2008, 2009a,b), or subjective report [Hower et al., 2014; Simons et al., 2010; reviewed by Levy (2012)]. It has also been suggested that parietal activity reflects stimulus-specific representations (Lee et al., 2017), memory precision (Richter et al., 2016), semantic representations (Binder et al., 2009; Price et al., 2015), and multimodal integration of features into a unified episodic representation (Shimamura, 2011). However, late-retrieval roles such as feature integration were not directly tested in the current study, as no condition assesses the binding of features together as opposed to single features' memory.

Furthermore, as noted, a large cluster of left occipitotemporal regions was associated with action and location memory in the delayed testing phase. This cluster constitutes part of the visual cortical processing stream associated with item identification, suggesting a perceptual processing role for those regions across all memory aspects. This is in accordance with the notion that episodic visual memory retrieval entails the reactivation of some of the same brain regions engaged when encoding the corresponding information, thus activating the visual cortices in the current task (Wheeler et al., 2000). However, since striate and extrastriate cortical lesions are often confounded with deficits in visual perception, it is difficult to know whether memory deficits after such lesions are due to damage of the storage representations or damage to the sites producing the percept that is to be stored.

Our finding that ventral temporo-occipital damage affects actors' identity memory along with memory for location and action data seems to challenge the dual-stream premises (Mishkin et al., 1983), which posit that these ventral regions preferentially process “what” information (identity of visual objects) whereas more dorsal occipito-parietal regions process “where” (location of visual objects) information. This finding also appears to contrast prior PET findings showing differentiation between identity and location memory, in relation to ventral and dorsal streams, respectively (Moscovitch et al., 1995; Köhler et al., 1998). However, this discrepancy could stem from differing analytical approaches in correlation (functional imaging) and causation (lesion effect) studies. The above-mentioned PET studies employed direct contrasts (e.g., identity > location) to show specificity in structure-function relationships. This could mask activations shared to a certain extent across tasks. In fact, the two streams revealed activations associated with both identity and location memory, when each task was contrasted with a separate baseline (Moscovitch et al., 1995). This latter approach uncovered broad regional involvement, more in accord with our lesion-based findings which assessed the necessity of regions for each task independently, thus revealing more shared neural substrates.

Furthermore, our use of the Family Pictures task, which reflects ecologically valid scene processing may require integrating multiple scene elements within complex contexts. This could engage a broader neural network than studies using isolated stimuli. The task's verbal report demands may also influence typical visuospatial processing patterns, potentially broadening observed activations.

Relatedly, in accordance with our findings, increasing evidence shows that dorsal and ventral streams carry both ‘what' and ‘where' information (albeit to different degrees). There is evidence that spatial information is also processed in the ventral stream (Lehky et al., 2008; Sereno and Lehky, 2011), while object shape information has been found in the dorsal stream (Sereno and Maunsell, 1998; Murata et al., 2000; Sereno and Amador, 2006; Konen and Kastner, 2008).

Additionally, our findings of neural underpinings of location memory seemingly contradict findings from Lugtmeijer et al. (2021), who identified using VLSM analysis the right frontal operculum as critical for episodic location memory (for criterion setting and not discrimination) while our results point to more distributed bilateral networks, specifically, right temporo-parietal involvement for immediate location memory and left occipito-temporal/MTL involvement for delayed location memory. This discrepancy may reflect differences in task demands, suggesting that ecologically valid scene processing, as in our study, engages broader and more complex neural systems than those recruited during simple spatial discrimination tasks of single objects.

We note that while the memoranda employed in the Family Pictures subtest are pictorial, verbal functions are required for proper report of recalled information. Prior studies have highlighted the task's reliance on language processing (e.g., Dulay et al., 2002; Chapin et al., 2009), suggesting that participants may rely on schematic knowledge or expectations when recalling actions or locations, rather than retrieving detailed perceptual information. As such, the observed performance could reflect memory for semantic representations of scene elements, or reasoning-based reconstructions, rather than direct recollection of specific visual or spatial details. Although we excluded patients exhibiting severe language impairments, it is possible that residual language impairments might have impacted on performance. This consideration introduces an important interpretive caveat when relating task performance to underlying neural substrates.

### 4.6 Limitations

While the results reported above indicate laterality effects in the involvement of different parts of the core recollection network (CRN) to scene memory, it should be noted that temporo-occipital/medial temporal regions within the territory of the posterior cerebral artery (PCA) were involved in just a small fraction of both LHD and RHD cohorts, with the latter group showing a smaller rate of involvement and in average a smaller extent of damage to these regions (Supplementary Table S13A, B). The non-significant but somewhat larger involvement in the LHD group could lead to demonstration of lesion effects in these regions in the LHD but not the RHD group due to lack of statistical power in the RHD cohort. Additionally, the lack of statistical power could lead also to type II errors in the VLSM analysis of lesion effects in the left-hemisphere. Specifically, there might be a lack of sufficient statistical power to detect lesion–behavior relationships in temporo-parietal peri-Sylvian regions of the left hemisphere due to the relatively small number of LHD participants. This stems from the exclusion of patients with significant aphasia, whose language impairments precluded comprehension of the task and valid participation. Thus, less involvement of temporo-parietal regions on the left (Supplementary Table S13A, B), could lead to demonstration of lesion effects in these regions in the RHD but not the LHD group. This limitation may affect the interpretation of our findings as it potentially exaggerates apparent hemispheric asymmetries.

Another limitation arising from our use of the *Family Pictures* subtest of the Wechsler Memory Scale (WMS-III) needs being mentioned. This subtest of the WMS does not provide optimal independent measures of location and action memory, as these scene elements are defined by the WMS as *meaningful* scene elements, in the sense of denoting the location and action of *properly recollected* key figures (the family members). Thus, this structure of the Family Pictures subtest limits the ability to assess identity, location, and action memory independently, which may have influenced the observed dissociations. As such, conclusions regarding functional specialization should be considered preliminary and warrant replication with more targeted and ecologically valid measures.

Furthermore, the identity, location, and action measures are not fully equivalent in the number of possible response choices. However, since the *Family Pictures* subtest uses ecological situations as a basis for its stimuli, participants presumably rely on their pre-existing schemas that create certain expectations for actions that can happen in the situation. The reliance on schemas makes the possible actions more limited and homogenous, and therefore the degrees of freedom for action memory are arguably of the same order of magnitude as the participant-identity and participant-location memory (albeit not having the same specific number of options).

Additionally, the analyses we present are not corrected for lesion volume, which has been suggested to bias the results of lesion-symptom mapping (DeMarco and Turkeltaub, 2018). However, it has also been recently argued that total lesion size is not a generally appropriate covariate control (Sperber, 2022), as it indiscriminately removes variance from analyses, thus making them conservative to the point of not detecting actual effects. Therefore, in the interests of enabling comparison with our prior studies regarding lateral temporo-parietal lesion effects (Ben-Zvi et al., 2015; Ben-Zvi Feldman et al., 2021, 2023), we similarly conducted our analyses here without lesion volume corrections. While aligning with our previous work, this approach means our specific findings should be interpreted with awareness of these potential biases in lesion-symptom mapping.

Another possible limitation of the current study is that we used false discovery rate (FDR) corrections to estimate statistically significant patterns of lesions effects. In recent analyses (e.g., Mirman et al., 2018), it has been suggested that FDR correction might underestimate the rate of false positives, especially with a smaller sample size. To compensate for these concerns, we employed two methods of reducing false positives yielded by multiple comparisons: only relating to lesion effects for clusters of at least 20 contiguous voxels, and only including in analyses voxels damaged in at least 10% of the group subjects. Both these requirements considerably decrease the multiplicity of comparisons and therefore reduce the possibility of false positives. We also thought it most appropriate to use the same methods employed in our prior studies of temporo-parietal lesion effects on episodic memory (Ben-Zvi et al., 2015; Ben-Zvi Feldman et al., 2021, 2023), to enable direct comparison of the present results with those findings.

Another limitation of the study relates to the “mass-univariate” approach used here for analysis of lesion effects at the voxel level. This approach seems to implicitly assume that damage to each voxel affects task performance independently of what happens in other brain voxels. Such a conclusion is of-course wrong, given the reliance of proper brain functioning on the coordinated activity of networks of neurons. Also, in standard VLSM, damage to functionally irrelevant voxels may be tightly associated with damage to critical voxels, due to reliance on supply coming from the same branch of the vascular tree. This phenomenon makes it difficult to distinguish voxel clusters relevant to the function in question from irrelevant voxel clusters, thus it may lead to erroneous conclusions about the anatomical basis of deficits. It was noted, e.g., by (Mah et al. 2014), that the mass univariate approach in VLSM can lead to significant and consistent spatial mislocalization of the relevant lesion topography, where the direction of errors is not random but instead follows the architecture of the neurovascular system (Mah et al., 2014).

Another caveat regarding the research is that brain scans were conducted at time points determined by clinical considerations, and patient participation timing was constrained by rehabilitation-treatment needs.

Although the mean time between scans used in the VLSM and memory testing was in the order of 3 weeks, individual time lags varied greatly across the patient population (range: −23 to 40 weeks, SD: 8 weeks, i.e., in one extreme case memory testing took place 23 weeks before the follow-up CT eventually used for lesion analysis, while in the other extreme case it was conducted 40 weeks after the time of imaging). This variability in timing may introduce noise into our lesion-behavior correlations, through the effect of dynamic processes of neural recovery and functional re-mapping, thus potentially impacting the precision of our functional localization conclusions.

## 5 Future directions

Future studies should aim to improve ecological validity by employing tasks that dissociate memory for actors' identity, location, and action within more naturalistic contexts, enabling assessment also of memory of the temporal order of events, as in episodic memory of real-life situations (for example by using video clips of daily events rather than static pictures). Expanding to larger patient samples, with more participation of stroke patients damaged in the less frequently affected territories of the posterior and anterior cerebral arteries, will enhance the generalizability of lesion-behavior relationships. Finally, leveraging more advanced analytic approaches, such as network-based lesion mapping could yield deeper insights into the distributed neural systems supporting complex episodic memory processes.

## 6 Conclusions

The current study identifies a distributed set of cortical and sub-cortical regions supporting visual memory for persons, actions, and locations portrayed in a narrative scene. Those regions include extrastriate and other cortical areas associated with visual perception and information processing. Our finding of differential processing for each visual element are consistent with findings from functional imaging and lesion studies. Additionally, non-specific processing of those features was also demonstrated in other clusters in left temporo-occipital and right lateral temporo-parietal networks, presumably related to generic visual episodic memory processes. In a diagnostic context, this may lead to better understanding of the subjective memory problems many stroke patients experience in daily life.

## Data Availability

The raw data supporting the conclusions of this article will be made available by the authors, without undue reservation.

## Supplementary material

Supplementary Figure S1 Individual lesion data. Normalized lesion data. Each patient's lesion is marked on an array of 11 standard atlas templates (Damasio and Damasio, 1989), with lesions shown in white and overlaid on Brodmann areas, which are color-coded to highlight different regions. Displays follow neurological conventions, i.e., right-sided damage displayed on the left and left-sided on the right side. In the case of a minimal lesion, the MEDx system does not depict the lesion in the restricted set of standard templates used to present the structural damage. Data required for the preparation of this figure were not available for seven RHD and nine LHD patients.

Supplementary Table S1 Normality of Identity, Location, and Action memory performance was evaluated using Shapiro-Wilk tests for RHD, LHD, and Control groups at Immediate and Delayed phases. Results include the W statistic, df, and *p*-value; data were considered not normally distributed if *p* < 0.05.

Supplementary Table S2 VLSM analysis, RHD group: Voxel clusters where damage significantly affected the WMS-III measure of immediate and delayed memory for family pictures.

Supplementary Table S3 VLSM analysis, LHD group: Voxel clusters where damage significantly affected the WMS-IIImeasure of immediate and delayed memory for family pictures.

Supplementary Table S4 VLSM analysis, RHD group: Voxel clusters where damage significantly affected identity memory.

Supplementary Table S5 VLSM analysis, RHD group: Voxel clusters where damage significantly affected location memory.

Supplementary Table S6 VLSM analysis, RHD group: Voxel clusters where damage significantly affected action memory.

Supplementary Table S7 VLSM analysis, LHD group: Voxel clusters where damage significantly affected identity memory.

Supplementary Table S8 VLSM analysis, LHD group: Voxel clusters where damage significantly affected location memory.

Supplementary Table S9 VLSM analysis, LHD group: Voxel clusters where damage significantly affected action memory.

Supplementary Table S10 VLSM conjunction analysis in the Immediate and Delayed testing phases for the RHD and LHD groups.

Supplementary Table S11 Demographic and clinical characteristics of individual patients.

Supplementary Table S12 Extent of damage (%) in brain regions defined by the AAL and WM atlases for individual RHD and LHD patients.

Supplementary Table S13 **(A)** Brain regions in which the percent of subjects who had at least 5% of the region damaged by the stroke was significantly larger in the RHD group compared to the LHD group. **(B)** Brain regions in which the average extent of damage (%) was significantly larger in the RHD group compared to the LHD group.
