# Supplementary figures and images for: Brain substrates of visual scene memory: a lesion-behavior mapping study
Source: Front Hum Neurosci. 2025 Sep 1;19:1606051. doi: 10.3389/fnhum.2025.1606051 (PMC12434039; doi:10.3389/fnhum.2025.1606051)

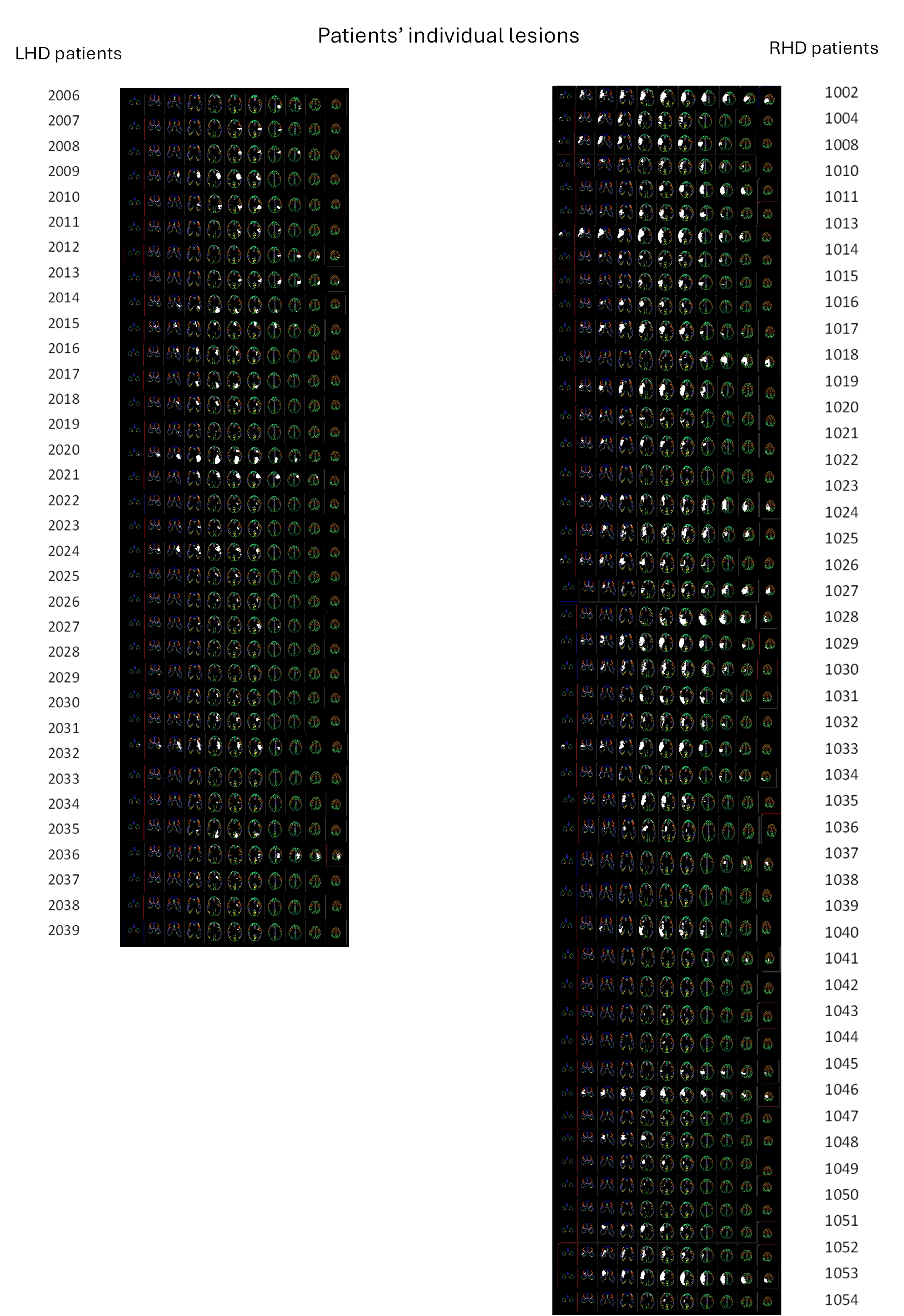

Supplement: Supplementary Figure S1 — Individual lesion data. Normalized lesion data. Each patient's lesion is marked on an array of 11 standard atlas templates (Damasio and Damasio, 1989), with lesions shown in white and overlaid on Brodmann areas, which are color-coded to highlight different regions. Displays follow neurological conventions, i.e., right-sided damage displayed on the left and left-sided on the right side. In the case of a minimal lesion, the MEDx system does not depict the lesion in the restricted set of standard templates used to present the structural damage. Data required for the preparation of this figure were not available for seven RHD and nine LHD patients. [file Image_1.jpeg]
