# Supplementary material for: Brain substrates of visual scene memory: a lesion-behavior mapping study
Source: Front Hum Neurosci. 2025 Sep 1;19:1606051. doi: 10.3389/fnhum.2025.1606051 (PMC12434039; doi:10.3389/fnhum.2025.1606051)
Supplement: Supplementary Table S1 — Normality of Identity, Location, and Action memory performance was evaluated using Shapiro-Wilk tests for RHD, LHD, and Control groups at Immediate and Delayed phases. Results include the W statistic, df, and p-value; data were considered not normally distributed if p < 0.05. [file Table_1.docx]

**Supplementary Materials**

**Table S1.** Normality of Identity, Location, and Action memory performance was evaluated using Shapiro-Wilk tests for RHD, LHD, and Control groups at Immediate and Delayed phases. Results include the *W* statistic, df, and *p*-value; data were considered not normally distributed if *p* < 0.05.

**Table S2.** VLSM analysis, RHD group: Voxel clusters where damage significantly affected the WMS-III measure of immediate and delayed memory for family pictures.

**Table S3.** VLSM analysis, LHD group: Voxel clusters where damage significantly affected the WMS-IIImeasure of immediate and delayed memory for family pictures.

**Table S4.** VLSM analysis, RHD group: Voxel clusters where damage significantly affected identity memory.

**Table S5.** VLSM analysis, RHD group: Voxel clusters where damage significantly affected location memory.

**Table S6.** VLSM analysis, RHD group: Voxel clusters where damage significantly affected action memory.

**Table S7.** VLSM analysis, LHD group: Voxel clusters where damage significantly affected identity memory.

**Table S8.** VLSM analysis, LHD group: Voxel clusters where damage significantly affected location memory.

**Table S9.** VLSM analysis, LHD group: Voxel clusters where damage significantly affected action memory.

**Table S10.** VLSM conjunction analysis in the Immediate and Delayed testing phases for the RHD and LHD groups.

**Table S11.** Demographic and clinical characteristics of individual patients.

**Table S12.** Extent of damage (%) in brain regions defined by the AAL and WM atlases for individual RHD and LHD patients.

**Table S13:**

A. Brain regions in which the percent of subjects who had at least 5% of the region damaged by the stroke was significantly larger in the RHD group compared to the LHD group.

B. Brain regions in which the average extent of damage (%) was significantly larger in the RHD group compared to the LHD group.

**Abbreviations**

RHD = right hemisphere damage

LHD = left hemisphere damage

g = gyrus

BCC = body of the corpus callosum

CGC = cingulum (in the cingulate gyrus)

CGH = cingulum (in the hippocampal region)

CP = cerebral peduncle

CR_S/A/P_ = superior/anterior/posterior part of the corona radiata

CST = corticospinal tract

EC = external capsule

FO_S/I_ = superior/inferior portion of the fronto-occipital fasciculus

FX/ST = fornix and stria terminalis

GCC = genu of the corpus callosum.

IC_AL/PL/RL_ = anterior/posterior/retrolenticular limb of the internal capsule

ICP = inferior cerebellar peduncle

IFG_TRI/ORB/OP_ = triangular/orbital/opercular parts of the inferior frontal gyrus

MCP = inferior/superior/middle cerebellar peduncle

ML = medial lemniscus

PCT = pontine crossing tract

SLF = superior longitudinal fasciculus

Rolandic op. = Rolandic operculum

SCC = splenium of the corpus callosum

SCP = superior cerebellar peduncle

SS = sagittal stratum

TAP = tapetum

TP_S/M_ = superior/middle parts of the temporal pole

TR_P_ = posterior thalamic radiation

UNC = uncinate fasciculus

**Table S1 Shapiro-Wilk Tests of Normality**

| Test phase | Variable | Group | Statistic (*W*) | df | Sig. (*p*) | Normality Conclusion |
| --- | --- | --- | --- | --- | --- | --- |
| Immediate | Identity | RHD | 0.913 | 49 | 0.002 | Not Normal |
|  |  | LHD | 0.933 | 36 | 0.03 | Not Normal |
|  |  | Control | 0.929 | 73 | 0 | Not Normal |
|  | Location | RHD | 0.942 | 49 | 0.018 | Not Normal |
|  |  | LHD | 0.958 | 36 | 0.185 | Normal |
|  |  | Control | 0.971 | 73 | 0.09 | Normal |
|  | Action | RHD | 0.88 | 49 | 0 | Not Normal |
|  |  | LHD | 0.934 | 36 | 0.033 | Not Normal |
|  |  | Control | 0.985 | 73 | 0.554 | Normal |
| Delayed | Identity | RHD | 0.959 | 49 | 0.084 | Normal |
|  |  | LHD | 0.92 | 36 | 0.013 | Not Normal |
|  |  | Control | 0.871 | 73 | 0 | Not Normal |
|  | Location | RHD | 0.925 | 49 | 0.004 | Not Normal |
|  |  | LHD | 0.938 | 36 | 0.045 | Not Normal |
|  |  | Control | 0.965 | 73 | 0.042 | Not Normal |
|  | Action | RHD | 0.882 | 49 | 0 | Not Normal |
|  |  | LHD | 0.914 | 36 | 0.008 | Not Normal |
|  |  | Control | 0.981 | 73 | 0.355 | Normal |

**Table S2. VLSM analysis, RHD group: Voxel clusters where damage significantly affected the WMS-III measure of immediate and delayed memory for family pictures.**

| Structure | Test phase | Z-value | X | Y | Z | Cohen’s r | Voxels | % Area |
| --- | --- | --- | --- | --- | --- | --- | --- | --- |
| Middle Temporal g | Immediate | 3.61 | 40 | -54 | 18 | 0.49 | 534 | 12.11 |
|  | Delayed | 3.03 | 42 | -52 | 18 | 0.43 | 22 | 0.5 |
| Superior Temporal g | Immediate | 3.74 | 48 | -42 | 18 | 0.51 | 272 | 8.66 |
|  | Delayed | 3.54 | 42 | -32 | 18 | 0.51 | 116 | 3.69 |
|  |  | 3.17 | 66 | -10 | -4 | 0.45 | 45 | 1.43 |
| Angular g | Immediate | 3.88 | 44 | -46 | 22 | 0.53 | 190 | 10.84 |
|  | Delayed | 3.76 | 42 | -50 | 28 | 0.54 | 94 | 5.37 |
| SLF | Immediate | 3.95 | 36 | -48 | 18 | 0.54 | 157 | 19.03 |
|  | Delayed | 3.36 | 36 | -48 | 18 | 0.48 | 78 | 9.45 |
| Putamen | Immediate | 4.28 | 32 | -8 | -8 | 0.58 | 125 | 11.75 |
|  | Delayed | 3.83 | 32 | -8 | -8 | 0.55 | 122 | 11.47 |
| EC | Immediate | 3.66 | 32 | -12 | 8 | 0.50 | 103 | 22.1 |
|  | Delayed | 3.74 | 32 | -12 | 8 | 0.53 | 94 | 20.17 |
| TR_P_ | Immediate | 4.25 | 34 | -46 | 18 | 0.58 | 93 | 19.1 |
|  | Delayed | - | - | - | - | - | - | - |
| Middle Occipital g | Immediate | 3.19 | 46 | -64 | 28 | 0.43 | 75 | 3.57 |
|  | Delayed | - | - | - | - | - | - | - |
| CR_P_ | Immediate | 3.47 | 26 | -48 | 26 | 0.47 | 66 | 14.6 |
|  | Delayed | 3.25 | 26 | -48 | 26 | 0.46 | 35 | 7.74 |
| IC_PL_ | Immediate | 3.8 | 28 | -18 | 8 | 0.52 | 57 | 11.38 |
|  | Delayed | 3.73 | 26 | -10 | 12 | 0.53 | 52 | 10.38 |
| FO_I_ | Immediate | 4.28 | 32 | -8 | -8 | 0.58 | 57 | 21.67 |
|  | Delayed | 3.83 | 32 | -8 | -8 | 0.55 | 40 | 15.21 |
| Insula | Immediate | 3.69 | 34 | -16 | 14 | 0.50 | 53 | 2.99 |
|  | Delayed | 3.97 | 34 | -16 | 14 | 0.57 | 56 | 3.16 |
| Pallidum | Immediate | 4.13 | 30 | -8 | -4 | 0.56 | 51 | 18.21 |
|  |  | 4.06 | 28 | -6 | -6 | 0.55 | 63 | 22.5 |
| SupraMarginal g | Immediate | 3.33 | 52 | -46 | 26 | 0.45 | 43 | 2.18 |
|  | Delayed | 3.3 | 46 | -36 | 22 | 0.47 | 22 | 1.11 |
| IC_RL_ | Immediate | 3.9 | 28 | -20 | 8 | 0.53 | 42 | 13.29 |
|  | Delayed | - | - | - | - | - | - | - |
| Hippocampus | Immediate | 3.1 | 38 | -8 | -14 | 0.42 | 31 | 3.28 |
|  | Delayed | - | - | - | - | - | - | - |
| CR_S_ | Immediate | 3.65 | 26 | -10 | 22 | 0.50 | 27 | 2.93 |
|  | Delayed | 3.53 | 26 | -10 | 22 | 0.50 | 60 | 6.52 |

Brain regions defined by the AAL and WM atlases with ‘significant’ clusters of ≥ 20 voxels. Presented results correspond to a z score of 2.6, )*p* < .005). Regions are sorted by the number of ‘significant’ voxels. X, Y, Z coordinates indicate the voxel that is most superior, posterior, and left in its location within the cluster.

**Table S3. VLSM analysis, LHD group: Voxel clusters where damage significantly affected the WMS-III measure of immediate and delayed memory for family pictures.**

| Structure | Test phase | Z-value | X | Y | Z | Cohen’s r | Voxels | % Area |
| --- | --- | --- | --- | --- | --- | --- | --- | --- |
| Lingual g | Immediate | - | - | - | - | - | - | - |
|  | Delayed | 2.84 | -26 | -86 | -18 | 0.47 | 729 | 34.8 |
| Calcarine | Immediate | - | - | - | - | - | - | - |
|  | Delayed | 2.84 | -8 | -86 | -6 | 0.47 | 333 | 14.75 |
| Fusiform g | Immediate | - | - | - | - | - | - | - |
|  | Delayed | 2.84 | -22 | -78 | -18 | 0.47 | 205 | 8.87 |
| ParaHippocampal g | Immediate | - | - | - | - | - | - | - |
|  | Delayed | 2.84 | -24 | -28 | -14 | 0.47 | 109 | 11.15 |
| Inferior Occipital g | Immediate | - | - | - | - | - | - | - |
|  | Delayed | 2.84 | -26 | -80 | -12 | 0.47 | 92 | 9.78 |
| Hippocampus | Immediate | - | - | - | - | - | - | - |
|  | Delayed | 2.84 | -28 | -28 | -12 | 0.47 | 27 | 2.9 |
| IC_PL_ | Immediate | 2.98 | -22 | -14 | 14 | 0.48 | 21 | 4.4 |
|  | Delayed | - | - | - | - | - | - | - |

Brain regions defined by the AAL and WM atlases with ‘significant’ clusters of ≥ 20 voxels. Presented results correspond to a z score of 2.6, )*p* < .005). The analysis for the delayed memory measure has also passed FDR correction for multiple comparisons (corresponding to a z score of 2.58). Regions are sorted by the number of ‘significant’ voxels. X, Y, Z coordinates indicate the voxel that is most superior, posterior, and left in its location within the cluster.

**Table S4. VLSM analysis, RHD group: Voxel clusters where damage significantly affected identity memory.**

| Structure | Test phase | Z-value | X | Y | Z | Cohen’s r | Voxels | % Area |  |
| --- | --- | --- | --- | --- | --- | --- | --- | --- | --- |
| Middle Temporal g | Immediate | 4.42 | 48 | -44 | 18 | 0.60 | 994 | 22.54 | |
|  | Delayed | 3.76 | 50 | -44 | 18 | 0.54 | 367 | 8.32 | |
| Superior Temporal g | Immediate | 4.97 | 48 | -42 | 18 | 0.68 | 588 | 18.72 | |
|  | Delayed | 4.48 | 52 | -42 | 18 | 0.64 | 1539 | 49 | |
| Angular g | Immediate | 3.95 | 44 | -46 | 22 | 0.54 | 311 | 17.75 | |
|  | Delayed | 3.85 | 48 | -44 | 22 | 0.55 | 219 | 12.5 | |
| SLF | Immediate | 4.55 | 38 | -40 | 24 | 0.62 | 287 | 34.79 | |
|  |  | 3.39 | 34 | -2 | 28 | 0.46 | 20 | 2.42 | |
|  | Delayed | 4.16 | 38 | -40 | 24 | 0.59 | 187 | 22.67 | |
| SupraMarginal g | Immediate | 4.09 | 46 | -42 | 28 | 0.56 | 142 | 7.19 | |
|  | Delayed | 3.93 | 46 | -36 | 22 | 0.56 | 120 | 6.08 | |
| CR_S_ | Immediate | 3.87 | 22 | -2 | 32 | 0.53 | 139 | 15.11 | |
|  |  | 3.79 | 26 | -10 | 20 | 0.52 | 61 | 6.63 | |
|  | Delayed | 3.56 | 22 | -6 | 30 | 0.51 | 61 | 6.63 | |
| SS | Immediate | 4.14 | 42 | -18 | -16 | 0.56 | 133 | 46.5 | |
|  | Delayed | 3.37 | 42 | -18 | -12 | 0.48 | 48 | 16.78 | |
| CR_P_ | Immediate | 3.8 | 22 | -22 | 30 | 0.52 | 121 | 26.77 | |
|  | Delayed | 3.6 | 30 | -42 | 20 | 0.51 | 97 | 21.46 | |
| IC_RL_ | Immediate | 4.06 | 28 | -22 | 14 | 0.55 | 120 | 37.97 | |
|  | Delayed | 3.86 | 30 | -20 | 0 | 0.55 | 89 | 28.16 | |
| IC_PL_ | Immediate | 3.85 | 28 | -16 | 8 | 0.52 | 110 | 21.96 | |
|  | Delayed | 3.42 | 22 | -20 | 12 | 0.49 | 48 | 9.58 | |
| TR_P_ | Immediate | 4.28 | 34 | -42 | 18 | 0.58 | 93 | 19.1 | |
|  | Delayed | 4 | 34 | -40 | 16 | 0.57 | 44 | 9.03 | |
| Putamen | Immediate | 3.64 | 32 | -8 | -4 | 0.50 | 86 | 8.08 | |
|  | Delayed | 3.64 | 34 | -6 | -8 | 0.52 | 141 | 13.25 | |
| EC | Immediate | 3.75 | 32 | -20 | 6 | 0.51 | 80 | 17.17 | |
|  | Delayed | 3.66 | 32 | -6 | 14 | 0.52 | 103 | 22.1 | |
| Hippocampus | Immediate | 4.14 | 42 | -18 | -16 | 0.56 | 74 | 7.82 | |
|  | Delayed | 3.37 | 42 | -14 | -14 | 0.48 | 32 | 3.38 | |
| Insula | Immediate | 3.28 | 32 | -22 | 12 | 0.45 | 69 | 3.9 | |
|  | Delayed | 3.59 | 34 | -12 | 8 | 0.51 | 93 | 5.25 | |
| Thalamus | Immediate | 3.75 | 18 | -12 | 10 | 0.51 | 57 | 5.39 | |
|  | Delayed | - | - | - | - | - | - | - | |
| Middle Occipital g | Immediate | 3.03 | 48 | -68 | 28 | 0.41 | 56 | 2.67 | |
|  | Delayed | 2.88 | 38 | -62 | 32 | 0.41 | 26 | 1.24 | |
| IFG_OP_ | Immediate | 3.02 | 42 | 6 | 22 | 0.41 | 38 | 2.72 | |
|  | Delayed | - | - | - | - | - | - | - | |
| FO_I_ | Immediate | 4.03 | 34 | -8 | -12 | 0.55 | 38 | 14.45 | |
|  | Delayed | 3.64 | 34 | -6 | -12 | 0.52 | 76 | 28.9 | |
| Inferior Temporal g | Immediate | 3.76 | 54 | -28 | -16 | 0.51 | 29 | 0.82 | |
|  | Delayed | 3.23 | 56 | -26 | -18 | 0.46 | 40 | 1.12 | |
| Inferior Parietal lobule | Immediate | 2.95 | 46 | -42 | 38 | 0.40 | 23 | 1.71 | |
|  | Delayed | - | - | - | - | - | - | - | |
| Heschl g | Immediate | 3.83 | 34 | -28 | 14 | 0.52 | 23 | 9.24 | |
|  | Delayed | 3.66 | 36 | -28 | 16 | 0.52 | 67 | 26.91 | |
| Precentral g | Immediate | 3.2 | 34 | 4 | 30 | 0.44 | 23 | 0.68 | |
|  | Delayed | - | - | - | - | - | - | - | |
| FX/ST | Immediate | 3.46 | 30 | -18 | -8 | 0.47 | 23 | 16.79 | |
|  | Delayed | - | - | - | - | - | - | - | |
| BCC | Immediate | 3.54 | 18 | -2 | 32 | 0.48 | 21 | 1.22 | |
|  | Delayed | - | - | - | - | - | - | - | |
| IC_AL_ | Immediate | - | - | - | - | - | - | - | |
|  | Delayed | 3.25 | 16 | 16 | 0 | 0.46 | 21 | 5.16 | |

Brain regions defined by the AAL and WM atlases with ‘significant’ clusters of ≥ 20 voxels. Results presented correspond to a z score of 2.6, )*p* < .005(. Analyses of those measures were also passed FDR correction for multiple comparisons (corresponding to a z score of 2.47 and 2.55 for the immediate and the delayed tests, respectively). those results were also passed the FDR correction for multiple comparisons. Regions are sorted by the number of ‘significant’ voxels. X, Y, Z coordinates indicate the voxel that is most superior, posterior, and left in its location within the cluster.

**Table S5. VLSM analysis, RHD group: Voxel clusters where damage significantly affected location memory.**

| Structure | Test phase | Z-value | X | Y | Z | Cohen’s r | Voxels | % Area |
| --- | --- | --- | --- | --- | --- | --- | --- | --- |
| Middle Temporal g | Immediate | 3.94 | 44 | -44 | 18 | 0.54 | 1598 | 36.24 |
|  | Delayed | 3.63 | 44 | -44 | 18 | 0.52 | 72 | 1.63 |
| Superior Temporal g | Immediate | 4.19 | 48 | -36 | 14 | 0.57 | 1064 | 33.87 |
|  | Delayed | 4.14 | 44 | -34 | 18 | 0.59 | 201 | 6.4 |
|  |  | 3.23 | 66 | -10 | -8 | 0.46 | 66 | 2.1 |
| Angular g | Immediate | 4.33 | 44 | -46 | 22 | 0.59 | 301 | 17.18 |
|  | Delayed | 4.28 | 44 | -46 | 22 | 0.61 | 181 | 10.33 |
| SLF | Immediate | 4.03 | 42 | -46 | 6 | 0.55 | 213 | 25.82 |
|  | Delayed | 4.06 | 38 | -38 | 22 | 0.58 | 175 | 21.21 |
| SupraMarginal g | Immediate | 3.96 | 48 | -40 | 22 | 0.54 | 121 | 6.13 |
|  | Delayed | 4.09 | 46 | -36 | 22 | 0.58 | 61 | 3.09 |
| TR_P_ | Immediate | 4.34 | 34 | -46 | 18 | 0.59 | 121 | 24.85 |
|  | Delayed | 3.95 | 34 | -46 | 18 | 0.56 | 22 | 4.52 |
| CR_S_ | Immediate | 3.48 | 26 | -8 | 30 | 0.47 | 95 | 10.33 |
|  | Delayed | 3.74 | 28 | -10 | 22 | 0.53 | 125 | 13.59 |
| CR_P_ | Immediate | 3.77 | 34 | -40 | 20 | 0.51 | 85 | 18.81 |
|  | Delayed | 4 | 32 | -42 | 24 | 0.57 | 73 | 16.15 |
| Middle Occipital g | Immediate | 3.52 | 46 | -64 | 28 | 0.48 | 76 | 3.62 |
|  | Delayed | - | - | - | - | - | - | - |
| Inferior Temporal g | Immediate | 3.88 | 50 | -44 | -6 | 0.53 | 72 | 2.02 |
|  | Delayed | - | - | - | - | - | - | - |
| SS | Immediate | 3.92 | 38 | -18 | -6 | 0.53 | 72 | 25.17 |
|  | Delayed | - | - | - | - | - | - | - |
| Putamen | Immediate | 3.37 | 32 | -8 | -4 | 0.46 | 55 | 5.17 |
|  | Delayed | 3.72 | 28 | -10 | 12 | 0.53 | 104 | 9.77 |
| IC_RL_ | Immediate | 3.8 | 34 | -34 | 14 | 0.52 | 55 | 17.41 |
|  | Delayed | 3.69 | 30 | -28 | 16 | 0.53 | 29 | 9.18 |
| TP_M_ | Immediate | 3.24 | 44 | 8 | -30 | 0.44 | 48 | 4.04 |
|  | Delayed | - | - | - | - | - | - | - |
| FO_I_ | Immediate | 3.43 | 28 | 12 | -12 | 0.47 | 40 | 15.21 |
|  | Delayed | - | - | - | - | - | - | - |
| EC | Immediate | 3.03 | 32 | -14 | 4 | 0.41 | 32 | 6.87 |
|  | Delayed | 3.41 | 32 | -16 | 8 | 0.49 | 106 | 22.75 |
| TP_S_ | Immediate | 3.27 | 46 | 4 | -20 | 0.44 | 31 | 2.32 |
|  | Delayed | - | - | - | - | - | - | - |
| Hippocampus | Immediate | 3.34 | 42 | -16 | -14 | 0.45 | 30 | 3.17 |
|  | Delayed | - | - | - | - | - | - | - |
| Amygdala | Immediate | 3.41 | 28 | 2 | -14 | 0.46 | 20 | 8.06 |
|  | Delayed | - | - | - | - | - | - | - |
| Insula | Immediate | - | - | - | - | - | - | - |
|  | Delayed | 3.68 | 34 | -16 | 14 | 0.53 | 101 | 5.71 |
| IC_PL_ | Immediate | - | - | - | - | - | - | - |
|  | Delayed | 3.98 | 26 | -10 | 12 | 0.57 | 65 | 12.97 |
| Pallidum | Immediate | - | - | - | - | - | - | - |
|  | Delayed | 3.87 | 24 | 0 | 2 | 0.55 | 60 | 21.43 |

Brain regions defined by the AAL and WM atlases with ‘significant’ clusters of ≥ 20 voxels. Presented results correspond to a z score of 2.6, )*p* < .005). The analysis for this measure has also passed FDR correction for multiple comparisons (corresponding to a z score of 2.42 and 3.24, for Immediate and delayed location memory, respectively). Regions are sorted by the number of ‘significant’ voxels. X, Y, Z coordinates indicate the voxel that is most superior, posterior, and left in its location within the cluster.

**Table S6. VLSM analysis, RHD group: Voxel clusters where damage significantly affected action memory.**

| Structure | Test phase | Z-value | X | Y | Z | Cohen’s r | Voxels | % Area |
| --- | --- | --- | --- | --- | --- | --- | --- | --- |
| Superior Temporal g | Immediate | 3.73 | 50 | -38 | 20 | 0.51 | 152 | 4.84 |
|  |  | 3.32 | 66 | -20 | 4 | 0.45 | 52 | 1.66 |
|  | Delayed | - | - | - | - | - | - | - |
| SLF | Immediate | 3.54 | 38 | -40 | 24 | 0.48 | 114 | 13.82 |
|  | Delayed | - | - | - | - | - | - | - |
| Angular g | Immediate | 3.49 | 42 | -50 | 28 | 0.47 | 90 | 5.14 |
|  | Delayed | 3.43 | 42 | -50 | 28 | 0.49 | 41 | 2.34 |
| Pallidum | Immediate | 3.41 | 20 | 6 | -4 | 0.46 | 64 | 22.86 |
|  | Delayed | 3.77 | 28 | -6 | -6 | 0.54 | 25 | 8.93 |
| CR_S_ | Immediate | 3.7 | 24 | -8 | 30 | 0.50 | 63 | 6.85 |
|  | Delayed | - | - | - | - | - | - | - |
| CR_P_ | Immediate | 3.81 | 28 | -40 | 28 | 0.52 | 63 | 13.94 |
|  | Delayed | - | - | - | - | - | - | - |
| Putamen | Immediate | 3.49 | 22 | 12 | -2 | 0.47 | 47 | 4.42 |
|  | Delayed | - | - | - | - | - | - | - |
| Middle Temporal g | Immediate | 3.04 | 50 | -70 | 18 | 0.41 | 41 | 0.93 |
|  |  | 2.97 | 68 | -26 | -8 | 0.40 | 30 | 0.68 |
|  |  | 3.1 | 42 | -52 | 18 | 0.42 | 26 | 0.59 |
|  |  | 3.14 | 68 | -22 | -18 | 0.43 | 24 | 0.54 |
|  | Delayed | - | - | - | - | - | - | - |
| Inferior Temporal g | Immediate | 3.38 | 56 | -26 | -18 | 0.46 | 30 | 0.84 |
|  | Delayed | - | - | - | - | - | - | - |
| EC | Immediate | 3.42 | 32 | -12 | 8 | 0.47 | 27 | 5.79 |
|  | Delayed | 3.37 | 32 | -12 | 14 | 0.48 | 42 | 9.01 |
| IFG_OP_ | Immediate | 3.31 | 36 | 4 | 28 | 0.45 | 26 | 1.86 |
|  | Delayed | - | - | - | - | - | - | - |
| TR_P_ | Immediate | 3.97 | 34 | -42 | 18 | 0.54 | 23 | 4.72 |
|  | Delayed | - | - | - | - | - | - | - |
| Caudate | Immediate | 3.39 | 20 | 22 | 14 | 0.46 | 22 | 2.21 |
|  | Delayed | - | - | - | - | - | - | - |
| SupraMarginal g | Immediate | 3.11 | 44 | -36 | 22 | 0.42 | 22 | 1.11 |
|  | Delayed | - | - | - | - | - | - | - |
| Insula | Immediate | - | - | - | - | - | - | - |
|  | Delayed | 3.53 | 34 | -16 | 14 | 0.50 | 21 | 1.19 |
| IC_PL_ | Immediate | 3.2 | 26 | -10 | 12 | 0.44 | 21 | 4.19 |
|  | Delayed | - | - | - | - | - | - | - |
| FO_I_ | Immediate | 3.58 | 26 | 12 | -12 | 0.49 | 21 | 7.98 |
|  | Delayed | - | - | - | - | - | - | - |

Brain regions defined by the AAL and WM atlases with ‘significant’ clusters of ≥ 20 voxels. Presented results correspond in this analysis to a z score of 2.6, (*p* < .005). Regions are sorted by the number of ‘significant’ voxels. X, Y, Z coordinates indicate the voxel that is most superior, posterior, and left in its location within the cluster.

**Table S7. VLSM analysis, LHD group: Voxel clusters where damage significantly affected identity memory.**

| Structure | Test phase | Z-value | X | Y | Z | Voxels | Cohen’s r | % Area |
| --- | --- | --- | --- | --- | --- | --- | --- | --- |
| Fusiform | Immediate | 2.92 | -36 | -34 | -18 | 20 | 0.47 | 0.87 |
|  | Delayed | 2.97 | -36 | -34 | -18 | 20 | 0.50 | 0.87 |

Brain regions defined by the AAL and WM atlases with ‘significant’ clusters of ≥ 20 voxels. Presented results correspond in this analysis to z scores of 2.6, (*p* < .005). Regions are sorted by the number of ‘significant’ voxels. X, Y, Z coordinates indicate the voxel that is most superior, posterior, and left in its location within the cluster.

**Table S8. VLSM analysis, LHD group: Voxel clusters where damage significantly affected location memory.**

| Structure | Test phase | Z-value | X | Y | Z | Cohen’s r | Voxels | % Area |
| --- | --- | --- | --- | --- | --- | --- | --- | --- |
| Lingual g | Immediate | - | - | - | - | - | - | - |
|  | Delayed | 2.72 | -86 | -18 | -26 | 0.45 | 729 | 34.8 |
| Calcarine | Immediate | - | - | - | - | - | - | - |
|  | Delayed | 2.72 | -86 | -6 | -8 | 0.45 | 333 | 14.75 |
| Fusiform g | Immediate | - | - | - | - | - | - | - |
|  | Delayed | 2.72 | -78 | -18 | -22 | 0.45 | 205 | 8.87 |
| ParaHippocampal g | Immediate | - | - | - | - | - | - | - |
|  | Delayed | 2.72 | -28 | -14 | -24 | 0.45 | 109 | 11.15 |
| Inferior Occipital g | Immediate | - | - | - | - | - | - | - |
|  | Delayed | 2.72 | -80 | -12 | -26 | 0.45 | 92 | 9.78 |
| Hippocampus | Immediate | - | - | - | - | - | - | - |
|  | Delayed | 2.72 | -28 | -12 | -28 | 0.45 | 22 | 2.36 |

Brain regions defined by the AAL and WM atlases with ‘significant’ clusters of ≥20 voxels. Presented results correspond in this analysis to z scores of 2.6, (*p* < .005). Results of the delayed location measure have also passed FDR correction for multiple comparisons (corresponding to a z score of 2.58). Regions are sorted by the number of ‘significant’ voxels. X, Y, Z coordinates indicate the voxel that is most superior, posterior, and left in its location within the cluster.

**Table S9. VLSM analysis, LHD group: Voxel clusters where damage significantly affected action memory.**

| Structure | Test phase | Z-value | X | Y | Z | Cohen’s r | Voxels | % Area |
| --- | --- | --- | --- | --- | --- | --- | --- | --- |
| Lingual g | Immediate | 2.87 | -26 | -86 | -18 | 0.46 | 726 | 34.65 |
|  | Delayed | 2.74 | -28 | -62 | -4 | 0.46 | 729 | 34.8 |
| Calcarine | Immediate | 2.87 | -8 | -86 | -6 | 0.46 | 333 | 14.75 |
|  | Delayed | 2.74 | -8 | -86 | -6 | 0.46 | 333 | 14.75 |
| Fusiform g | Immediate | 2.87 | -22 | -78 | -18 | 0.46 | 203 | 8.79 |
|  | Delayed | 2.74 | -30 | -62 | -4 | 0.46 | 205 | 8.87 |
| ParaHippocampal g | Immediate | 2.87 | -24 | -28 | -14 | 0.46 | 107 | 10.94 |
|  | Delayed | 2.74 | -34 | -44 | -8 | 0.46 | 109 | 11.15 |
| Inferior Occipital g | Immediate | 2.87 | -26 | -80 | -12 | 0.46 | 92 | 9.78 |
|  | Delayed | 2.74 | -26 | -80 | -12 | 0.46 | 92 | 9.78 |
| Insula | Immediate | - | - | - | - |  | - | - |
|  | Delayed | 2.84 | -26 | 32 | 2 | 0.47 | 56 | 3.01 |
| CR_A_ | Immediate | - | - | - | - |  | - | - |
|  | Delayed | 2.84 | -24 | 34 | 12 | 0.47 | 53 | 6.13 |
| Hippocampus | Immediate | - | - | - | - |  | - | - |
|  | Delayed | 2.74 | -26 | -28 | -8 | 0.46 | 22 | 2.36 |
| Insula | Immediate | - | - | - | - |  | - | - |
|  | Delayed | 3.18 | -32 | 0 | 16 | 0.53 | 20 | 1.08 |

Brain regions defined by the AAL and WM atlases with ‘significant’ clusters of ≥ 10 voxels. Presented results correspond in this analysis to z scores of 2.6, (*p* < .005). Results have also passed FDR correction for multiple comparisons (corresponding to a z score of 2.69 and 2.53 for immediate and delayed action memory, respectively). Regions are sorted by the number of ‘significant’ voxels. X, Y, Z coordinates indicate the voxel that is most superior, posterior, and left in its location within the cluster.

**Table S10. VLSM conjunction analysis in the Immediate and Delayed testing phases for the RHD and LHD groups.**

1. RHD group

|  |  | Identity vs. Location vs. Action | | | | |  |  |  |
| --- | --- | --- | --- | --- | --- | --- | --- | --- | --- |
| Region | Test phase | I | L | A | I+L | I+A | L+A | I+L+A |  |
| Precentral G | Immediate | 25 | - | - | - | - | - | - |  |
|  | Delayed | - | - | - | - | - | - | - |  |
| IFG_OP_ | Immediate | 27 | - | - | - | - | - | - |  |
|  | Delayed | - | - | - | - | - | - | - |  |
| Insula | Immediate | 57 | - | - | - | - | - | - |  |
|  | Delayed | 40 | 48 | - | 53 | 21 | 22 | - |  |
| Hippocampus | Immediate | 42 | - | - | 29 | - | - | - |  |
|  | Delayed | 31 | - | - | - | - | - | - |  |
| Middle Occipital g | Immediate | - | 26 | - | 53 | 23 | 28 | - |  |
|  | Delayed | 23 | 27 | - | - | - | - | - |  |
| Inferior Parietal lobule | Immediate | 31 | - | - | - | - | - | - |  |
|  | Delayed | 25 | - | - | - | - | - | - |  |
| SupraMarginal g | Immediate | 50 | 31 | - | 90 | 22 | 21 | 20 |  |
|  | Delayed | 109 | - | - | 51 | - | - | - |  |
| Angular g | Immediate | 71 | 61 | - | 240 | 90 | 94 | 88 |  |
|  | Delayed | 108 | 67 | - | 115 | 42 | 42 | 42 |  |
| Caudate | Immediate | - | - | 27 | - | - | - | - |  |
|  | Delayed | 25 | - | - | - | - | - | - |  |
| Putamen | Immediate | 53 | - | 43 | 47 | 23 | - | - |  |
|  | Delayed | 95 | 58 | - | 53 | - | - | - |  |
| Pallidum | Immediate | - | - | 52 | - | - | - | - |  |
|  | Delayed | - | 33 | - | - | - | 25 | - |  |
| Heschl g | Immediate | - | - | - | - | - | - | - |  |
|  | Delayed | 54 | - | - | - | - | - | - |  |
| Superior Temporal g | Immediate | 77 | 515 | - | 512 | 196 | 231 | 189 |  |
|  | Delayed | 1281 | 61 | - | 264 | - | - | - |  |
| TP_S_ | Immediate | - | 40 | - | - | - | - | - |  |
|  | Delayed | - | 32 | - | - | - | - | - |  |
| Middle Temporal g | Immediate | 121 | 709 | 34 | 876 | 129 | 145 | 127 |  |
|  | Delayed | 299 | 76 | - | 68 | 11 | - | - |  |
| TP_M_ | Immediate | - | 48 | - | - | - | - | - |  |
|  | Delayed | - | 19 | - | - | - | - | - |  |
| Inferior Temporal g | Immediate | - | 43 | 26 | - | - | - | - |  |
|  | Delayed | 40 | - | - | - | - | - | - |  |
| IC_AL_ | Immediate | - | - | 23 | - | - | - | - |  |
|  | Delayed | 26 | - | - | - | - | - | - |  |
| IC_PL_ | Immediate | 85 | - | - | - | 24 | - | - |  |
|  | Delayed | 26 | 42 | - | 26 | - | - | - |  |
| IC_RL_ | Immediate | 60 | - | - | 52 | 20 | - | - |  |
|  | Delayed | 70 | - | - | - | - | - | - |  |
| CR_A_ | Immediate | - | - | 21 | - | - | - | - |  |
|  | Delayed | - | - | - | - | - | - | - |  |
| CR_S_ | Immediate | 110 | - | - | 79 | 63 | 55 | 51 |  |
|  | Delayed | 36 | 88 | - | 38 | - | - | - |  |
| CR_P_ | Immediate | 46 | - | - | 73 | 45 | 46 | 43 |  |
|  | Delayed | 55 | 28 | - | 42 | - | - | - |  |
| TR_P_ | Immediate | - | 35 | - | 85 | 33 | 33 | 32 |  |
|  | Delayed | 30 | - | - | - | - | - | - |  |
| SS | Immediate | 64 | - | - | 68 | - | - | - |  |
|  | Delayed | 47 | - | - | - | - | - | - |  |
| EC | Immediate | 47 | - | - | 28 | - | - | - |  |
|  | Delayed | 50 | 38 | - | 62 | 26 | 43 | 26 |  |
| SLF | Immediate | 76 | - | - | 219 | 121 | 110 | 108 |  |
|  | Delayed | 85 | 48 | - | 114 | - | - | - |  |
| FO_I_ | Immediate | 24 | - | - | 31 | - | - | - |  |
|  | Delayed | 58 | - | - | - | - | - | - |  |
| BCC | Immediate | 20 | - | - | - | - | - | - |  |
|  | Delayed | - | - | - | - | - | - | - |  |

1. LHD group

|  |  | | Identity vs. Location vs. Action | | | | |  |  |
| --- | --- | --- | --- | --- | --- | --- | --- | --- | --- |
| Region | | Test phase | I | L | A | I+L | I+A | L+A | I+L+A |
| Insula | | Immediate | - | - | - | - | - | - | - |
|  | | Delayed | - | - | 95 | - | - | - | - |
| Hippocampus | | Immediate | - |  | - | 102 | - | - | - |
|  | | Delayed | - | - | - | - | - | 22 | - |
| ParaHippocampal g | | Immediate | - | - | - | - | - | - | - |
|  | | Delayed | - | - | - | - | - | 109 | - |
| Calcarine | | Immediate | - | - | - | - | - | - | - |
|  | | Delayed | - | - | - | - | - | 333 | - |
| Lingual g | | Immediate | - | - | - | - | - | - | - |
|  | | Delayed | - | - | - | - | - | 729 | - |
| Inferior Occipital g | | Immediate | - | - | - | - | - | - | - |
|  | | Delayed | - | - | - | - | - | 92 | - |
| Fusiform g | | Immediate | 20 | - | - | - | - | - | - |
|  | | Delayed | 20 | - | - | - | - | 205 | - |
| IC_AL_ | | Immediate | 23 | - | - | - | - | - | - |
|  | | Delayed | - | - | - | - | - | - | - |
| IC_PL_ | | Immediate | - | 32 | - | - | - | - | - |
|  | | Delayed | - | - | - | - | - | - | - |

Voxel clusters in which the existence of damage significantly affected the following measures of visual memory, using the coding I = identity, L = location, A = action, for the following functions: identity only, location only, action only, identity plus location, identity plus action, location plus action, and identity plus location plus action. Brain regions defined by the AAL and WM atlases with clusters of ≥ 10 significant voxels correspond to a z score of 2.6, (*p* < .005) are presented.

**Table S12**. **Extent of damage (%) in brain regions defined by the AAL and WM atlases for individual RHD and LHD patients.**

**A. Right hemisphere damage (RHD) group**

| Areas | 1001 | | 1002 | 1003 | 1004 | 1005 | 1006 | 1007 | 1008 | 1009 | 1010 | 1011 | 1012 | 1013 | 1014 | 1015 | 1016 | 1017 | 1018 | 1019 |
| --- | --- | --- | --- | --- | --- | --- | --- | --- | --- | --- | --- | --- | --- | --- | --- | --- | --- | --- | --- | --- |
| Precentral g | 0 | | 71.28 | 58.56 | 6.71 | 1.04 | 0.71 | 22.33 | 33.78 | 6.48 | 11.48 | 81.6 | 14.49 | 5.62 | 68.74 | 3.67 | 1.98 | 0.86 | 2.81 | 61.85 |
| Superior frontal g | 0 | | 3.57 | 63.17 | 0.02 | 0 | 0 | 0 | 0.02 | 0 | 13.19 | 9.89 | 0 | 0 | 4.17 | 0 | 0 | 0 | 1.5 | 20.12 |
| Sup. frontal g, orbital | 0 | | 28.89 | 38.01 | 1.5 | 0 | 0 | 0 | 19.26 | 0 | 20.46 | 0 | 0 | 0 | 56.17 | 0 | 0 | 1 | 2.11 | 0 |
| Mid. frontal g | 0 | | 57.23 | 88.6 | 9.5 | 0 | 0 | 7.84 | 11.36 | 0 | 11.21 | 34.37 | 0 | 0.43 | 67.81 | 0.69 | 0 | 0.67 | 4.94 | 11.19 |
| Mid. frontal g, med. orb. | 0 | | 70.94 | 59.41 | 0.1 | 0 | 0 | 0 | 62.27 | 0 | 7.98 | 0 | 0 | 0 | 81.58 | 0 | 0 | 0 | 1.67 | 0 |
| IFG_OP_ | 2.64 | | 90.42 | 22.94 | 76.13 | 0 | 3.57 | 48.96 | 88.21 | 6.22 | 20.23 | 90.85 | 0 | 25.88 | 99.07 | 34.6 | 1.86 | 40.17 | 51.47 | 0 |
| IFG_TRI_ | 0.65 | | 93.49 | 18.18 | 53.28 | 0 | 1.86 | 2.46 | 77.27 | 0 | 19.67 | 21.8 | 0 | 0.23 | 99.91 | 9.9 | 0 | 15.9 | 41.56 | 0 |
| IFG_ORB_ | 4.8 | | 98.01 | 5.92 | 28.76 | 0 | 0 | 22.96 | 72.52 | 0 | 2.46 | 2.99 | 0 | 0 | 98.36 | 17.93 | 0 | 17.46 | 31.69 | 0 |
| Rolandic op. | 29.6 | | 95.94 | 0 | 89.71 | 0 | 16.53 | 88.13 | 95.27 | 26.07 | 11.8 | 91.36 | 1.8 | 75.13 | 100 | 57.02 | 76.4 | 18.26 | 87.83 | 2.48 |
| Supp. motor area | 0 | | 0 | 8.73 | 0 | 3.54 | 0 | 0 | 0 | 0 | 2.91 | 0 | 5.74 | 0 | 0 | 0 | 0 | 0 | 0 | 47.66 |
| Olfactory | 0 | | 11.42 | 0 | 0.69 | 0 | 4.84 | 0 | 1.73 | 0 | 2.77 | 0 | 0 | 0 | 23.53 | 0 | 0 | 4.5 | 8.65 | 0 |
| Sup. frontal g, medial | 0 | | 0 | 15.65 | 0 | 0 | 0 | 0 | 0 | 0 | 6.28 | 0 | 0 | 0 | 0.09 | 0 | 0 | 0 | 0.94 | 0 |
| Sup. frontal g, orbital | 0 | | 0 | 10.63 | 0 | 0 | 0 | 0 | 1.29 | 0 | 26.64 | 0 | 0 | 0 | 8.18 | 0 | 0 | 0 | 0.47 | 0 |
| Rectus g | 0 | | 2.68 | 0.13 | 0 | 0 | 0 | 0 | 0.13 | 0 | 2.28 | 0 | 0 | 0 | 24.3 | 0 | 0 | 0 | 2.01 | 0 |
| Insula | 58.42 | | 99.89 | 1.07 | 98.53 | 0 | 17.68 | 63.5 | 99.32 | 3.95 | 6.89 | 79.32 | 1.53 | 61.53 | 100 | 85.08 | 41.5 | 69.89 | 95.76 | 0.23 |
| Anterior cingulum | 0 | | 0.08 | 0 | 0 | 0 | 0 | 0 | 0 | 0 | 21.17 | 0 | 0 | 0 | 3.05 | 0 | 0 | 0 | 4.8 | 3.35 |
| Middle cingulum | 0 | | 0.18 | 0.23 | 0 | 21.83 | 0 | 0 | 0 | 0 | 11.58 | 0 | 4.68 | 0.32 | 0 | 0 | 0.05 | 0 | 0 | 47.48 |
| Posterior cingulum | 0 | | 0 | 0 | 0 | 0 | 0 | 0 | 0 | 0 | 0 | 0 | 0 | 2.69 | 0 | 0 | 0 | 0 | 0 | 0 |
| Hippocampus | 14.16 | | 0 | 0 | 11.73 | 0 | 0.21 | 0.63 | 0.32 | 0 | 0 | 2.33 | 0 | 15.33 | 1.8 | 0 | 5.92 | 0.11 | 4.44 | 0 |
| Parahippocampal g | 0 | | 1.24 | 0 | 0.71 | 0 | 0 | 4.59 | 0 | 0 | 0 | 0 | 0 | 0.18 | 6.54 | 0 | 0 | 0 | 0 | 0 |
| Amygdala | 3.23 | | 6.05 | 0 | 13.31 | 0 | 26.21 | 5.65 | 5.65 | 0 | 0 | 5.24 | 0 | 1.21 | 37.9 | 0 | 0 | 13.71 | 7.26 | 0 |
| Calcarine | 0 | | 0.7 | 0 | 0 | 0 | 0 | 0.05 | 0 | 0 | 0 | 0 | 0 | 2.85 | 0 | 0 | 0 | 0 | 0 | 0 |
| Cuneus | 0 | | 0.07 | 0.14 | 0 | 0 | 0.63 | 0 | 0 | 0 | 0 | 1.4 | 0 | 0.21 | 0 | 0 | 0 | 0 | 0 | 0 |
| Lingual g | 0 | | 0 | 0 | 0 | 0 | 0 | 0 | 0 | 0 | 0 | 0 | 0 | 2.3 | 0 | 0 | 0 | 0 | 0 | 0 |
| Superior occipital g | 1.06 | | 0.07 | 1.77 | 0 | 0.28 | 1.77 | 0 | 0 | 0 | 0 | 8.7 | 0 | 1.49 | 0 | 0 | 0 | 0 | 0 | 0.57 |
| Middle occipital g | 7.29 | | 14.87 | 10.1 | 0.24 | 0 | 5.2 | 2.48 | 0 | 0 | 1.76 | 5.05 | 0 | 3.24 | 27.55 | 0 | 0.71 | 0 | 0 | 0 |
| Inferior occipital g | 0 | | 1.31 | 0 | 0 | 0 | 0 | 0 | 0 | 0 | 0 | 0 | 0 | 0 | 17.9 | 0 | 0 | 0 | 0 | 0 |
| Fusiform g | 0.08 | | 1.07 | 0 | 4.01 | 0 | 0 | 3.02 | 0 | 0 | 0 | 0 | 0 | 2.14 | 2.1 | 0 | 0 | 0 | 0.28 | 0 |
| Postcentral g | 4.05 | | 85.56 | 59.85 | 3.3 | 14.54 | 0.18 | 6.25 | 37.35 | 7.59 | 7.38 | 82.47 | 62.23 | 10.28 | 68.43 | 10.62 | 12.1 | 0 | 9.84 | 59.74 |
| Superior parietal g | 0.09 | | 32.18 | 69.67 | 0 | 34.97 | 0 | 0 | 0 | 0 | 0.05 | 25.52 | 12.96 | 0 | 8.28 | 0.14 | 0 | 0 | 0 | 30.38 |
| Inferior parietal g | 20.52 | | 96.88 | 86.02 | 0 | 7.06 | 0 | 0.22 | 2.97 | 0 | 1.26 | 94.35 | 17.55 | 16.21 | 64.76 | 22.45 | 0.22 | 0 | 18.74 | 1.41 |
| Supramarginal g | 34.8 | | 96.61 | 15.4 | 10.28 | 0.51 | 12.82 | 61.96 | 84.14 | 2.33 | 17.98 | 96.1 | 18.24 | 73.91 | 98.18 | 68.69 | 63.6 | 0 | 59.02 | 3.55 |
| Angular g | 67.18 | | 73.52 | 15.07 | 3.88 | 1.26 | 10.96 | 22.09 | 6.56 | 0 | 15.58 | 82.82 | 0 | 37.04 | 68.84 | 5.19 | 7.65 | 0 | 1.03 | 0.23 |
| Precuneus | 0.03 | | 0.34 | 7.26 | 0 | 13.48 | 0 | 0 | 0 | 0 | 0 | 0.25 | 5.11 | 0.61 | 0 | 0 | 0 | 0 | 0.12 | 33.45 |
| Paracentral lobule | 0 | | 0 | 37.32 | 0 | 20.22 | 0 | 0 | 0 | 0 | 1.2 | 0.24 | 52.87 | 0 | 0 | 0 | 0 | 0 | 0 | 80.38 |
| Caudate | 19.62 | | 13.28 | 29.78 | 0.3 | 0 | 50.8 | 0 | 1.81 | 0 | 17 | 1.51 | 0 | 0 | 73.24 | 2.52 | 0 | 42.86 | 66.2 | 0.4 |
| Putamen | 56.77 | | 68.7 | 47.09 | 30.36 | 0 | 84.87 | 21.05 | 75 | 0 | 25.56 | 24.53 | 0 | 10.62 | 100 | 55.92 | 12.4 | 92.11 | 93.14 | 7.71 |
| Pallidum | 25.36 | | 52.86 | 4.29 | 2.86 | 0 | 76.43 | 11.43 | 19.64 | 0 | 71.43 | 0 | 0 | 8.57 | 98.57 | 4.64 | 1.07 | 64.64 | 48.57 | 0 |
| Thalamus | 17.6 | | 0.95 | 0.09 | 1.51 | 0 | 7.28 | 0 | 0 | 0 | 0.28 | 0 | 0 | 0.38 | 12.68 | 0 | 0.95 | 2.27 | 4.26 | 0 |
| Heschl g | 69.48 | | 98.39 | 0 | 90.36 | 0 | 6.83 | 93.98 | 96.39 | 0 | 2.41 | 97.19 | 0 | 94.78 | 100 | 71.49 | 90.8 | 2.81 | 97.99 | 0 |
| Superior temporal g | 69.15 | | 91.02 | 0.38 | 76.06 | 0 | 8.28 | 94.08 | 54.95 | 0 | 5.44 | 59.57 | 0 | 80.74 | 99.87 | 22.83 | 45.2 | 2.42 | 60.9 | 0 |
| Superior temporal pole | 28.33 | | 86.55 | 0 | 62.11 | 0 | 0 | 86.32 | 56.13 | 0 | 0 | 12.71 | 0 | 6.65 | 98.8 | 16.07 | 0.07 | 3.66 | 19.36 | 0 |
| Middle temporal g | 18.19 | | 75.01 | 6.53 | 60.35 | 0 | 5.15 | 78.16 | 25.65 | 0 | 1.38 | 11.54 | 0 | 37.06 | 97.85 | 0.02 | 20.7 | 0 | 3.79 | 0 |
| Middle temporal pole | 4.63 | | 82.14 | 0 | 37.41 | 0 | 0 | 86.6 | 34.12 | 0 | 0 | 0 | 0 | 0 | 95.45 | 0 | 0 | 0 | 0.25 | 0 |
| Inferior temporal g | 0.39 | | 11.5 | 0 | 16.02 | 0 | 0 | 41.41 | 14.39 | 0 | 0 | 0 | 0 | 1.8 | 42.11 | 0 | 0.03 | 0 | 2.45 | 0 |
| CST | 0 | | 0 | 0 | 0 | 0 | 0 | 0 | 0 | 0 | 0 | 0 | 0 | 0 | 0 | 0 | 0 | 0 | 0 | 0 |
| ML | 0 | | 0 | 0 | 0 | 0 | 0 | 0 | 0 | 0 | 0 | 0 | 0 | 0 | 0 | 0 | 0 | 0 | 0 | 0 |
| ICP | 0 | | 0 | 0 | 0 | 0 | 0 | 0 | 0 | 0 | 0 | 0 | 0 | 0 | 0 | 0 | 0 | 0 | 0 | 0 |
| SCP | 0 | | 0 | 0 | 0 | 0 | 0 | 0 | 0 | 0 | 0 | 0 | 0 | 0 | 0 | 0 | 0 | 0 | 0 | 0 |
| CP | 14.93 | | 0 | 0 | 0 | 0 | 0 | 1.12 | 0 | 0 | 0 | 0 | 0 | 1.12 | 0 | 0 | 0 | 0 | 0 | 0 |
| IC_AL_ | 20.64 | | 65.6 | 57 | 1.97 | 0 | 84.77 | 0 | 15.97 | 0 | 26.29 | 0 | 0 | 2.21 | 93.61 | 25.55 | 0 | 86.24 | 87.71 | 2.95 |
| IC_PL_ | 77.84 | | 33.93 | 30.74 | 19.56 | 0 | 64.67 | 0 | 19.36 | 0 | 29.74 | 3.19 | 1.6 | 12.57 | 78.44 | 14.77 | 13 | 53.09 | 58.48 | 0.4 |
| IC_RL_ | 86.08 | | 35.13 | 0 | 25.95 | 0 | 12.34 | 10.76 | 11.71 | 0 | 16.14 | 0.32 | 0 | 59.81 | 31.65 | 6.96 | 77.2 | 7.91 | 65.51 | 0 |
| CR_A_ | 0 | | 49.77 | 27.22 | 34.58 | 0 | 10.51 | 0 | 27.69 | 0 | 90.42 | 3.5 | 0 | 4.32 | 89.95 | 0.35 | 0 | 58.29 | 73.71 | 1.87 |
| CR_S_ | 46.41 | | 81.2 | 22.93 | 48.7 | 14.35 | 54.24 | 6.09 | 52.61 | 28.04 | 88.15 | 37.5 | 22.17 | 34.35 | 56.96 | 34.35 | 33.8 | 55.33 | 57.17 | 38.37 |
| CR_P_ | 52.21 | | 56.64 | 4.87 | 25.88 | 23.67 | 11.73 | 4.87 | 5.31 | 5.97 | 64.16 | 25 | 6.64 | 62.83 | 18.58 | 30.09 | 43.1 | 19.91 | 42.04 | 16.37 |
| TR_P_ | 9.86 | | 12.53 | 0 | 8.42 | 0 | 0 | 3.49 | 0 | 0 | 6.57 | 0 | 0 | 55.03 | 20.53 | 0 | 36.6 | 0 | 5.34 | 0 |
| SS | 52.45 | | 11.19 | 0 | 21.68 | 0 | 0 | 15.03 | 2.45 | 0 | 0 | 0 | 0 | 53.85 | 17.83 | 0 | 34.6 | 0 | 16.43 | 0 |
| External capsule | 84.12 | | 96.35 | 54.72 | 68.03 | 0 | 92.7 | 31.33 | 97.42 | 1.29 | 20.82 | 18.03 | 1.29 | 37.12 | 100 | 78.33 | 46.8 | 95.92 | 100 | 18.03 |
| CGC | 0 | | 0 | 0 | 0 | 6.8 | 0 | 0 | 0 | 0 | 2.04 | 0 | 0 | 0.68 | 0 | 0 | 0 | 0 | 0 | 32.31 |
| CGH | 0 | | 0 | 0 | 0 | 0 | 0 | 0 | 0 | 0 | 0 | 0 | 0 | 0 | 0 | 0 | 0 | 0 | 0 | 0 |
| FX/ST | 30.66 | | 2.19 | 0 | 0 | 0 | 0 | 5.11 | 0 | 0 | 0 | 0 | 0 | 32.12 | 0 | 0 | 4.38 | 0 | 0.73 | 0 |
| SLF | 50.3 | | 90.42 | 18.79 | 45.33 | 1.82 | 22.91 | 44.48 | 73.45 | 33.09 | 80.73 | 80.12 | 40.61 | 97.33 | 77.33 | 66.79 | 83.3 | 12.97 | 72 | 15.76 |
| FOs | 76.27 | | 93.22 | 62.71 | 0 | 0 | 100 | 0 | 10.17 | 0 | 47.46 | 0 | 0 | 0 | 54.24 | 27.12 | 1.69 | 100 | 100 | 5.08 |
| FO_I_ | 62.74 | | 77.57 | 10.27 | 37.64 | 0 | 64.26 | 49.05 | 72.24 | 0 | 0.38 | 0 | 0 | 27.38 | 98.86 | 29.66 | 15.6 | 73.76 | 83.65 | 0 |
| UNC | 44.68 | | 57.45 | 0 | 36.17 | 0 | 46.81 | 80.85 | 91.49 | 0 | 0 | 0 | 0 | 6.38 | 100 | 19.15 | 17 | 48.94 | 65.96 | 0 |
| TAP | 19.23 | | 11.54 | 0 | 0 | 0 | 0 | 0 | 0 | 0 | 16.67 | 0 | 0 | 5.13 | 0 | 0 | 10.3 | 0 | 8.97 | 0 |
| PCT - bilateral | 0 | | 0 | 0 | 0 | 0 | 0 | 0 | 0 | 0 | 0 | 0 | 0 | 0 | 0 | 0 | 0 | 0 | 0 | 0 |
| BCC - bilateral | 0 | | 1.51 | 0.06 | 0 | 0.87 | 0.12 | 0 | 0 | 0 | 14.71 | 0 | 0.35 | 0 | 3.88 | 0.06 | 0.29 | 0.23 | 2.55 | 14.07 |
| FX - bilateral | 0 | | 0 | 0 | 0 | 0 | 0 | 0 | 0 | 0 | 0 | 0 | 0 | 0 | 0 | 0 | 0 | 0 | 0 | 0 |
| MCP - bilateral | 0 | | 0 | 0 | 0 | 0 | 0 | 0 | 0 | 0 | 0 | 0 | 0 | 0 | 0 | 0 | 0 | 0 | 0 | 0 |
| GCC - bilateral | 0 | | 3.45 | 5.13 | 0 | 0 | 0 | 0 | 0 | 0 | 20.16 | 0 | 0 | 0 | 22.37 | 0 | 0 | 1.33 | 10.43 | 3.36 |
| SCC - bilateral | 0.19 | | 1.88 | 0 | 0 | 0 | 0 | 0 | 0 | 0 | 0.06 | 0 | 0 | 4.28 | 0 | 0 | 0.06 | 0 | 2.59 | 0 |
| **A. Right hemisphere damage (RHD) group (cont.)** | | | | | | | | | | | | | | | | | | | |  |
| Areas | 1020 | 1021 | 1022 | 1023 | 1024 | 1025 | 1026 | 1027 | 1028 | 1029 | 1030 | 1031 | 1032 | 1033 | 1034 | 1035 | 1036 | 1037 | 1038 |  |
| Precentral g | 7.93 | 0 | 14.23 | 0.12 | 37.62 | 22.89 | 0 | 39.6 | 14.2 | 19.4 | 7.84 | 11.5 | 4.02 | 35.08 | 0.06 | 16.95 | 0 | 14.73 | 0 |  |
| Superior frontal g | 0.02 | 0 | 0.59 | 0.02 | 24.85 | 9.32 | 0 | 16.96 | 3.55 | 0.2 | 5.7 | 0 | 0 | 0.52 | 0 | 0 | 0 | 12.15 | 0 |  |
| Sup. frontal g, orbital | 0 | 0 | 4.81 | 0.1 | 14.64 | 4.31 | 0 | 0 | 0 | 0.7 | 0.3 | 0 | 0 | 0 | 0 | 0 | 0 | 0 | 0 |  |
| Mid. frontal g | 2.04 | 0 | 5.25 | 1.06 | 57.48 | 5.33 | 0 | 3.12 | 0.45 | 10.68 | 4.27 | 2.02 | 4.68 | 33.99 | 0.02 | 18.34 | 0 | 2.16 | 0 |  |
| Mid. frontal g, med. orb. | 0 | 0 | 8.57 | 0.89 | 40.49 | 2.66 | 0 | 0 | 0 | 12.91 | 0 | 0 | 0 | 1.97 | 0 | 0.1 | 0 | 0 | 0 |  |
| IFG_OP_ | 44.89 | 0 | 70.34 | 10.01 | 13.8 | 23.02 | 6.58 | 2.07 | 8.72 | 77.06 | 17.23 | 16.9 | 11.58 | 95.57 | 0 | 93.14 | 0.21 | 0 | 0 |  |
| IFG_TRI_ | 11.34 | 0 | 51.93 | 11.11 | 25.71 | 15.67 | 1.02 | 0.65 | 0 | 70.2 | 7.58 | 3.63 | 18.55 | 79.36 | 0 | 81.08 | 0.14 | 0 | 0 |  |
| IFG_ORB_ | 12.6 | 0.06 | 38.72 | 10.37 | 36.85 | 3.34 | 12.36 | 16.93 | 0 | 36.26 | 0.47 | 0.18 | 0 | 19.27 | 0.88 | 10.95 | 0.94 | 0 | 0 |  |
| Rolandic op. | 89.93 | 1.43 | 77.76 | 75.36 | 1.73 | 40.57 | 8.26 | 54.32 | 12.47 | 92.11 | 28.32 | 14.4 | 9.24 | 93.91 | 0 | 83.02 | 11.87 | 0 | 0 |  |
| Supp. motor area | 0 | 0 | 0 | 0 | 5.19 | 21.81 | 0 | 60.35 | 16.28 | 0 | 3.29 | 0 | 0 | 0 | 0 | 0 | 0 | 32.77 | 0 |  |
| Olfactory | 3.46 | 0 | 0 | 0 | 0 | 2.42 | 2.08 | 0 | 0 | 5.19 | 0 | 0 | 9.34 | 0 | 0 | 0.35 | 0 | 0 | 0 |  |
| Sup. frontal g, medial | 0 | 0 | 0 | 0 | 0.19 | 10.45 | 0 | 0.7 | 0 | 0 | 1.31 | 0 | 0 | 0 | 0 | 0 | 0 | 0 | 0 |  |
| Sup. frontal g, orbital | 0 | 0 | 0 | 0 | 0.35 | 16.82 | 0 | 0 | 0 | 0 | 0 | 0 | 0 | 0 | 0 | 0 | 0 | 0 | 0 |  |
| Rectus g | 0 | 0 | 0 | 0 | 1.07 | 5.91 | 0 | 0 | 0 | 0 | 0 | 0 | 0.27 | 0 | 0 | 0 | 0 | 0 | 0 |  |
| Insula | 86.95 | 20.96 | 74.12 | 93.95 | 12.37 | 49.77 | 68.14 | 36.5 | 1.24 | 68.59 | 57.8 | 8.47 | 11.47 | 85.93 | 0 | 86.16 | 48.31 | 0 | 0 |  |
| Anterior cingulum | 0 | 0 | 0 | 0.08 | 0.99 | 23.08 | 0 | 2.28 | 0 | 0 | 3.27 | 0 | 0 | 0 | 0 | 0 | 0 | 0 | 0 |  |
| Middle cingulum | 0.32 | 0 | 1.04 | 0 | 6.08 | 14.25 | 0 | 24.88 | 10.17 | 0.41 | 3.45 | 0 | 0 | 0 | 0 | 0 | 0 | 8.76 | 0 |  |
| Posterior cingulum | 0 | 0 | 0 | 0 | 0 | 0 | 0 | 0 | 42.09 | 0 | 0 | 0 | 0.3 | 0 | 0 | 0 | 0 | 0 | 0 |  |
| Hippocampus | 15.01 | 0 | 0 | 3.07 | 0 | 14.38 | 50.95 | 0 | 1.9 | 20.93 | 21.04 | 0 | 0 | 0.63 | 0 | 0 | 2.11 | 0 | 0 |  |
| Parahippocampal g | 3.45 | 0 | 0 | 0 | 1.5 | 0.8 | 3.8 | 0 | 0 | 10.42 | 0 | 0 | 0 | 14.66 | 0 | 0 | 0.09 | 0 | 0 |  |
| Amygdala | 25.4 | 0 | 0 | 10.48 | 0 | 1.21 | 30.24 | 0 | 0 | 58.06 | 11.29 | 0 | 7.66 | 73.39 | 0 | 4.03 | 4.44 | 0 | 0 |  |
| Calcarine | 0.21 | 0 | 0 | 0 | 0 | 0 | 0 | 0 | 66.79 | 0 | 6.23 | 6.61 | 0 | 0 | 0 | 0 | 0 | 0 | 0 |  |
| Cuneus | 0 | 0 | 0 | 0 | 1.69 | 0 | 0 | 0 | 78.93 | 0 | 3.79 | 3.02 | 0.63 | 0 | 0 | 0 | 0 | 0 | 0 |  |
| Lingual g | 0 | 0 | 0 | 0 | 0 | 0 | 0 | 0 | 5.83 | 9.96 | 0 | 0.13 | 0 | 0 | 0 | 0 | 0 | 0 | 0 |  |
| Superior occipital g | 0 | 0 | 0 | 0 | 4.25 | 0 | 0 | 0 | 68.22 | 0.71 | 6.51 | 19.9 | 0.42 | 0 | 0.21 | 0 | 0 | 0 | 0 |  |
| Middle occipital g | 4.1 | 0 | 0 | 0 | 3.43 | 0 | 0 | 0 | 71.16 | 5.96 | 8.1 | 66 | 0.14 | 0 | 2.43 | 0 | 0 | 0 | 0 |  |
| Inferior occipital g | 0 | 0 | 0 | 0 | 0 | 0 | 0 | 0 | 23.86 | 18.71 | 0 | 8.9 | 0 | 0 | 0 | 0 | 0 | 0 | 0 |  |
| Fusiform g | 1.47 | 0 | 0 | 0 | 0 | 0.12 | 0.56 | 0 | 5.08 | 22.52 | 1.07 | 1.19 | 0 | 3.85 | 0 | 0 | 0.2 | 0 | 0 |  |
| Postcentral g | 7.64 | 0 | 3.48 | 0.94 | 47.58 | 22.31 | 0 | 82.5 | 25.77 | 38.29 | 10.75 | 1.36 | 2.01 | 37.82 | 6.51 | 3.24 | 0.24 | 2.85 | 0 |  |
| Superior parietal g | 0 | 0 | 0 | 0 | 41.49 | 0.14 | 0 | 48.83 | 41.09 | 11.39 | 0.05 | 8.64 | 0.68 | 0.05 | 22.37 | 0 | 0 | 0 | 0 |  |
| Inferior parietal g | 6.25 | 0 | 0 | 0 | 80.15 | 6.25 | 0.22 | 92.57 | 58.96 | 85.72 | 4.76 | 49.5 | 17.17 | 42.83 | 63.64 | 0 | 0 | 0 | 0 |  |
| Supramarginal g | 39.31 | 2.74 | 2.53 | 8.66 | 24.62 | 22.14 | 15.25 | 92.2 | 53.29 | 73.86 | 29.74 | 54.7 | 8.41 | 80.24 | 29.23 | 1.67 | 0 | 0 | 0 |  |
| Angular g | 29.11 | 8.28 | 0 | 0 | 51.83 | 3.54 | 1.37 | 29.05 | 89.67 | 62.39 | 18.44 | 94.6 | 13.3 | 0.46 | 34.02 | 0 | 0 | 0 | 0 |  |
| Precuneus | 0 | 0 | 0 | 0 | 15.16 | 1.68 | 0 | 12.25 | 53.54 | 0 | 3.83 | 1.56 | 2.7 | 0 | 0.28 | 0 | 0 | 0.12 | 0 |  |
| Paracentral lobule | 0 | 0 | 0 | 0 | 23.09 | 22.61 | 0 | 70.33 | 14.95 | 0.12 | 3.95 | 0 | 0 | 0 | 0 | 0 | 0 | 44.5 | 0 |  |
| Caudate | 51.91 | 0 | 0.1 | 4.12 | 0.4 | 22.23 | 5.03 | 0 | 0 | 28.97 | 21.93 | 0 | 8.35 | 14.89 | 0 | 1.01 | 61.37 | 0 | 0 |  |
| Putamen | 61.84 | 23.4 | 6.58 | 30.92 | 12.22 | 50.94 | 73.87 | 0.75 | 0 | 82.71 | 59.87 | 0.47 | 77.63 | 60.81 | 0 | 37.97 | 67.39 | 0 | 0 |  |
| Pallidum | 1.07 | 13.21 | 0 | 5 | 0 | 39.64 | 41.07 | 0 | 0 | 66.79 | 18.57 | 0 | 19.29 | 14.29 | 0 | 4.29 | 30.71 | 0 | 1.43 |  |
| Thalamus | 4.45 | 6.15 | 0 | 0 | 0 | 29.61 | 16.84 | 0 | 0.09 | 8.61 | 30.37 | 0 | 0.19 | 0 | 0 | 0.09 | 2.08 | 0 | 1.23 |  |
| Heschl g | 97.99 | 55.02 | 35.74 | 83.94 | 2.81 | 44.58 | 65.06 | 83.94 | 12.45 | 97.99 | 43.78 | 64.7 | 9.64 | 80.72 | 0 | 85.14 | 26.51 | 0 | 0 |  |
| Superior temporal g | 80.58 | 58.61 | 10.28 | 14.39 | 3.92 | 5.09 | 67.14 | 55.81 | 15.19 | 68.13 | 15.5 | 82.9 | 3.12 | 81.41 | 17.67 | 63.9 | 2.74 | 0 | 0 |  |
| Superior temporal pole | 52.77 | 21.75 | 6.28 | 17.79 | 6.8 | 0.15 | 58.37 | 26.91 | 0 | 17.64 | 2.84 | 2.09 | 0 | 45.52 | 9.27 | 29.45 | 0.22 | 0 | 0 |  |
| Middle temporal g | 54.21 | 2.79 | 0 | 0 | 8.69 | 5.15 | 7.08 | 25.49 | 25.49 | 47.33 | 21.52 | 66.6 | 3.67 | 17.31 | 25.47 | 34.23 | 0 | 0 | 0 |  |
| Middle temporal pole | 18.28 | 6.15 | 0 | 0 | 0 | 0 | 41.53 | 0.93 | 0 | 8.51 | 1.01 | 0 | 0 | 54.42 | 0 | 1.35 | 0 | 0 | 0 |  |
| Inferior temporal g | 3.96 | 0 | 0 | 0 | 0 | 1.04 | 2.7 | 0.31 | 2.42 | 1.69 | 7.08 | 9.81 | 0 | 5.54 | 12.85 | 0 | 0.06 | 0 | 0 |  |
| CST | 0 | 0 | 0 | 0 | 0 | 0 | 0 | 0 | 0 | 0 | 0 | 0 | 0 | 0 | 0 | 0 | 0 | 0 | 0 |  |
| ML | 0 | 0 | 0 | 0 | 0 | 0 | 0 | 0 | 0 | 0 | 0 | 0 | 0 | 0 | 0 | 0 | 0 | 0 | 0 |  |
| ICP | 0 | 0 | 0 | 0 | 0 | 0 | 0 | 0 | 0 | 0 | 0 | 0 | 0 | 0 | 0 | 0 | 0 | 0 | 0 |  |
| SCP | 0 | 0 | 0 | 0 | 0 | 0 | 0 | 0 | 0 | 0 | 0 | 0 | 0 | 0 | 0 | 0 | 0 | 0 | 0 |  |
| CP | 0 | 0 | 0 | 0 | 0 | 41.04 | 29.1 | 0 | 0 | 0.75 | 1.87 | 0 | 0 | 0 | 0 | 0 | 0 | 0 | 8.21 |  |
| IC_AL_ | 62.16 | 0 | 0 | 1.72 | 1.72 | 58.23 | 19.41 | 0 | 0 | 73.71 | 47.67 | 0 | 29.73 | 43.73 | 0 | 3.44 | 81.57 | 0 | 0 |  |
| IC_PL_ | 37.92 | 36.73 | 0 | 3.39 | 11.58 | 83.43 | 77.84 | 0 | 0 | 62.28 | 72.06 | 0 | 21.16 | 1.6 | 0 | 32.34 | 32.93 | 0 | 5.39 |  |
| IC_RL_ | 64.87 | 34.81 | 0 | 0.32 | 0 | 82.59 | 85.44 | 0 | 0.63 | 71.2 | 92.41 | 0 | 18.99 | 0 | 0 | 11.08 | 0.63 | 0 | 0 |  |
| CR_A_ | 47.9 | 0 | 31.89 | 44.04 | 72.66 | 98.01 | 3.27 | 3.27 | 0 | 52.8 | 86.1 | 0 | 18.69 | 32.71 | 0 | 36.45 | 5.96 | 0 | 0 |  |
| CR_S_ | 88.15 | 1.09 | 33.8 | 25 | 66.41 | 91.52 | 48.04 | 12.39 | 44.35 | 85 | 96.52 | 0 | 59.89 | 40.33 | 0 | 60.33 | 20.22 | 17.61 | 0 |  |
| CR_P_ | 60.4 | 10.4 | 0.22 | 10.18 | 27.21 | 50 | 47.79 | 1.55 | 76.55 | 65.27 | 85.18 | 42.7 | 58.41 | 0 | 0 | 10.4 | 5.97 | 0 | 0 |  |
| TR_P_ | 64.48 | 3.49 | 0 | 0 | 3.49 | 6.37 | 9.24 | 0 | 64.89 | 26.49 | 68.99 | 53.2 | 15.81 | 0 | 0 | 12.11 | 0 | 0 | 0 |  |
| SS | 66.08 | 0.7 | 0 | 0 | 0 | 41.96 | 56.99 | 0 | 0 | 74.13 | 66.78 | 7.69 | 0 | 0 | 0 | 0.7 | 0.7 | 0 | 0 |  |
| External capsule | 96.14 | 44.85 | 12.88 | 65.67 | 31.12 | 60.73 | 96.14 | 0.86 | 0.21 | 99.79 | 87.12 | 0.64 | 87.98 | 78.76 | 0 | 69.53 | 84.12 | 0 | 0 |  |
| CGC | 0 | 0 | 0 | 0 | 0.68 | 10.54 | 0 | 0 | 17.69 | 0 | 0 | 0 | 0 | 0 | 0 | 0 | 0 | 0 | 0 |  |
| CGH | 0 | 0 | 0 | 0 | 0 | 1.31 | 0 | 0 | 5.23 | 0 | 0 | 0 | 0 | 0 | 0 | 0 | 0 | 0 | 0 |  |
| FX/ST | 4.38 | 0 | 0 | 2.19 | 0 | 35.77 | 78.1 | 0 | 0 | 27.74 | 51.82 | 0 | 0 | 0 | 0 | 0 | 0.73 | 0 | 0 |  |
| SLF | 94.91 | 7.15 | 25.7 | 34.42 | 75.15 | 76.48 | 48.61 | 53.21 | 94.55 | 99.76 | 100 | 36.7 | 94.06 | 51.39 | 0 | 48.48 | 1.45 | 1.82 | 0 |  |
| FOs | 100 | 0 | 6.78 | 38.98 | 0 | 74.58 | 27.12 | 0 | 0 | 100 | 84.75 | 0 | 11.86 | 42.37 | 0 | 33.9 | 94.92 | 0 | 0 |  |
| FO_I_ | 73 | 30.8 | 1.52 | 31.94 | 1.14 | 57.41 | 80.23 | 4.56 | 0 | 92.02 | 78.71 | 3.8 | 67.3 | 54.75 | 0 | 42.59 | 47.91 | 0 | 0 |  |
| UNC | 100 | 14.89 | 4.26 | 55.32 | 0 | 34.04 | 80.85 | 0 | 0 | 97.87 | 74.47 | 0 | 42.55 | 100 | 0 | 44.68 | 61.7 | 0 | 0 |  |
| TAP | 30.77 | 1.28 | 0 | 0 | 0 | 14.1 | 6.41 | 0 | 84.62 | 19.23 | 30.77 | 25.6 | 35.9 | 0 | 0 | 3.85 | 0 | 0 | 0 |  |
| PCT - bilateral | 0 | 0 | 0 | 0 | 0 | 0 | 0 | 0 | 0 | 0 | 0 | 0 | 0 | 0 | 0 | 0 | 0 | 0 | 0 |  |
| BCC - bilateral | 2.08 | 0 | 0.52 | 1.1 | 1.51 | 15.11 | 0.06 | 0.17 | 4.86 | 2.03 | 10.65 | 0 | 4.69 | 0 | 0 | 0.17 | 0 | 0.98 | 0 |  |
| FX - bilateral | 0 | 0 | 0 | 0 | 0 | 0 | 0 | 0 | 0 | 0 | 0 | 0 | 0 | 0 | 0 | 0 | 0 | 0 | 0 |  |
| MCP - bilateral | 0 | 0 | 0 | 0 | 0 | 0 | 0 | 0 | 0 | 0 | 0 | 0 | 0 | 0 | 0 | 0 | 0 | 0 | 0 |  |
| GCC - bilateral | 1.33 | 0 | 0.44 | 2.21 | 2.03 | 15.56 | 0 | 0 | 0 | 1.15 | 6.37 | 0 | 0.62 | 0 | 0 | 0.35 | 0.09 | 0 | 0 |  |
| SCC - bilateral | 0.13 | 0 | 0 | 0 | 0.06 | 0.39 | 0.19 | 0 | 32.34 | 0 | 3.11 | 2.27 | 4.41 | 0 | 0 | 0 | 0 | 0 | 0 |  |

**A. Right hemisphere damage (RHD) group (cont.)**

| Areas | 1039 | 1040 | 1041 | 1042 | 1043 | 1044 | 1045 | 1046 | 1047 | 1048 | 1049 | 1050 | 1051 | 1052 | 1053 | 1054 | **Average** |
| --- | --- | --- | --- | --- | --- | --- | --- | --- | --- | --- | --- | --- | --- | --- | --- | --- | --- |
| Precentral g | 0.21 | 1.27 | 0 | 0 | 0 | 0.59 | 55.58 | 0 | 0.03 | 0 | 0 | 0 | 17.98 | 4.79 | 61.37 | 0 | **15.59** |
| Superior frontal g | 0.12 | 0 | 0 | 0 | 0 | 0 | 10.9 | 0 | 0 | 0 | 0 | 0 | 0.2 | 0.02 | 7.62 | 0 | **3.86** |
| Sup. frontal g, orbital | 0 | 0 | 0 | 0 | 0 | 0 | 2.61 | 0 | 0.2 | 0 | 0 | 0 | 2.61 | 4.41 | 21.56 | 0 | **4.14** |
| Mid. frontal g | 0.24 | 0 | 0 | 0 | 0 | 0 | 42.36 | 0 | 0.59 | 0 | 0 | 0 | 21.96 | 0.53 | 62.42 | 0 | **10.83** |
| Mid. frontal g, orbital | 0 | 0 | 0 | 0 | 0 | 0 | 3.35 | 0 | 0 | 0 | 0 | 0 | 20.1 | 0 | 52.81 | 0 | **7.92** |
| IFG_OP_ | 6.58 | 0 | 0 | 0 | 0 | 0 | 76.84 | 0 | 20.66 | 0.07 | 0 | 0 | 80.27 | 9.86 | 86.78 | 0 | **27.32** |
| IFG_TRI_ | 2.09 | 0 | 0 | 0 | 0 | 0 | 54.67 | 0 | 27.15 | 0 | 0 | 0 | 70.57 | 6.46 | 72.76 | 0 | **19.77** |
| IFG_ORB_ | 0 | 0 | 0 | 0 | 0 | 0 | 28.59 | 0 | 20.33 | 0 | 0 | 0 | 26.54 | 3.22 | 48.45 | 0 | **13.54** |
| Rolandic op. | 7.66 | 0 | 0 | 0.15 | 0.3 | 25.32 | 57.33 | 9.24 | 25.62 | 3.83 | 0 | 0 | 50.56 | 7.96 | 93.61 | 0 | **35.92** |
| Supp. motor area | 0 | 2.36 | 0 | 0 | 0 | 0 | 13.79 | 0 | 0 | 0 | 0 | 0 | 0 | 0 | 0 | 0 | **4.16** |
| Olfactory | 0 | 0 | 0 | 0 | 0 | 0 | 13.15 | 0 | 2.08 | 0 | 0 | 0 | 0.35 | 5.54 | 0 | 0 | **1.89** |
| Sup. frontal g, medial | 0 | 0 | 0 | 0 | 0 | 0 | 0 | 0 | 0 | 0 | 0 | 0 | 0 | 0.14 | 0.09 | 0 | **0.66** |
| Sup. frontal g, med. orb. | 0 | 0 | 0 | 0 | 0 | 0 | 0 | 0 | 0 | 0 | 0 | 0 | 3.39 | 2.45 | 0 | 0 | **1.30** |
| Rectus g | 0 | 0 | 0 | 0 | 0 | 0 | 0.94 | 0 | 0.13 | 0 | 0 | 0 | 0 | 6.44 | 0.67 | 0 | **0.87** |
| Insula | 13.62 | 0 | 0.34 | 4.41 | 4.29 | 0 | 95.54 | 10.51 | 88.47 | 38.25 | 0 | 0 | 65.99 | 65.37 | 93.39 | 2.88 | **42.18** |
| Anterior cingulum | 0 | 0 | 0 | 0 | 0 | 0 | 4.65 | 0 | 0 | 0 | 0 | 0 | 1.6 | 0 | 0 | 0 | **1.27** |
| Middle cingulum | 0 | 11.03 | 0 | 0 | 0 | 0.27 | 10.99 | 0 | 0 | 0 | 0 | 0 | 0 | 0 | 0.64 | 0 | **3.31** |
| Posterior cingulum | 0 | 1.19 | 0 | 0 | 0 | 0 | 0 | 0 | 0 | 0 | 0 | 0 | 0 | 0 | 0 | 0 | **0.86** |
| Hippocampus | 8.03 | 0 | 0 | 0 | 0 | 0 | 42.6 | 0 | 5.18 | 0 | 0 | 0 | 0 | 21.67 | 0 | 0 | **4.90** |
| Parahippocampal g | 0.18 | 0 | 0 | 0 | 0 | 0 | 6.54 | 0 | 0 | 0 | 0 | 0 | 0 | 0.09 | 0 | 0 | **1.01** |
| Amygdala | 1.61 | 0 | 0 | 0 | 0 | 0 | 81.45 | 0 | 38.71 | 2.82 | 0 | 0 | 0 | 17.74 | 0 | 0 | **9.15** |
| Calcarine | 4.41 | 0 | 0 | 0 | 0 | 0 | 0 | 0 | 0 | 0 | 0 | 0 | 0 | 0 | 1.99 | 0 | **1.66** |
| Cuneus | 0.28 | 0 | 0 | 0 | 0 | 0 | 0 | 0 | 0 | 0 | 0 | 0 | 0 | 0 | 2.46 | 0 | **1.73** |
| Lingual g | 3.35 | 0 | 0 | 0 | 0 | 0 | 0 | 0 | 0 | 0 | 0 | 0 | 0 | 0 | 0.04 | 0 | **0.40** |
| Superior occipital g | 0.99 | 0 | 0 | 0 | 0 | 0 | 0 | 0 | 0 | 0 | 0 | 0 | 0 | 0 | 18.26 | 0 | **2.50** |
| Middle occipital g | 20.69 | 0 | 0 | 0 | 0 | 0 | 0 | 0 | 0 | 0 | 0 | 0 | 0 | 0 | 54.29 | 0 | **5.83** |
| Inferior occipital g | 11.83 | 0 | 0 | 0 | 0 | 0 | 0 | 0 | 0 | 0 | 0 | 0 | 0 | 0 | 0 | 0 | **1.53** |
| Fusiform g | 5.24 | 0 | 0 | 0 | 0 | 0 | 0.48 | 0 | 0 | 0 | 0 | 0 | 0 | 3.85 | 0 | 0 | **1.08** |
| Postcentral g | 0.08 | 6.25 | 0 | 0 | 0 | 30.32 | 19.59 | 1.96 | 0 | 0 | 0 | 0 | 2.33 | 4.19 | 69.34 | 0 | **17.95** |
| Superior parietal g | 0 | 4.28 | 0 | 0 | 0 | 11.79 | 0 | 0 | 0 | 0 | 0 | 0 | 0 | 0 | 54.28 | 0 | **8.51** |
| Inferior parietal g | 0 | 0.52 | 0 | 0 | 0 | 33.31 | 0.82 | 0 | 0 | 0 | 0 | 0 | 0 | 0 | 93.31 | 0 | **20.12** |
| Supramarginal g | 6.33 | 0.35 | 0 | 0 | 0 | 49.54 | 1.93 | 2.28 | 0 | 0 | 0 | 0 | 0.05 | 2.99 | 96.3 | 0 | **28.08** |
| Angular g | 18.78 | 0 | 0 | 0 | 0 | 4.22 | 0 | 0 | 0 | 0 | 0 | 0 | 0 | 1.48 | 96.92 | 0 | **18.08** |
| Precuneus | 0.37 | 11.85 | 0 | 0 | 0 | 1.07 | 0.28 | 0 | 0 | 0 | 0 | 0 | 0 | 0 | 4.53 | 0 | **3.15** |
| Paracentral lobule | 0 | 18.9 | 0 | 0 | 0 | 2.03 | 36.12 | 0 | 0 | 0 | 0 | 0 | 0 | 0 | 0 | 0 | **7.94** |
| Caudate | 2.21 | 0 | 0 | 1.21 | 0 | 0 | 43.96 | 0.4 | 16.4 | 5.43 | 1.91 | 0 | 4.53 | 24.55 | 3.02 | 10.56 | **12.11** |
| Putamen | 20.39 | 0 | 0 | 6.48 | 8.93 | 0 | 85.71 | 19.92 | 81.48 | 80.08 | 10.43 | 11.47 | 44.92 | 70.21 | 31.48 | 6.58 | **35.76** |
| Pallidum | 10 | 0 | 0 | 13.21 | 2.86 | 0 | 79.64 | 19.64 | 88.57 | 48.57 | 1.07 | 3.57 | 0 | 11.07 | 0 | 0 | **18.96** |
| Thalamus | 2.84 | 0 | 0 | 6.34 | 5.2 | 0 | 31.6 | 7.19 | 5.49 | 1.99 | 0.28 | 0 | 0 | 16.46 | 0.09 | 0.85 | **4.19** |
| Heschl g | 14.06 | 0 | 0 | 2.01 | 0.4 | 0 | 42.17 | 10.04 | 15.66 | 16.87 | 0 | 0 | 9.24 | 34.14 | 98.39 | 0 | **39.74** |
| Superior temporal g | 55.43 | 0 | 0 | 0 | 0 | 5.51 | 24.67 | 2.99 | 8.47 | 0.19 | 0 | 0 | 3.09 | 8.79 | 64.79 | 0 | **29.47** |
| Superior temporal pole | 0.9 | 0 | 0 | 0 | 0 | 0 | 64.65 | 0 | 29.82 | 0 | 0 | 0 | 1.79 | 1.2 | 5.08 | 0 | **16.26** |
| Middle temporal g | 63.01 | 0 | 0 | 0 | 0 | 0 | 7.6 | 0 | 0.07 | 0 | 0 | 0 | 0 | 8.1 | 24.04 | 0 | **16.47** |
| Middle temporal pole | 1.94 | 0 | 0 | 0 | 0 | 0 | 11.88 | 0 | 0.25 | 0 | 0 | 0 | 0 | 2.36 | 0 | 0 | **9.06** |
| Inferior temporal g | 25.7 | 0 | 0 | 0 | 0 | 0 | 2.31 | 0 | 0 | 0 | 0 | 0 | 0 | 9.08 | 0 | 0 | **3.98** |
| CST | 0 | 0 | 0 | 0 | 0 | 0 | 0 | 0 | 0 | 0 | 0 | 0 | 0 | 0 | 0 | 0 | **0.00** |
| ML | 0 | 0 | 0 | 0 | 0 | 0 | 0 | 0 | 0 | 0 | 0 | 0 | 0 | 0 | 0 | 0 | **0.00** |
| ICP | 0 | 0 | 0 | 0 | 0 | 0 | 0 | 0 | 0 | 0 | 0 | 0 | 0 | 0 | 0 | 0 | **0.00** |
| SCP | 0 | 0 | 0 | 0 | 0 | 0 | 0 | 0 | 0 | 0 | 0 | 0 | 0 | 0 | 0 | 0 | **0.00** |
| CP | 0 | 0 | 0 | 0 | 0 | 0 | 60.07 | 2.99 | 0.37 | 0 | 0 | 0 | 0 | 4.48 | 0 | 0 | **3.08** |
| IC_AL_ | 2.7 | 0 | 0 | 0.25 | 3.19 | 0 | 71.01 | 0 | 51.35 | 31.94 | 16.71 | 0 | 4.42 | 26.54 | 1.23 | 27.76 | **22.79** |
| IC_PL_ | 34.33 | 0 | 0 | 56.29 | 45.51 | 0 | 90.82 | 44.31 | 48.5 | 52.3 | 17.56 | 0.2 | 0 | 51.9 | 1.8 | 31.34 | **27.13** |
| IC_RL_ | 52.53 | 0 | 0 | 11.71 | 3.16 | 0 | 74.68 | 27.53 | 4.43 | 31.01 | 0 | 0 | 0 | 83.23 | 47.78 | 2.53 | **23.13** |
| CR_A_ | 23.71 | 0 | 0 | 0 | 0 | 0 | 67.52 | 0 | 27.45 | 3.62 | 0.82 | 0 | 60.63 | 54.67 | 65.3 | 0 | **24.25** |
| CR_S_ | 34.35 | 3.26 | 3.15 | 11.85 | 1.3 | 0 | 96.09 | 34.02 | 37.5 | 23.26 | 4.13 | 0 | 3.59 | 54.02 | 75.54 | 23.91 | **37.21** |
| CR_P_ | 26.99 | 47.57 | 0 | 1.77 | 0 | 0 | 46.24 | 20.58 | 0.44 | 2.88 | 0 | 0 | 0 | 27.65 | 62.83 | 6.64 | **24.36** |
| TR_P_ | 66.53 | 0 | 0 | 0 | 0 | 0 | 2.05 | 0 | 0 | 0 | 0 | 0 | 0 | 15.81 | 28.54 | 0 | **11.11** |
| SS | 40.21 | 0 | 0 | 0 | 17.17 | 0 | 47.9 | 0 | 5.24 | 1.4 | 0 | 0 | 0 | 52.8 | 1.4 | 0 | **13.10** |
| External capsule | 42.49 | 0 | 1.5 | 20.17 | 0 | 0 | 97.42 | 40.77 | 89.06 | 97.64 | 3.65 | 5.79 | 66.31 | 98.5 | 76.82 | 25.75 | **48.61** |
| CGC | 0 | 0 | 0 | 0 | 0 | 0 | 14.29 | 0 | 0 | 0 | 0 | 0 | 0 | 0 | 0 | 0 | **1.57** |
| CGH | 0 | 0 | 0 | 0 | 0 | 0 | 4.58 | 0 | 0 | 0 | 0 | 0 | 0 | 0 | 0 | 0 | **0.21** |
| FX/ST | 11.68 | 0 | 0 | 0 | 0 | 0 | 51.82 | 0 | 9.49 | 1.46 | 0 | 0 | 0 | 42.34 | 0 | 0 | **7.27** |
| SLF | 39.03 | 5.94 | 1.21 | 0.73 | 0 | 45.94 | 56.12 | 18.06 | 15.64 | 8.12 | 0 | 0 | 10.06 | 77.94 | 93.94 | 0.12 | **43.04** |
| FOs | 6.78 | 0 | 3.39 | 1.69 | 0 | 0 | 100 | 1.69 | 55.93 | 55.93 | 0 | 0 | 0 | 100 | 8.47 | 47.46 | **31.01** |
| FO_I_ | 25.1 | 0 | 0 | 0 | 0 | 0 | 92.4 | 1.52 | 74.14 | 62.36 | 0 | 0 | 39.92 | 84.41 | 31.94 | 0 | **33.01** |
| UNC | 10.64 | 0 | 0 | 0 | 0 | 0 | 100 | 0 | 100 | 19.15 | 0 | 0 | 12.77 | 91.49 | 34.04 | 0 | **31.36** |
| TAP | 2.56 | 2.56 | 0 | 0 | 0 | 0 | 0 | 0 | 0 | 0 | 0 | 0 | 0 | 0 | 15.38 | 0 | **6.39** |
| PCT - bilateral | 0 | 0 | 0 | 0 | 0 | 0 | 0 | 0 | 0 | 0 | 0 | 0 | 0 | 0 | 0 | 0 | **0.00** |
| BCC - bilateral | 1.16 | 1.68 | 0 | 0 | 0 | 0 | 10.31 | 0.52 | 0 | 0 | 0 | 0 | 0 | 0.52 | 0.58 | 0 | **1.80** |
| FX - bilateral | 0 | 0 | 0 | 0 | 0 | 0 | 0 | 0 | 0 | 0 | 0 | 0 | 0 | 0 | 0 | 0 | **0.00** |
| MCP - bilateral | 0 | 0 | 0 | 0 | 0 | 0 | 0 | 0 | 0 | 0 | 0 | 0 | 0 | 0 | 0 | 0 | **0.00** |
| GCC - bilateral | 1.33 | 0 | 0 | 0 | 0 | 0 | 11.49 | 0 | 0 | 0 | 0 | 0 | 1.41 | 0.8 | 0.71 | 0 | **2.08** |
| SCC - bilateral | 0.32 | 5.96 | 0 | 0 | 0 | 0 | 0 | 0 | 0 | 0 | 0 | 0 | 0 | 0 | 0.84 | 0 | **1.09** |

**B. Left hemisphere damage (LHD) group**

| Area | 2001 | 2002 | 2003 | 2004 | 2005 | 2006 | 2007 | 2008 | 2009 | 2010 | 2011 | 2012 | 2013 | 2014 | 2015 | 2016 | 2017 | 2018 | 2019 |
| --- | --- | --- | --- | --- | --- | --- | --- | --- | --- | --- | --- | --- | --- | --- | --- | --- | --- | --- | --- |
| Precentral g | 25.84 | 7.88 | 0 | 7.2 | 61.32 | 0 | 0 | 35.42 | 6.01 | 0 | 0 | 0 | 0 | 0 | 0.2 | 0.34 | 0 | 0 | 0 |
| Superior frontal g | 14.39 | 16.95 | 0 | 0 | 37.45 | 0 | 0 | 0.03 | 0.56 | 0 | 0 | 0 | 0 | 0 | 27.9 | 0 | 0 | 0 | 0 |
| Sup. frontal g, orbital | 0 | 0 | 0 | 0 | 0 | 0 | 0 | 0 | 0 | 0 | 0 | 0 | 0 | 0 | 12.77 | 0 | 0 | 0 | 0 |
| Mid. frontal g | 4.19 | 14.7 | 0 | 0 | 20.15 | 0 | 0 | 0.8 | 4.46 | 0 | 0 | 0 | 0 | 0 | 1.11 | 0.41 | 0 | 0 | 0 |
| Mid. frontal g, orbital | 0 | 0 | 0 | 0 | 0 | 0 | 0 | 0 | 0 | 0 | 0 | 0 | 0 | 0 | 0 | 0.11 | 0 | 0 | 0 |
| IFG_OP_ | 0 | 12.24 | 0 | 0 | 10.98 | 0 | 0 | 2.99 | 83.72 | 0 | 0 | 0 | 0 | 0 | 0 | 10.4 | 0 | 0 | 0 |
| IFG_TRI_ | 0 | 12.3 | 0 | 0 | 1.19 | 0 | 0 | 2.85 | 50.49 | 0 | 0 | 0 | 0 | 0 | 0 | 17.04 | 0 | 0 | 0 |
| IFG_ORB_ | 0 | 0 | 0 | 0 | 0 | 0 | 0 | 0 | 23.31 | 0 | 0 | 0 | 0 | 0 | 1.42 | 12.43 | 0 | 0 | 0 |
| Rolandic op. | 0 | 1.31 | 3.54 | 0 | 4.44 | 0 | 29.6 | 46.67 | 98.59 | 2.42 | 4.04 | 0 | 25.35 | 0 | 0 | 4.95 | 0 | 0 | 0 |
| Supp. motor area | 32.56 | 8.85 | 0 | 0.19 | 66 | 0 | 0 | 0 | 0 | 0 | 0 | 0 | 0 | 0 | 8.94 | 0 | 0 | 0 | 0 |
| Olfactory | 0 | 0 | 0 | 0 | 0 | 0 | 0 | 0 | 0.36 | 0 | 0 | 0 | 0 | 0 | 19.64 | 0.71 | 0 | 1.79 | 0 |
| Sup. frontal g, medial | 0.03 | 1.54 | 0 | 0 | 14.87 | 0 | 0 | 0 | 0.07 | 0 | 0 | 0 | 0 | 0 | 40.88 | 0 | 0 | 0 | 0 |
| Sup. frontal g, med. orb. | 0 | 0 | 0 | 0 | 0 | 0 | 0 | 0 | 0 | 0 | 0 | 0 | 0 | 0 | 0.83 | 0 | 0 | 0 | 0 |
| Rectus g | 0 | 0 | 0 | 0 | 0 | 0 | 0 | 0 | 0 | 0 | 0 | 0 | 0 | 0 | 24.77 | 0 | 0 | 0 | 0 |
| Insula | 0.05 | 9.53 | 0.11 | 0 | 2.15 | 0 | 6.84 | 4.14 | 92.09 | 0.22 | 3.55 | 0 | 2.1 | 0 | 2.05 | 48.17 | 0 | 14.75 | 0 |
| Anterior cingulum | 0 | 19 | 0 | 0 | 8.86 | 0 | 0 | 0 | 0 | 0 | 0 | 0 | 0 | 0 | 31.93 | 0 | 0 | 0 | 0 |
| Middle cingulum | 23.18 | 16.13 | 2.01 | 0.15 | 35.91 | 0 | 0 | 0 | 0.26 | 0 | 0 | 0 | 0 | 0 | 12.16 | 0 | 0 | 0 | 0 |
| Posterior cingulum | 0 | 0 | 0.86 | 0 | 1.73 | 0 | 0 | 0 | 0 | 0 | 0 | 0 | 0 | 0 | 0 | 0 | 0 | 0 | 0 |
| Hippocampus | 0 | 0 | 0 | 0 | 0 | 0 | 0 | 0 | 0.11 | 21.03 | 0 | 0 | 0 | 16.09 | 0 | 0.97 | 20.28 | 0.21 | 1.29 |
| Parahippocampal g | 0 | 0 | 0 | 0 | 0 | 0 | 0 | 0 | 0 | 2.56 | 0 | 0 | 0 | 33.64 | 0 | 1.23 | 22.29 | 0 | 0.41 |
| Amygdala | 0 | 0 | 0 | 0 | 0 | 0 | 0 | 0 | 4.09 | 0 | 0 | 0 | 0 | 0 | 0 | 20.45 | 0 | 15.45 | 0 |
| Calcarine | 0 | 0 | 6.82 | 0 | 0.09 | 0 | 0 | 0 | 0 | 0.58 | 1.68 | 0 | 0 | 38.49 | 0 | 0 | 33.88 | 0 | 0 |
| Cuneus | 0 | 0 | 9.04 | 0 | 6.75 | 0 | 0 | 0 | 0 | 0.79 | 0 | 0 | 0 | 23.79 | 0 | 0 | 12.32 | 0 | 0 |
| Lingual g | 0 | 0 | 0 | 0 | 0 | 0 | 0 | 0 | 0 | 0.43 | 0 | 0 | 0 | 72.17 | 0 | 0 | 53.7 | 0 | 0 |
| Superior occipital g | 0.22 | 0 | 32.21 | 0 | 23.06 | 0 | 0 | 0 | 0 | 0.66 | 0.81 | 0 | 0 | 24.16 | 0 | 0 | 10.91 | 0 | 0 |
| Middle occipital g | 0.67 | 0 | 38.26 | 0 | 15.96 | 3.09 | 0 | 0 | 0 | 4.25 | 0.76 | 0.43 | 1.44 | 8.56 | 0 | 0 | 7.77 | 0 | 0 |
| Inferior occipital g | 0 | 0 | 0 | 0 | 0 | 0 | 0 | 0 | 0 | 2.02 | 0 | 0 | 0 | 50.16 | 0 | 0 | 45.27 | 0 | 0 |
| Fusiform g | 0 | 0 | 0 | 0 | 0 | 0 | 0 | 0 | 0 | 0.22 | 0 | 0 | 0 | 59.13 | 0 | 0 | 38.35 | 0 | 3.81 |
| Postcentral g | 28.37 | 5.16 | 0 | 2.67 | 62.64 | 0 | 3.83 | 28.06 | 10.07 | 0.03 | 2 | 12.82 | 10.41 | 0 | 0 | 0 | 0 | 0 | 0 |
| Superior parietal g | 26.44 | 0.15 | 43.44 | 0 | 26.92 | 0 | 0 | 0 | 0 | 0 | 0 | 15.11 | 28.67 | 0 | 0 | 0 | 0 | 0 | 0 |
| Inferior parietal g | 12.46 | 1.59 | 53.13 | 0 | 47.4 | 8.87 | 0.53 | 0 | 0 | 0 | 4.33 | 64.73 | 80.34 | 0 | 0 | 0 | 0 | 0 | 0 |
| Supramarginal g | 0 | 0 | 20.94 | 0 | 50.56 | 12.1 | 36.94 | 6.21 | 11.23 | 22.13 | 16 | 25.16 | 92.91 | 0 | 0 | 0 | 0 | 0 | 0 |
| Angular g | 0.51 | 0 | 74.08 | 0 | 19.52 | 34.27 | 0 | 0 | 0 | 21.48 | 1.62 | 5.63 | 65.64 | 0 | 0 | 0 | 0 | 0 | 0 |
| Precuneus | 21.83 | 3.03 | 19.42 | 0.54 | 19.84 | 0 | 0 | 0 | 0 | 0.2 | 0.06 | 0.03 | 0 | 0 | 0 | 0 | 2.13 | 0 | 0 |
| Paracentral lobule | 66.64 | 10.67 | 0 | 25.95 | 54.19 | 0 | 0 | 0 | 0 | 0 | 0 | 0 | 0 | 0 | 0 | 0 | 0 | 0 | 0 |
| Caudate | 0.73 | 0 | 0 | 0 | 13.83 | 0 | 0 | 0 | 11.54 | 0 | 0 | 0 | 0 | 0 | 4.26 | 8.94 | 0 | 29.11 | 0 |
| Putamen | 0 | 3.27 | 0 | 0 | 0 | 0 | 0 | 0 | 59.96 | 0.2 | 0.3 | 0 | 0 | 0 | 15.06 | 68.58 | 0 | 95.94 | 0 |
| Pallidum | 0 | 0 | 0 | 0 | 0 | 0 | 0 | 0 | 4.44 | 0 | 0 | 0 | 0 | 0 | 0 | 30.38 | 0 | 67.58 | 5.12 |
| Thalamus | 0 | 0 | 0 | 0 | 0 | 0 | 0 | 0 | 0 | 0.18 | 0 | 0 | 0 | 0 | 0 | 1.64 | 0 | 7.91 | 11.18 |
| Heschl g | 0 | 0 | 0 | 0 | 0 | 0 | 24.44 | 24 | 96.89 | 0.89 | 7.56 | 0 | 50.22 | 0 | 0 | 1.78 | 0 | 8 | 0 |
| Superior temporal g | 0 | 0 | 8.62 | 0 | 0 | 0 | 12.37 | 5.36 | 38.5 | 13.55 | 7.23 | 0 | 43.9 | 0 | 0 | 0.09 | 0 | 3.7 | 0 |
| Superior temporal pole | 0 | 0 | 0 | 0 | 0 | 0 | 0 | 0 | 19.46 | 0 | 0 | 0 | 0 | 0 | 0 | 1.09 | 0 | 3.74 | 0 |
| Middle temporal g | 0 | 0 | 5.32 | 0 | 1.86 | 0 | 0 | 0 | 0.22 | 30.35 | 5.26 | 0 | 1.78 | 0 | 0 | 0 | 0 | 0.3 | 0 |
| Middle temporal pole | 0 | 0 | 0 | 0 | 0 | 0 | 0 | 0 | 0 | 0 | 0 | 0 | 0 | 0 | 0 | 0 | 0 | 0 | 0 |
| Inferior temporal g | 0 | 0 | 0 | 0 | 0 | 0 | 0 | 0 | 0 | 0.38 | 0 | 0 | 0 | 9.03 | 0 | 0 | 0.12 | 0 | 0.06 |
| CST | 0 | 0 | 0 | 0 | 0 | 0 | 0 | 0 | 0 | 0 | 0 | 0 | 0 | 0 | 0 | 0 | 0 | 0 | 0 |
| ML | 0 | 0 | 0 | 0 | 0 | 0 | 0 | 0 | 0 | 0 | 0 | 0 | 0 | 0 | 0 | 0 | 0 | 0 | 0 |
| ICP | 0 | 0 | 0 | 0 | 0 | 0 | 0 | 0 | 0 | 0 | 0 | 0 | 0 | 0 | 0 | 0 | 0 | 0 | 0 |
| SCP | 0 | 0 | 0 | 0 | 0 | 0 | 0 | 0 | 0 | 0 | 0 | 0 | 0 | 0 | 0 | 0 | 0 | 0 | 0 |
| CP | 0 | 0 | 0 | 0 | 0 | 0 | 0 | 0 | 0 | 4.18 | 0 | 0 | 0 | 0 | 0 | 0 | 0.38 | 0.38 | 9.13 |
| IC_AL_ | 0 | 0 | 0 | 0 | 0 | 0 | 0 | 0 | 44.13 | 0 | 0 | 0 | 0 | 0 | 8.93 | 50 | 0 | 83.16 | 0 |
| IC_PL_ | 2.31 | 0 | 0 | 0 | 0 | 0 | 2.31 | 0 | 35.64 | 0 | 11.74 | 0 | 0 | 0 | 0 | 50.1 | 0 | 68.13 | 10.06 |
| IC_RL_ | 0.32 | 0 | 3.86 | 0 | 0 | 0 | 3.22 | 0 | 14.15 | 52.73 | 28.94 | 0 | 2.25 | 0 | 0 | 15.76 | 0 | 37.3 | 0.32 |
| CR_A_ | 0 | 47.28 | 0 | 0 | 17.92 | 0 | 0 | 0 | 29.02 | 0 | 0 | 0 | 0 | 0 | 42.08 | 44.51 | 0 | 3.93 | 0 |
| CR_S_ | 41.77 | 50.97 | 0 | 0 | 97.51 | 2.06 | 2.92 | 7.14 | 38.96 | 0 | 6.17 | 0 | 0 | 0 | 13.53 | 39.61 | 0 | 24.35 | 0 |
| CR_P_ | 20.4 | 17.04 | 51.79 | 0 | 85.2 | 32.29 | 12.33 | 0 | 7.62 | 53.36 | 29.82 | 2.02 | 6.28 | 0 | 0 | 1.79 | 0 | 6.28 | 0 |
| TR_P_ | 0 | 0 | 37.66 | 0 | 7.11 | 0 | 0 | 0 | 0 | 70.92 | 35.98 | 0 | 1.05 | 0.42 | 0 | 0 | 13.18 | 0 | 0 |
| SS | 0 | 0 | 0 | 0 | 0 | 0 | 0 | 0 | 0 | 63.89 | 0 | 0 | 0 | 6.94 | 0 | 0 | 15.28 | 2.08 | 0 |
| External capsule | 1.33 | 10.67 | 0 | 0 | 0 | 0 | 1.33 | 0.22 | 94.22 | 0 | 6 | 0 | 0 | 0 | 4.89 | 94.89 | 0 | 90 | 0 |
| CGC | 1.78 | 51.34 | 0 | 0 | 30.56 | 0 | 0 | 0 | 0 | 0 | 0 | 0 | 0 | 0 | 16.32 | 0 | 0 | 0 | 0 |
| CGH | 0 | 0 | 0 | 0 | 0 | 0 | 0 | 0 | 0 | 2.29 | 0 | 0 | 0 | 44.27 | 0 | 0 | 49.62 | 0 | 0 |
| FX/ST | 0 | 0 | 0 | 0 | 0 | 0 | 0 | 0 | 0 | 59.86 | 0 | 0 | 0 | 2.04 | 0 | 0.68 | 10.88 | 6.8 | 0 |
| SLF | 36.44 | 15.09 | 20.49 | 0 | 79.39 | 13.87 | 23.44 | 23.07 | 25.15 | 37.3 | 45.64 | 9.82 | 20.98 | 0 | 0 | 12.02 | 0 | 0.74 | 0 |
| FOs | 0 | 0 | 0 | 0 | 100 | 0 | 0 | 0 | 87.27 | 0 | 0 | 0 | 0 | 0 | 0 | 67.27 | 0 | 69.09 | 0 |
| FO_I_ | 0 | 0 | 0 | 0 | 0 | 0 | 0 | 0 | 52.07 | 2.07 | 0 | 0 | 0 | 0 | 30.17 | 54.96 | 0 | 92.15 | 0 |
| UNC | 0 | 0 | 0 | 0 | 0 | 0 | 0 | 0 | 55.1 | 0 | 0 | 0 | 0 | 0 | 0 | 36.73 | 0 | 87.76 | 0 |
| TAP | 0 | 0 | 83.1 | 0 | 0 | 0 | 0 | 0 | 0 | 91.55 | 1.41 | 0 | 0 | 0 | 0 | 0 | 2.82 | 0 | 0 |
| PCT- bilateral | 0 | 0 | 0 | 0 | 0 | 0 | 0 | 0 | 0 | 0 | 0 | 0 | 0 | 0 | 0 | 0 | 0 | 0 | 0 |
| BCC- bilateral | 1.51 | 11.81 | 0 | 0 | 25.19 | 0 | 0 | 0 | 0 | 0 | 0 | 0 | 0 | 0 | 6.43 | 0.81 | 0 | 0 | 0 |
| FX- bilateral | 0 | 0 | 0 | 0 | 0 | 0 | 0 | 0 | 0 | 0 | 0 | 0 | 0 | 0 | 0 | 0 | 0 | 0 | 0 |
| MCP- bilateral | 0 | 0 | 0 | 0 | 0 | 0 | 0 | 0 | 0 | 0 | 0 | 0 | 0 | 0 | 0 | 0 | 0 | 0 | 0 |
| GCC- bilateral | 0 | 18.3 | 0 | 0 | 2.48 | 0 | 0 | 0 | 0 | 0 | 0 | 0 | 0 | 0 | 16.53 | 0.62 | 0 | 0.35 | 0 |
| SCC- bilateral | 0 | 0.32 | 9.66 | 0 | 7.71 | 0.45 | 0 | 0 | 0 | 5.06 | 0.71 | 0 | 0 | 0.19 | 0 | 0 | 3.63 | 0 | 0 |

**B. Left hemisphere damage (LHD) group (cont.)**

| Areas | 2020 | 2021 | 2022 | 2023 | 2024 | 2025 | 2026 | 2027 | 2028 | 2029 | 2030 | 2031 | 2032 | 2033 | 2033 | 2035 | 2036 |
| --- | --- | --- | --- | --- | --- | --- | --- | --- | --- | --- | --- | --- | --- | --- | --- | --- | --- |
| Precentral g | 0 | 55.16 | 0 | 0 | 7.23 | 0 | 0 | 0 | 0 | 0.09 | 0 | 2.5 | 2.3 | 0 | 0 | 0 | 4.88 |
| Superior frontal g | 0.69 | 3.08 | 0 | 0 | 0 | 0 | 0 | 0 | 0 | 0 | 0 | 0 | 0 | 0 | 0 | 0 | 0 |
| Sup. frontal g, orbital | 0 | 0 | 0 | 0 | 0.52 | 0 | 0 | 0 | 0 | 0 | 0 | 0.83 | 2.6 | 0 | 0 | 0 | 0 |
| Mid. frontal g | 2.12 | 36.79 | 0 | 0 | 0.41 | 0 | 0 | 0 | 0 | 0.02 | 0 | 2.76 | 0.12 | 0 | 0 | 0 | 0 |
| Mid. frontal g, orbital | 0 | 0 | 0 | 0 | 0 | 0 | 0 | 0 | 0 | 0 | 0 | 0 | 0 | 0 | 0 | 0 | 0 |
| IFG_OP_ | 1.73 | 95.57 | 0 | 0 | 51.06 | 0 | 0 | 0 | 0 | 0 | 0 | 5.49 | 4.05 | 0 | 0 | 0 | 0 |
| IFG_TRI_ | 2.25 | 79.72 | 0 | 0 | 31.47 | 0 | 0 | 0 | 0 | 0 | 0 | 2.37 | 2.61 | 0 | 0 | 0 | 0 |
| IFG_ORB_ | 0 | 0.47 | 0 | 0 | 14.14 | 0 | 0 | 0 | 0 | 0 | 0.3 | 1.72 | 2.31 | 0 | 0 | 0 | 0 |
| Rolandic op. | 0 | 42.63 | 0 | 2.22 | 41.82 | 0 | 1.31 | 14.04 | 0 | 0 | 0 | 0 | 26.36 | 0 | 0 | 0 | 0 |
| Supp. motor area | 0 | 0 | 0 | 0 | 0 | 0 | 0 | 0 | 0 | 0 | 0 | 0 | 0 | 0 | 0 | 0 | 1.07 |
| Olfactory | 0 | 0 | 0 | 0 | 2.86 | 0 | 0 | 0 | 0 | 0 | 0.71 | 0 | 3.57 | 0 | 0 | 0 | 0 |
| Sup. frontal g, medial | 0.07 | 0 | 0 | 0 | 0 | 0 | 0 | 0 | 0 | 0 | 0 | 0 | 0 | 0 | 0 | 0 | 0 |
| Sup. frontal g, med. orb. | 0 | 0 | 0 | 0 | 0 | 0 | 0 | 0 | 0 | 0 | 0 | 0 | 0 | 0 | 0 | 0 | 0 |
| Rectus g | 0 | 0 | 0 | 0 | 0 | 0 | 0 | 0 | 0 | 0 | 0 | 1.41 | 1.53 | 0 | 0 | 0 | 0 |
| Insula | 0.54 | 27.13 | 0 | 8.56 | 79.17 | 0.97 | 6.4 | 25.03 | 0.05 | 0 | 5.71 | 14.64 | 84.34 | 0 | 0.27 | 0 | 0 |
| Anterior cingulum | 0 | 0 | 0 | 0 | 0 | 0 | 0 | 0 | 0 | 0 | 0 | 0 | 0 | 0 | 0 | 0 | 0 |
| Middle cingulum | 4.48 | 0.46 | 0 | 0 | 0 | 0 | 0 | 0 | 0 | 0 | 0 | 0 | 0 | 0 | 0 | 0 | 9.84 |
| Posterior cingulum | 73.43 | 0 | 0 | 0 | 0 | 0 | 0 | 0 | 0 | 0 | 0 | 0 | 0 | 0 | 0 | 0 | 0 |
| Hippocampus | 91.09 | 0 | 0 | 0.64 | 6.87 | 0 | 0 | 0.32 | 0 | 0 | 2.04 | 0 | 22.75 | 0 | 0 | 8.58 | 0 |
| Parahippocampal g | 66.77 | 0 | 0 | 0 | 0 | 0 | 0 | 0 | 0 | 0 | 0 | 0 | 0.1 | 0 | 0 | 19.63 | 0 |
| Amygdala | 23.18 | 0 | 0 | 0 | 26.82 | 0 | 0 | 0 | 0 | 0 | 9.09 | 0 | 9.09 | 0 | 0 | 0 | 0 |
| Calcarine | 88.62 | 0 | 0 | 0 | 0 | 0 | 0 | 0 | 0 | 0 | 0 | 0 | 0 | 0 | 0 | 37.95 | 0 |
| Cuneus | 60.42 | 0 | 0 | 0 | 0 | 0 | 0 | 0 | 0 | 0 | 0 | 0 | 0 | 0 | 0 | 0.13 | 0 |
| Lingual g | 96.32 | 0 | 0 | 0 | 0 | 0 | 0 | 0 | 0 | 0 | 0 | 0 | 0 | 0 | 0 | 74.37 | 0 |
| Superior occipital g | 12.81 | 0 | 0 | 0 | 0 | 0 | 0 | 0 | 0 | 0 | 0 | 0 | 0 | 0 | 0 | 1.68 | 0.59 |
| Middle occipital g | 5.99 | 0 | 0 | 0 | 0 | 0 | 0 | 0 | 0 | 0 | 0 | 0 | 0 | 0 | 0 | 1.8 | 0.89 |
| Inferior occipital g | 72.79 | 0 | 0 | 0 | 0 | 0 | 0 | 0 | 0 | 0 | 0 | 0 | 0 | 0 | 0 | 19.98 | 0 |
| Fusiform g | 87.1 | 0 | 0 | 0 | 1.34 | 0 | 0 | 0 | 0 | 0 | 0 | 0 | 2.94 | 0 | 0 | 14.2 | 0 |
| Postcentral g | 0 | 17.21 | 0 | 0 | 2.8 | 0 | 0 | 0.08 | 0 | 0 | 0 | 0.08 | 1.1 | 0 | 0 | 0 | 20.4 |
| Superior parietal g | 3 | 0 | 0 | 0 | 0 | 0 | 0 | 0 | 0 | 0 | 0 | 0 | 0 | 0 | 0 | 0 | 42.03 |
| Inferior parietal g | 0 | 0 | 0 | 0 | 0 | 0 | 0 | 0 | 0 | 0 | 0 | 0 | 0.37 | 0 | 0 | 0 | 23.33 |
| Supramarginal g | 0 | 0 | 0 | 0 | 0.08 | 0 | 0 | 12.82 | 0 | 0 | 0 | 0 | 19.59 | 0 | 0 | 0 | 6.61 |
| Angular g | 0 | 0 | 0 | 0 | 0 | 0 | 0 | 4.01 | 0 | 0 | 0 | 0 | 0.68 | 0 | 0 | 0 | 4.01 |
| Precuneus | 41.52 | 0 | 0 | 0 | 0 | 0 | 0 | 0 | 0 | 0 | 0 | 0 | 0 | 0 | 0 | 1.59 | 16.07 |
| Paracentral lobule | 0 | 0 | 0 | 0 | 0 | 0 | 0 | 0 | 0 | 0 | 0 | 0 | 0 | 0 | 0 | 0 | 16.75 |
| Caudate | 0.83 | 1.25 | 5.61 | 2.18 | 44.49 | 39.19 | 0.21 | 0.52 | 6.24 | 0 | 0.31 | 10.5 | 12.27 | 0 | 0 | 0 | 0 |
| Putamen | 3.37 | 2.48 | 12.19 | 29.83 | 66.8 | 38.85 | 22.4 | 4.56 | 13.97 | 0 | 76.71 | 48.86 | 70.27 | 0 | 1.29 | 0 | 0 |
| Pallidum | 0.68 | 0 | 1.02 | 11.95 | 27.99 | 17.41 | 3.07 | 0 | 0.34 | 0 | 28.33 | 6.48 | 27.3 | 0 | 3.41 | 0 | 0 |
| Thalamus | 26.55 | 0 | 2.64 | 0.09 | 0 | 2.09 | 3.55 | 0 | 0.64 | 0 | 0.27 | 8.09 | 17 | 0 | 21.64 | 0 | 0 |
| Heschl g | 0 | 0 | 0 | 32 | 4.89 | 0 | 4.44 | 20.44 | 0 | 0 | 0 | 4.44 | 64.89 | 0 | 0 | 0 | 0 |
| Superior temporal g | 0.26 | 3.22 | 0 | 16.38 | 14.46 | 0 | 0 | 20.17 | 0 | 0 | 0 | 0.17 | 12.2 | 0 | 0 | 0 | 0 |
| Superior temporal pole | 0 | 4.82 | 0 | 1.87 | 28.87 | 0 | 0 | 0.62 | 0 | 0 | 0 | 0 | 2.8 | 0 | 0 | 0 | 0 |
| Middle temporal g | 3.68 | 0 | 0 | 2.79 | 18.23 | 0 | 0 | 1.72 | 0 | 0 | 0 | 0 | 4.98 | 0 | 0 | 0 | 0 |
| Middle temporal pole | 0 | 0 | 0 | 0 | 2.78 | 0 | 0 | 0 | 0 | 0 | 0 | 0 | 0.66 | 0 | 0 | 0 | 0 |
| Inferior temporal g | 36.22 | 0 | 0 | 0 | 1.78 | 0 | 0 | 0 | 0 | 0 | 0 | 0 | 20.94 | 0 | 0 | 0.44 | 0 |
| CST | 0 | 0 | 0 | 0 | 0 | 0 | 0 | 0 | 0 | 0 | 0 | 0 | 0 | 12.36 | 0 | 0 | 0 |
| ML | 0 | 0 | 0 | 0 | 0 | 0 | 0 | 0 | 0 | 0 | 0 | 0 | 0 | 3.61 | 0 | 0 | 0 |
| ICP | 0 | 0 | 0 | 0 | 0 | 0 | 0 | 0 | 0 | 0 | 0 | 0 | 0 | 0 | 0 | 0 | 0 |
| SCP | 0 | 0 | 0 | 0 | 0 | 0 | 0 | 0 | 0 | 0 | 0 | 0 | 0 | 0 | 0 | 0 | 0 |
| CP | 7.98 | 0 | 0 | 0 | 0 | 0 | 3.42 | 0 | 0 | 0 | 0 | 2.66 | 2.66 | 0 | 12.55 | 0 | 0 |
| IC_AL_ | 5.1 | 11.99 | 0 | 0 | 44.64 | 46.68 | 7.14 | 2.55 | 3.06 | 0 | 27.3 | 40.82 | 40.82 | 0 | 0 | 0 | 0 |
| IC_PL_ | 36.48 | 0.21 | 38.57 | 24.53 | 0 | 34.8 | 51.36 | 3.77 | 35.85 | 0 | 30.19 | 57.44 | 71.91 | 0.42 | 53.04 | 0 | 0 |
| IC_RL_ | 69.13 | 0 | 26.37 | 34.08 | 9.32 | 0.64 | 25.08 | 24.12 | 2.25 | 0 | 8.68 | 42.77 | 92,28 | 0 | 7.07 | 0 | 0 |
| CR_A_ | 29.13 | 24.05 | 0 | 0 | 37.23 | 0 | 0.35 | 0.23 | 0 | 0 | 2.66 | 27.51 | 40.92 | 0 | 0 | 0 | 0 |
| CR_S_ | 15.x91 | 47.19 | 12.88 | 20.35 | 47.4 | 11.26 | 38.1 | 14.5 | 4.98 | 4 | 14.07 | 59.9 | 53.35 | 0 | 0 | 0 | 26.52 |
| CR_P_ | 15.02 | 0 | 8.07 | 15.92 | 12.78 | 0.22 | 9.87 | 36.55 | 2.69 | 0 | 4.93 | 8.7 | 45.29 | 0 | 0 | 0 | 65.02 |
| TR_P_ | 89.12 | 0 | 0 | 0 | 7.53 | 0 | 0 | 4.81 | 0 | 0 | 0 | 0 | 10.04 | 0 | 0 | 6.9 | 0 |
| SS | 100 | 0 | 0 | 14.24 | 28.82 | 0 | 0 | 4.17 | 0 | 0 | 1.04 | 3.82 | 90.28 | 0 | 0 | 15.28 | 0 |
| External capsule | 4.22 | 19.11 | 8.22 | 39.11 | 55.56 | 36.67 | 47.33 | 22.44 | 8.89 | 0 | 88.22 | 82.67 | 100 | 0 | 5.11 | 0 | 0 |
| CGC | 13.06 | 0 | 0 | 0 | 0 | 0 | 0 | 0 | 0 | 0 | 0 | 0 | 0 | 0 | 0 | 0 | 2.37 |
| CGH | 100 | 0 | 0 | 0 | 0 | 0 | 0 | 0 | 0 | 0 | 0 | 0 | 0 | 0 | 0 | 19.85 | 0 |
| FX/ST | 100 | 0 | 0 | 0.68 | 12.24 | 0 | 0 | 0.68 | 0 | 0 | 6.8 | 0 | 54.42 | 0 | 0 | 0 | 0 |
| SLF | 1.6 | 37.42 | 0 | 0.98 | 28.83 | 0 | 10.43 | 39.14 | 0 | 0 | 0 | 26.13 | 71.66 | 0 | 0 | 0 | 50.55 |
| FOs | 10.91 | 58.18 | 5.45 | 0 | 81.82 | 58.18 | 25.45 | 0 | 16.36 | 0 | 14.55 | 92.73 | 96.36 | 0 | 0 | 0 | 0 |
| FO_I_ | 6.61 | 0 | 2.89 | 46.28 | 66.94 | 4.96 | 9.09 | 22.73 | 6.2 | 0 | 54.55 | 76.45 | 90.5 | 0 | 0 | 0 | 0 |
| UNC | 6.12 | 0 | 0 | 42.86 | 100 | 0 | 0 | 6.12 | 0 | 0 | 34.69 | 14.29 | 81.63 | 0 | 0 | 0 | 0 |
| TAP | 95.77 | 0 | 0 | 0 | 0 | 0 | 0 | 11.27 | 0 | 0 | 0 | 0 | 0 | 0 | 0 | 0 | 4.23 |
| PCT - bilateral | 0 | 0 | 0 | 0 | 0 | 0 | 0 | 0 | 0 | 0 | 0 | 0 | 0 | 6.56 | 0 | 0 | 0 |
| BCC - bilateral | 3.01 | 0.87 | 0.52 | 0 | 0.06 | 0.06 | 0 | 0 | 0 | 0 | 0 | 0.17 | 0.64 | 0 | 0 | 0 | 3.42 |
| FX - bilateral | 0 | 0 | 0 | 0 | 0 | 0 | 0 | 0 | 0 | 0 | 0 | 0 | 0 | 0 | 0 | 0 | 0 |
| MCP - bilateral | 0 | 0 | 0 | 0 | 0 | 0 | 0 | 0 | 0 | 0 | 0 | 0 | 0 | 0.21 | 0 | 0 | 0 |
| GCC - bilateral | 0.97 | 0.18 | 0 | 0 | 1.33 | 0 | 0 | 0 | 0 | 0 | 0 | 0.53 | 0.09 | 0 | 0 | 0 | 0 |
| SCC - bilateral | 42.32 | 0 | 0 | 0 | 0 | 0 | 0 | 0 | 0 | 0 | 0 | 0 | 0 | 0 | 0 | 3.11 | 3.18 |

**B. Left hemisphere damage (LHD) group (cont.)**

| Areas | 2037 | 2038 | 2039 | **Average** |
| --- | --- | --- | --- | --- |
| Precentral g | 0 | 0 | 0 | **5.55** |
| Superior frontal g | 0 | 0 | 0 | **2.59** |
| Sup. frontal g, orbital | 0 | 0 | 0 | **0.43** |
| Mid. frontal g | 0 | 0 | 0 | **2.26** |
| Mid. frontal g, orbital | 0 | 0 | 0 | **0.00** |
| IFG_OP_ | 0 | 0 | 0 | **7.13** |
| IFG_TRI_ | 0 | 0 | 0 | **5.19** |
| IFG_ORB_ | 0 | 0 | 0 | **1.44** |
| Rolandic op. | 0 | 0 | 0 | **8.96** |
| Supp. motor area | 0 | 0 | 0 | **3.02** |
| Olfactory | 0 | 0 | 0 | **0.76** |
| Sup. frontal g, medial | 0 | 0 | 0 | **1.47** |
| Sup. frontal g, med. orb. | 0 | 0 | 0 | **0.02** |
| Rectus g | 0 | 0 | 0 | **0.71** |
| Insula | 0.81 | 0 | 0.91 | **11.29** |
| Anterior cingulum | 0 | 0 | 0 | **1.53** |
| Middle cingulum | 0 | 0 | 0 | **2.68** |
| Posterior cingulum | 0 | 0 | 0 | **1.95** |
| Hippocampus | 0 | 0 | 0 | **4.93** |
| Parahippocampal g | 0 | 0 | 0 | **3.76** |
| Amygdala | 0 | 0 | 0 | **2.77** |
| Calcarine | 0 | 0 | 0 | **5.34** |
| Cuneus | 0 | 0 | 0 | **2.90** |
| Lingual g | 0 | 0 | 0 | **7.62** |
| Superior occipital g | 0 | 0 | 0 | **2.75** |
| Middle occipital g | 0 | 0 | 0 | **2.30** |
| Inferior occipital g | 0 | 0 | 0 | **4.88** |
| Fusiform g | 0 | 0 | 0 | **5.31** |
| Postcentral g | 0 | 0 | 0 | **5.33** |
| Superior parietal g | 0 | 0 | 0 | **4.76** |
| Inferior parietal g | 0 | 0 | 0 | **7.62** |
| Supramarginal g | 0 | 0 | 0 | **8.55** |
| Angular g | 0 | 0 | 0 | **5.93** |
| Precuneus | 0 | 0 | 0 | **3.24** |
| Paracentral lobule | 0 | 0 | 0 | **4.47** |
| Caudate | 23.49 | 2.91 | 0 | **5.60** |
| Putamen | 62.83 | 9.91 | 0 | **18.14** |
| Pallidum | 54.27 | 0 | 0 | **7.43** |
| Thalamus | 0.27 | 0 | 0 | **2.66** |
| Heschl g | 0 | 0 | 0 | **8.84** |
| Superior temporal g | 0 | 0 | 0 | **5.13** |
| Superior temporal pole | 0 | 0 | 0 | **1.62** |
| Middle temporal g | 0 | 0 | 0 | **1.96** |
| Middle temporal pole | 0 | 0 | 0 | **0.09** |
| Inferior temporal g | 0 | 0 | 0 | **1.77** |
| CST | 0 | 0 | 0 | **0.32** |
| ML | 0 | 0 | 0 | **0.09** |
| ICP | 0 | 0 | 0 | **0.00** |
| SCP | 0 | 0 | 0 | **0.00** |
| CP | 0 | 0 | 0 | **1.11** |
| IC_AL_ | 78.06 | 7.91 | 0.26 | **12.89** |
| IC_PL_ | 9.22 | 22.64 | 0.42 | **16.70** |
| IC_RL_ | 0 | 0 | 0 | **10.75** |
| CR_A_ | 2.2 | 0 | 0 | **8.95** |
| CR_S_ | 0 | 21.86 | 12.45 | **18.71** |
| CR_P_ | 0 | 0.45 | 0 | **14.15** |
| TR_P_ | 0 | 0 | 0 | **7.30** |
| SS | 0 | 0 | 0 | **8.87** |
| External capsule | 32 | 17.33 | 4.67 | **22.44** |
| CGC | 0 | 0 | 0 | **2.96** |
| CGH | 0 | 0 | 0 | **5.54** |
| FX/ST | 0 | 0 | 0 | **6.54** |
| SLF | 0 | 0 | 6.13 | **16.32** |
| FOs | 12.73 | 47.27 | 5.45 | **21.77** |
| FO_I_ | 36.36 | 0 | 0 | **16.79** |
| UNC | 14.29 | 0 | 0 | **12.30** |
| TAP | 0 | 0 | 0 | **7.44** |
| PCT - bilateral | 0 | 0 | 0 | **0.17** |
| BCC - bilateral | 0 | 0 | 0 | **1.40** |
| FX - bilateral | 0 | 0 | 0 | **0.00** |
| MCP - bilateral | 0 | 0 | 0 | **0.01** |
| GCC - bilateral | 0 | 0 | 0 | **1.06** |
| SCC - bilateral | 0 | 0 | 0 | **1.96** |

**Table S13:**

**A. Brain regions in which the percent of subjects who had at least 5% of the region damaged by the stroke was significantly larger in the RHD group compared to the LHD group:**

Rolandic operculum (69% vs. 21%, *p* < .001)

Superior temporal pole (44% vs. 5%, *p* < .001)

Middle temporal gyrus (50% vs. 10%, *p* > .001)

Inferior frontal gyrus, opercular part (59% vs. 18%, *p* < .001)

Heschl gyrus (65% vs. 23%, *p* < .001)

Putamen (78% vs. 38%, *p* < .001)

Inferior frontal gyrus, orbital part (41% vs. 8%, *p* < .001)

Inferior frontal gyrus, triangular part (46% vs. 13%, *p* < .001)

**B. Brain regions in which the average extent of damage (%) was significantly larger in the RHD group compared to the LHD group:**

Rolandic operculum (36% vs. 9%, U = 508.5, *p* < .001)

Heschl gyrus (40% vs. 9%, *U* = 526, *p* < .001)

Inferior frontal gyrus, opercular part (27% vs. 7%, *U* = 540.5, *p* < .001)

Superior temporal pole (16% vs. 2%, *U* = 572.5, *p* < .001)

Superior temporal gyrus (29% vs. 5%, *U* = 545.5, *p* < .001)

Insula (42% vs. 11%, U = 537, *p* < .001)

Inferior frontal gyrus, orbital part (14% vs. 1%, *U* = 586.5, *p* < .001)

Supramarginal gyrus (28% vs. 9%, *U* = 577.5, *p* < .001)

Precentral gyrus (16% vs. 6%, U = 586.5, *p* < .001)

Middle temporal gyrus (16% vs. 2%, *U* = 618, *p* < .001)

Postcentral gyrus (18% vs. 5%, U = 605, *p* < .001)

Inferior frontal gyrus, triangular part (20% vs. 5%, *U* = 631, *p* < .001)

Middle frontal gyrus, orbital part (8% vs. 0%, *U* = 742, *p* < .001)

Middle temporal pole (9% vs. 0%, *U* = 726.5, *p* < .001)

Middle frontal gyrus (11% vs. 2%, *U* = 669.5, *p* < .005)

Angular gyrus (18% vs. 6%, *U* = 677, *p* < .005)

Superior longitudinal fasciculus (43% vs. 16%, U = 537.5, *p* < .001)

External capsule (49% vs. 22%, U = 619, *p* < .005)

Superior corona radiata (37% vs. 19%, U = 635, *p* < .005)
