# Supplementary material for: Brain substrates of visual scene memory: a lesion-behavior mapping study
Source: Front Hum Neurosci. 2025 Sep 1;19:1606051. doi: 10.3389/fnhum.2025.1606051 (PMC12434039; doi:10.3389/fnhum.2025.1606051)
Supplement: Supplementary Table S11 — Demographic and clinical characteristics of individual patients. [file Table_2.docx]

| **Table S11. Demographic and clinical characteristics of individual patients** | | | | | | | | | | | | |  |  |
| --- | --- | --- | --- | --- | --- | --- | --- | --- | --- | --- | --- | --- | --- | --- |
| Participant | Age/  Gender | Education  (years) | Lesion  Type | Lesion  volume (cc) | Registration  Accuracy (%) | TAO  (weeks) | MI |  | SI | VFD | Neglect | Aphasia | Interval between stroke and CT (days) | Interval between CT and test (days) |
|  | **A. Right Hemisphere Damage (RHD)** | | | | | | | | | | |  |  |  |
| 1001 | 40/F | 14 | H | NA | NA | 10 | ++ |  | +/e | −/e | + | - | 32 | 82 |
| 1002* | 61/F | 17 | I | 229.72 | 91.47 | 13 | ++ |  | ++ | −/e | + | - | 0 | 118 |
| 1003 | 29/F | 17 | I | NA | NA | 4 | + |  | + | -/e | + | - | NA | NA |
| 1004 | 63/M | 8 | I | 88.37 | 95.22 | 4 | + |  | +/e | -/e | + | - | 40 | -5 |
| 1005* | 63/F | 12 | H | NA | NA | 3 | + |  | +/e | - | + | - | NA | NA |
| 1006* | 57/M | 15 | I | NA | NA | 6 | ++ |  | + | -/e | - | - | NA | NA |
| 1007 | 76/M | 16 | I | NA | NA | 7 | NA |  | NA | NA | + | - | NA | NA |
| 1008 | 72/M | 16 | I/H | 138.82 | 94.96 | 3 | + |  | +/e | -/e | + | - | 53 | -29 |
| 1009 | 82/F | 15 | NA | NA | NA | 5 | + |  | + | - | + | - | NA | NA |
| 1010 | 66/F | 9 | I | 60.3 | 95.21 | 13 | + |  | + | -/e | + | - | 110 | -15 |
| 1011 | 60/F | 11 | I | 107 | 94.4 | 5 | ++ |  | + | -/e | + | - | 61 | -19 |
| 1012* | 26/F | 12 | AVM-H | NA | NA | 5 | + |  | + | - | - | - | NA | NA |
| 1013 | 58/M | 12 | I | 93.62 | 94.64 | 12 | + |  | + | + | - | - | 35 | 52 |
| 1014 | 60/M | 12 | I | 278 | 91.29 | 9 | + |  | + | -/e | - | - | 5 | 58 |
| 1015 | 62/F | 12 | I | 53.6 | 95.29 | 10 | + |  | + | - | + | - | 53 | 15 |
| 1016 | 63/M | 12 | I | 61.3 | 95.15 | 3 | ++ |  | - | -/e | + | - | 33 | -9 |
| 1017 | 64/M | 10 | I | 47.8 | 94.51 | 5 | + |  | - | -/e | + | - | 44 | -7 |
| 1018 | 44/F | 17 | I/H | 117 | 94.66 | 9 | + |  | + | -/e | + | - | 2 | 60 |
| 1019 | 61/M | 15 | I | 72.3 | 95 | 12 | + |  | + | - | + | - | 55 | 32 |
| 1020 | 60/M | 19 | I | 139 | 94.49 | 5 | - |  | + | - | + | - | 7 | 26 |
| 1021 | 66/M | 15 | H | 24 | 95.18 | 14 | + |  | - | - | + | - | 53 | 114 |
| 1022 | 76/F | 8 | I | 43.5 | 94.58 | 12 | + |  | + | - | + | - | 0 | 84 |
| 1023 | 75/M | 10 | I | 46.4 | 95.33 | 8 | + |  | - | - | - | - | 54 | 0 |
| 1024 | 73/M | 16 | I | 137 | 93.98 | 10 | + |  | - | - | + | - | 243 | -163 |
| 1025 | 79/M | 12 | H | 118 | 94.88 | 8 | + |  | + | -/e | + | - | 53 | 6 |
| 1026* | 40/M | 16 | H | 69.6 | 95.04 | 16 | + |  | - | - | + | - | 49 | 68 |
| 1027 | 79/F | 16 | I | 117 | 94.41 | 12 | + |  | + | - | + | - | 110 | -27 |
| 1028 | 65/M | 10 | H | 137 | 94.16 | 11 | + |  | - | - | + | - | 39 | 35 |
| 1029 | 71/M | 12 | I | 170 | 95.03 | 15 | + |  | ++ | + | + | - | 27 | 64 |
| 1030 | 63/M | 8 | H | 96.6 | 94.19 | 8 | + |  | + | - | + | - | 47 | 24 |
| 1031 | 65/M | 14 | I | 92.4 | 94.31 | 10 | - |  | - | -/e | - | - | 70 | 8 |
| 1032 | 60/M | 18 | I | 52.4 | 94.44 | 9 | + |  | + | -/e | + | - | 54 | 6 |
| 1033* | 57/M | 20 | I/H | 113 | 88.95 | 13 | ++ |  | + | -/e | + | - | 51 | 41 |
| 1034 | 56/F | 18 | I | 24.3 | 94.53 | 6 | + |  | + | -/e | + | - | 36 | 5 |
| 1035 | 66/M | 16 | I | 90.57 | 94.01 | 9 | + |  | - | - | + | - | 54 | 8 |
| 1036 | 28/F | 16 | I | 22.69 | 94.71 | 9 | + |  | - | - | - | - | 27 | 42 |
| 1037 | 58/M | 12 | H | 26.5 | 94.63 | 6 | + |  | - | - | - | - | 1 | 39 |
| 1038 | 43/M | 11 | I | 0.67 | 95.02 | 13 | + |  | - | - | - | - | 3 | 45 |
| 1039 | 57/F | 12 | I | 47.13 | 94.12 | 25 | + |  | - | - | - | - | 49 | 5 |
| 1040 | 60/F | 18 | H | 17.86 | 92.57 | 4 | + |  | + | - | - | - | 35 | -7 |
| 1041* | 72/M | 9 | I | 0.39 | 95.08 | 5 | + |  | + | - | - | - | 27 | 26 |
| 1042* | 55/M | 20+ | I | 5.44 | 94.8 | 7 | + |  | - | - | - | - | 10 | 31 |
| 1043 | 65/M | 17 | I | 3.41 | 93.83 | 3 | + |  | - | - | - | - | 1 | 22 |
| 1044 | 66/F | 14 | H | 25.76 | 94.33 | 4 | + |  | + | + | - | - | 53 | -27 |
| 1045 | 45/F | 14 | I | 125.09 | 94.19 | 8 | + |  | + | - | - | - | 13 | 44 |
| 1046 | 53/M | 12 | H | 10.03 | 94.49 | 6 | + |  | + | - | - | - | 34 | 11 |
| 1047 | 46/M | 12 | I | 41.56 | 95.05 | 4 | - |  | - | - | - | - | 36 | -13 |
| 1048 | 46/M | 16 | H | 22.27 | 93.96 | 5 | + |  | + | - | - | - | 0 | 39 |
| 1049 | 66/M | 11 | I | 2.07 | 94.28 | 8 | + |  | - | - | - | - | 63 | -5 |
| 1050 | 59/M | 9.5 | I | 0.93 | 92.53 | 23 | - |  | - | - | - | - | 88 | 73 |
| 1051 | 74/M | 15 | I | 86.8 | 94.63 | 8 | + |  | + | - | + | - | 4 | 44 |
| 1052 | 60/F | 9 | H | 68.23 | 92.38 | 9 | + |  | + | + | + | - | 47 | 29 |
| 1053 | 60/M | 9 | I | 202.12 | 94.13 | 7.1 | + |  | + | - | + | - | 49 | 1 |
| 1054 | 74/M | 16 | I | 5.99 | 94.47 | 13 | + |  | + | - | - | - | 1 | 25 |
|  |  |  |  |  |  |  |  |  |  |  |  |  |  |  |

| Participant | Age/  Gender | Education  (years) | Lesion  type | Lesion  volume (cc) | Registration  Accuracy (%) | TAO  (weeks) | MI |  | SI | VFD | Neglect | | Aphasia | | Interval between stroke and CT (days) | | Interval between CT and test (days) | |
| --- | --- | --- | --- | --- | --- | --- | --- | --- | --- | --- | --- | --- | --- | --- | --- | --- | --- | --- |
|  | **B. Left Hemisphere Damage (LHD)** | | | | | | | | | | |  | |  | |  | |  |
| 2001 | 43/M | 15 | H | 58.1 | NA | 12 | + |  | + | - | - | | - | | 60 | | 27 | |
| 2002 | 77/F | 13 | I | 45.5 | NA | 12 | + |  | - | - | - | | + | | NA | | NA | |
| 2003 | 32/F | 12 | AVM-H | NA | NA | 4 | - |  | + | - | - | | - | | 30 | | 9 | |
| 2004 | 32/F | 16 | CVST-H | 6.1 | NA | 5 | - |  | - | - | - | | - | | NA | | NA | |
| 2005 | 27/F | 13 | CVST-H | 112 | NA | 15 | ++ |  | + | - | - | | - | | 19 | | 278 | |
| 2006 | 75/F | 12 | I | 9.08 | 94.44 | 12 | + |  | + | - | - | | + | | 76 | | 7 | |
| 2007 | 57/M | 12 | I | 10.4 | 94.45 | 4 | - |  | + | - | - | | + | | 21 | | 7 | |
| 2008* | 65/M | 20 | I | 22.9 | 94.42 | 3 | - |  | + | - | - | | + | | 44 | | -14 | |
| 2009 | 58/M | 15 | I | 67.7 | 95.21 | 11 | + |  | + | - | - | | + | | 47 | | 39 | |
| 2010 | 70/F | 16 | I/H | 34.3 | 93.9 | 9 | + |  | - | - | - | | + | | 29 | | 51 | |
| 2011 | 81/F | 8 | I | 12.5 | 94.67 | 7 | - |  | - | - | - | | + | | 44 | | 9 | |
| 2012 | 67/F | 16 | I | 14.2 | 94.5 | 5 | + |  | - | - | - | | - | | 36 | | 5 | |
| 2013 | 67/M | 19 | I | 57.8 | 93.96 | 8 | + |  | + | - | - | | + | | 59 | | 0 | |
| 2014 | 54/M | 12 | I | 41.75 | 95.13 | 3 | + |  | - | + | - | | - | | 41 | | 11 | |
| 2015 | 62/F | 12 | I | 28.45 | 95.16 | 6 | + |  | - | - | - | | - | | 42 | | 8 | |
| 2016 | 52/F | 12 | I | 21.8 | 94.35 | 10 | + |  | + | - | - | | + | | 54 | | 16 | |
| 2017 | 54/M | 10 | I | 35.5 | 95.12 | 6 | + |  | + | + | - | | - | | 97 | | -53 | |
| 2018 | 48/M | 12 | I | 21.19 | 94.59 | 5 | + |  | - | - | - | | + | | 47 | | -1 | |
| 2019 | 70/F | 12 | I | 1.89 | 94.17 | 9 | + |  | - | - | - | | + | | 71 | | -12 | |
| 2020 | 65/M | 12 | I | 123.19 | 94.68 | 6 | + |  | + | - | - | | - | | 2 | | 46 | |
| 2021 | 75/M | 11 | I | 52.12 | 93.17 | 7 | - |  | - | - | - | | + | | 37 | | -1 | |
| 2022 | 60/M | 16 | I | 4.02 | 90.42 | 4 | + |  | - | - | - | | + | | 40 | | 46 | |
| 2023 | 46/M | 11 | H | 11.61 | 94.25 | 6 | + |  | - | - | - | | - | | 48 | | 63 | |
| 2024* | 70/F | 15 | I | 49.61 | 92.42 | 11.5 | + |  | - | - | - | | - | | 1 | | 192 | |
| 2025 | 59/F | 10 | I | 8.45 | 94.86 | 8.6 | + |  | + | - | - | | - | | 3 | | 58 | |
| 2026 | 49/M | 14 | H | 9.13 | 93.57 | 5.6 | + |  | + | - | - | | + | | 71 | | -31 | |
| 2027 | 49/M | 9 | I | 12.29 | 93.86 | 8.4 | + |  | + | - | - | | + | | 3 | | 58 | |
| 2028 | 60/M | 12 | I | 3.25 | 94.38 | 6 | + |  | + | + | - | | - | | 32 | | 17 | |
| 2029 | 60/F | 12 | I | 0.84 | 91.08 | 10 | + |  | - | - | - | | - | | 49 | | 29 | |
| 2030 | 65/M | 16 | I | 11.82 | 94.19 | 10.6 | + |  | - | - | - | | - | | 51 | | 30 | |
| 2031 | 39/M | 14 | H | 21.72 | 95.57 | 13 | + |  | + | - | - | | + | | 35 | | -156 | |
| 2032 | 74/M | 15 | H | 55.91 | 93.16 | 8.2 | + |  | + | + | - | | + | | 36 | | 21 | |
| 2033 | 58/M | 12 | I | 0.41 | 94.32 | 6.4 | + |  | - | - | - | | - | | 39 | | 8 | |
| 2034 | 66/F | 8 | I | 2.95 | 93.86 | 4.6 | + |  | + | + | - | | - | | 42 | | -7 | |
| 2035 | 70/M | 8 | I | 25.98 | 95.17 | 2.4 | + |  | - | + | - | | - | | 0 | | 28 | |
| 2036 | 79/M | 10 | H | 42.17 | 94.09 | 2.3 | + |  | + | + | - | | + | | 52 | | -35 | |
| 2037 | 35/F | 12 | H | 8.78 | 94.52 | 9 | + |  | + | + | - | | - | | 22 | | 37 | |
| 2038 | 67/M | 12 | I | 3.28 | 94.1 | 8 | + |  | + | - | - | | - | | 65 | | -6 | |
| 2039 | 72/F | 10 | I | 1.18 | 94.2 | 20 | - |  | - | - | - | | - | | 7 | | 54 | |

* = left handedness (all other patients were right handers); I/H = ischemic/hemorrhagic stroke; CVST = cerebral venous sinus thrombosis; AVM = arterial venous malformation; TAO = time of memory testing after stroke onset; MI/SI = motor/sensory impairment (- = no impairment, + = mild impairment, ++ = moderate/severe impairment); VFD = visual field defect (- = no, -/e = extinction upon bilateral simultaneous stimulation but no VFD); NA = data not available.
